# OCTN2 Activates a Non‐Canonical Carnitine Metabolic Pathway to Promote MASH‐HCC Progression and Immunotherapy Resistance

**DOI:** 10.1002/advs.202517054

**Published:** 2026-01-21

**Authors:** Chuqi Xia, Xiao Zhang, Jinze Li, Ning Xu, Sheng Hu, Qiyu Lu, Yuxuan Li, Taifu Xiao, Xu Li, Xue Wang, Kequan Xu, Daoming Liang

**Affiliations:** ^1^ Department of Gastrointestinal Surgery The Second Affiliated Hospital of Kunming Medical University Kunming China; ^2^ TUM School of Medicine and Health Technical University of Munich Munich Germany; ^3^ Division of Liver Surgery, Department of General Surgery, West China Hospital Sichuan University Chengdu China; ^4^ Department of Hepatobiliary Surgery The Second Affiliated Hospital of Kunming Medical University Kunming China; ^5^ Department of Gastrointestinal Surgery The Second Affiliated Hospital of Nanchang University Nanchang China; ^6^ Zhongnan Hospital Wuhan University Wuhan China

**Keywords:** metabolic dysfunction‐associated steatohepatitis (MASH), hepatocellular carcinoma (HCC), L‐carnitine, OCTN2, acetyl group buffering

## Abstract

Metabolic dysfunction‐associated steatohepatitis related hepatocellular carcinoma (MASH‐HCC) is a distinct HCC subtype characterized by lipid accumulation, impaired fatty acid oxidation (FAO), immune evasion, and resistance to immunotherapy. In this study, we observed elevated levels of L‐carnitine—a classical FAO activator—and its transporter OCTN2 in MASH‐HCC. Mechanistically, L‐carnitine is redirected from FAO promotion to buffering intracellular acetyl groups via conversion to acetyl‐L‐carnitine, leading to acetyl group depletion. This disrupts protein acetylation through two distinct pathways: reduced acetylation of p53 weakens its tumor‐suppressive signaling and promotes tumor progression, while decreased acetylation of histone H3 impairs MHC‐I antigen presentation, facilitating immune evasion. We further identified that the lncRNA LINCMD1 competitively bound the E3 ligase DZIP3, sequestering it in the nucleus and preventing its interaction with cytoplasmic OCTN2. This inhibited K48‐linked ubiquitination of OCTN2 and stabilized its protein expression, further amplifying L‐carnitine accumulation. To therapeutically target this axis, we developed a liver‐specific lipid nanoparticle (LNP)‐delivered antisense oligonucleotide against the DZIP3‐binding region of LINCMD1, which restored p53 and MHC‐I pathways and enhanced anti–PD‐1 efficacy in vivo. Together, our findings uncover a noncanonical carnitine‐driven metabolic–epigenetic–immune bypass in MASH‐HCC and identify the LINCMD1/DZIP3/OCTN2–L‐carnitine axis as a potential therapeutic target.

## Graphical abstract

In non‐MASH‐HCC, L‐carnitine promotes tumor progression primarily through its classical role in enhancing fatty acid oxidation (FAO). However, in MASH‐HCC, where FAO is markedly suppressed, L‐carnitine shifts from this canonical function to serve instead as an intracellular acetyl group buffer. Elevated L‐carnitine in MASH‐HCC binds acetyl groups to form acetyl‐L‐carnitine, resulting in acetyl group depletion. This leads, on one hand, to reduced acetylation and decreased stability of the tumor suppressor p53, thereby compromising its tumor‐suppressive function and facilitating tumor progression. On the other hand, loss of histone H3 acetylation reduces activity, resulting in suppression of MHC‐I antigen presentation, which ultimately promotes immune evasion and resistance to immunotherapy. Mechanistically, the lncRNA LINCMD1 is highly expressed and predominantly localized in the nucleus of MASH‐HCC cells, where it binds to the E3 ubiquitin ligase DZIP3. This interaction retains DZIP3 within the nucleus, thereby reducing its interaction with the cytoplasmic carnitine transporter OCTN2, and inhibiting DZIP3‐mediated K48‐linked ubiquitination and proteasomal degradation of OCTN2. As a result, OCTN2 is stabilized and upregulated, leading to increased intracellular L‐carnitine accumulation and reinforcement of this non‐canonical metabolic pathway. (Created with bioRender.com)

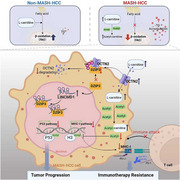

**Abbreviations**
Acetyl‐CoA: Acetyl coenzyme A
ASO: Antisense oligonucleotide
BioGRID: Biological General Repository for Interaction Datasets
DZIP3: DAZ interacting zinc finger protein 3
FAO: Fatty acid oxidation
GEPIA: Gene Expression Profiling Interactive Analysis
GSEA: Gene Set Enrichment Analysis
HCC: Hepatocellular carcinoma
IHC: Immunohistochemistry
iUUCD: Integrative Ubiquitin and Ubiquitin‐like Conjugation Database
IntAct: IntAct Molecular Interaction Database
IP/MS: Immunoprecipitation–mass spectrometry
KEGG: Kyoto Encyclopedia of Genes and Genomes
LINCMD1: Long intergenic non‐coding RNA MD1
LNP: Lipid nanoparticle
MASH: Metabolic dysfunction‐associated steatohepatitis
MHC‐I: Major histocompatibility complex class I
OCTN2: Organic cation/carnitine transporter 2
PD‐1: Programmed cell death protein 1
qPCR: Quantitative polymerase chain reaction
RF: Random Forest
RIP: RNA immunoprecipitation
STRING: Search Tool for the Retrieval of Interacting Genes/Proteins
SVM: Support Vector Machine
TME: Tumor microenvironment

## 1 Introduction

Hepatocellular carcinoma (HCC) arising from metabolic dysfunction‐associated steatohepatitis (MASH) is increasingly recognized as a distinct and growing subtype of HCC that differs markedly from other HCC in its metabolic microenvironment and tumorigenic pathways [1]. Unlike other HCC, which typically progresses in an inflammatory and fibrotic milieu driven by viral replication or excessive alcohol consumption, MASH‐HCC is shaped by metabolic stress, lipotoxicity, and a unique immunosuppressive tumor microenvironment [2]. These differences manifest in reprogrammed lipid metabolism, altered nutrient utilization, and immune modulation that collectively drive tumor growth [3]. Notably, the changes in the lipid microenvironment profoundly affect immune cell recruitment and activation, contributing to impaired antigen presentation and T cell dysfunction [4]. Consistent with these observations, large clinical datasets have shown that patients with MASH‐HCC derive minimal survival benefit from PD‐1/PD‐L1 inhibitors compared with those with viral HCC [5]. Despite advances in understanding some of these metabolic shifts, the detailed metabolic adaptations supporting MASH‐HCC and their impact on immunotherapy resistance remain incompletely characterized.

Among the various metabolic pathways dysregulated in other HCC, fatty acid oxidation (FAO) has been recognized as a key driver of tumor progression, often facilitated by the mitochondrial carnitine shuttle system [6]. This system relies on L‐carnitine to transport long‐chain fatty acids into the mitochondria for β‐oxidation, thereby fueling ATP production and sustaining cancer cell survival [7], and the activation of carnitine transport is a key driver of enhanced FAO in other HCC [8]. However, consistent with findings from González‐Romero, Huang, and others–as well as our own previous work–FAO is markedly suppressed in MASH‐HCC, which may partially account for the substantial lipid accumulation observed in its tumor microenvironment [9, 10, 11, 12]. Notably, Fujiwara et al. further demonstrated that this FAO suppression leads to increased carnitine accumulation in MASH‐HCC tissues [13]. Building on this, our study further revealed a striking activation of carnitine uptake mechanisms in MASH‐HCC, suggesting that L‐carnitine accumulation is not merely a passive consequence of impaired FAO, but may reflect a distinct metabolic adaptation. These findings imply that L‐carnitine may exert functions beyond its classical role in facilitating FAO, instead promoting tumor adaptation and progression under FAO‐deficient conditions. Nonetheless, the precise biological significance of this aberrant carnitine transport in MASH‐HCC remains unclear and warrants further investigation.

To address the noncanonical activation of carnitine metabolism in MASH‐HCC, we systematically investigated the underlying mechanisms. L‐carnitine classically transports long‐chain fatty acids into mitochondria for β‐oxidation; however, it also acts as an acceptor of short‐chain acyl groups, particularly acetyl groups, contributing to acetyl group buffering [14, 15]. Our study revealed that in MASH‐HCC, accumulated L‐carnitine primarily supports acetyl group buffering through the formation of acetyl‐L‐carnitine (ALCAR) rather than promoting FAO. This buffering depletes intracellular acetyl groups essential for protein acetylation, resulting in reduced p53 acetylation, which suppresses its tumor‐suppressive signaling and promotes tumor progression. Simultaneously, decreased histone H3 acetylation impairs MHC‐I antigen presentation, thereby facilitating immune evasion. Mechanistically, lncRNA LINCMD1 competitively binds to the E3 ubiquitin ligase DZIP3, sequestering it in the nucleus and thereby preventing it from mediating K48‐linked ubiquitination of the L‐carnitine transporter OCTN2, ultimately leading to enhanced intracellular accumulation of L‐carnitine. Given that immunotherapy for MASH‐HCC suffers from multiple limitations, including poor efficacy, low response rates, and a high propensity for resistance [5], we further designed a lipid nanoparticle (LNP)‐based delivery system targeting this metabolic axis. This approach sensitized tumors to anti–PD‐1 therapy and significantly attenuated tumor progression. Collectively, these findings reveal a metabolic bypass in MASH‐HCC that shifts carnitine metabolism from FAO toward acetyl group homeostasis, uncovering a previously unrecognized metabolic‐epigenetic‐immune circuit and highlighting a promising therapeutic target.

## 2 Results

### 2.1 Elevated Carnitine Transport Identified as a Novel Metabolic Feature in MASH‐HCC

**FIGURE 1:**
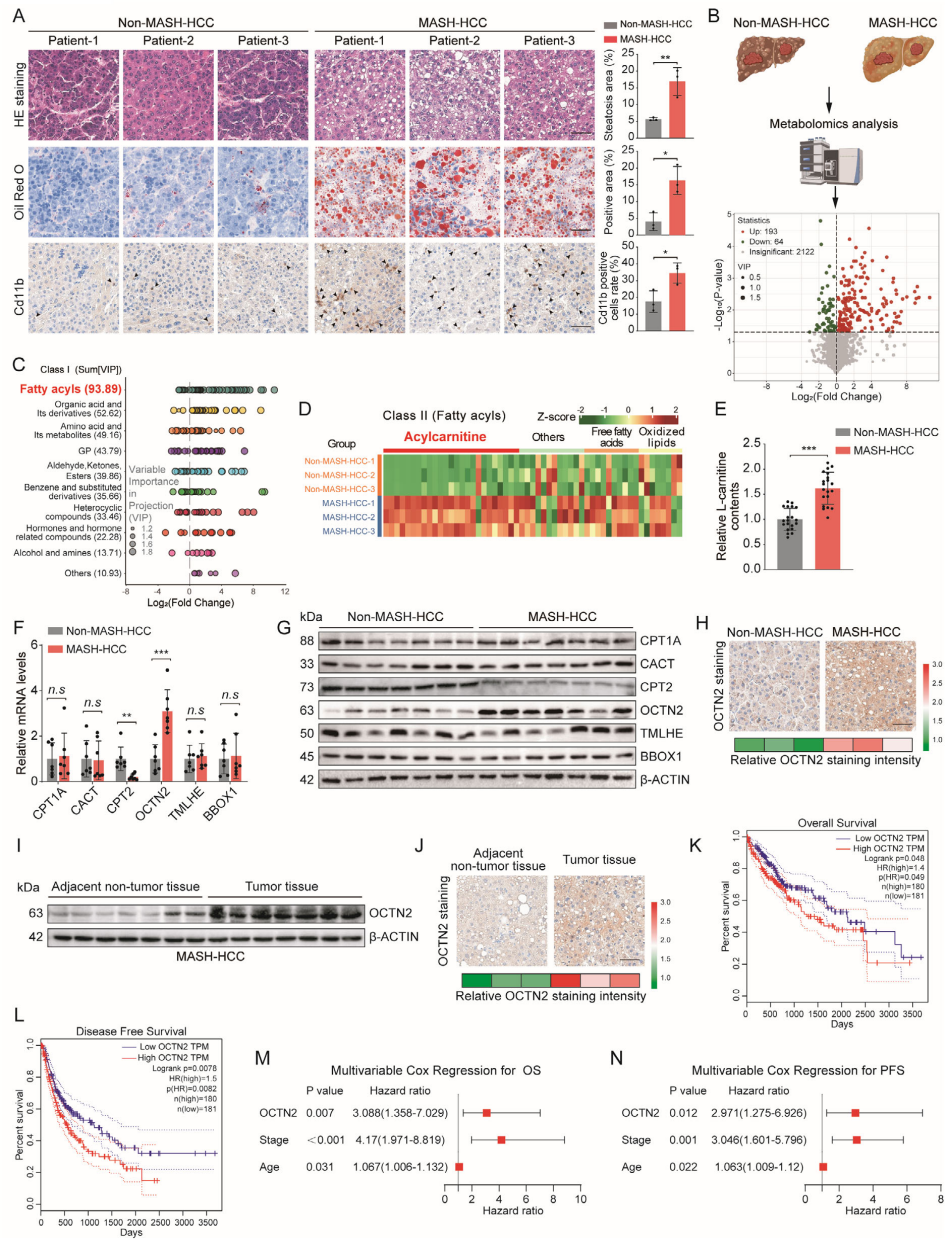
Elevated Carnitine Transport Identified as a Novel Metabolic Feature in MASH‐HCC. (A) Representative H&E, Oil Red O, and Cd11b staining images with corresponding quantitative analyses in tumor tissues from patients with MASH‐HCC and non‐MASH‐HCC. Scale bars, 50 µm. (B) Volcano plot showing significantly altered metabolites between MASH‐HCC and non‐MASH‐HCC tumor samples (n = 3 per group). (C) Scatter plot depicting differential metabolites classified according to Class I categories between MASH‐HCC and non‐MASH‐HCC tumor samples (n = 3 per group). (D) Heatmap of differentially expressed fatty acyl metabolites between MASH‐HCC and non‐MASH‐HCC tumor samples (n = 3 per group). (E) Quantification of L‐carnitine levels in tumor tissues from MASH‐HCC and non‐MASH‐HCC patients (n = 20 per group). (F) qRT‐PCR analysis of mRNA expression levels of carnitine metabolism–related genes in MASH‐HCC and non‐MASH‐HCC patient samples (n = 7 per group). (G) Western blot analysis of carnitine metabolism–related proteins in MASH‐HCC and non‐MASH‐HCC tissues (n = 7 per group). (H) Representative immunohistochemical staining of OCTN2 in clinical tumor tissues from MASH‐HCC and non‐MASH‐HCC patients (n = 3 per group). Scale bars, 50 µm. (I) Western blot analysis of OCTN2 protein expression in paired tumor and adjacent non‐tumor tissues from MASH‐HCC patients (n = 7 per group). (J) Representative immunohistochemical staining of OCTN2 in paired tumor and adjacent non‐tumor tissues from MASH‐HCC patients (n = 3 per group). Scale bars, 50 µm. (K,L) Kaplan–Meier survival curves showing overall survival (K) and disease‐free survival (L) of HCC patients stratified by high vs. low OCTN2 expression. (M,N) Multivariate Cox regression analyses evaluating OCTN2 expression, tumor stage, and age with overall survival (OS) (M) and progression‐free survival (PFS) (N) in the MASH‐HCC cohort (n = 41). The data are expressed as the mean ± SD. *p*‐values were determined by a two‐tailed Student's *t*‐test. Statistical significance: *n.s* means not significant, ^*^ *p* < 0.05, ^**^ *p* < 0.01, ^***^ *p* < 0.001.

First, we analyzed the clinical and pathological characteristics of 41 MASH‐HCC and 22 non‐MASH‐HCC patients (Tables S1 and S2). Consistent with previous reports [1, 2, 3, 4, 6], MASH‐HCC exhibited more pronounced hepatocellular ballooning, lipid accumulation, and inflammatory infiltration than non‐MASH‐HCC tissues (Figure 1A). To explore metabolic alterations associated with MASH‐HCC, we performed untargeted metabolomic profiling on tumor tissues from three MASH‐HCC and three non‐MASH‐HCC patients. Multivariate statistical analyses indicated low within‐group variability and clear group separation (Figure S1A,B). Volcano plot analysis identified numerous differentially expressed metabolites between the two groups (Figure 1B; Table S3). Metabolites classification demonstrated that, in agreement with prior studies [16], lipid metabolites constituted a major portion of the altered metabolites, with fatty acyl subclass being particularly prominent (Figure 1C; Figure S1C). Among these, oxidized lipids and free fatty acids—well‐established contributors to MASH‐HCC progression [6]—were elevated; interestingly, we also observed a marked increase in carnitine‐related metabolites, a feature less frequently highlighted in the literature (Figure 1D). Since carnitine is classically known to promote fatty acid oxidation (FAO) [8, 17], its accumulation in the context of suppressed FAO in MASH‐HCC prompted us to investigate its underlying regulatory mechanisms. We therefore focused on L‐carnitine, the fundamental form within the carnitine family, while other derivatives are typically produced through its chemical modification or metabolic conversion [18]. Quantification of L‐carnitine in clinical liver tissues revealed significantly higher levels in MASH‐HCC than in non‐MASH‐HCC (Figure 1E). Using a validated mouse model of MASH‐HCC (Figure S1D), we similarly detected elevated L‐carnitine levels in tumor tissues (Figure S1E).

To investigate the underlying regulatory mechanisms, we examined genes involved in carnitine biosynthesis (TMLHE and BBOX1), the carnitine–acylcarnitine shuttle (CPT1A, CACT, CPT2), and carnitine transport (OCTN2). mRNA and protein levels of TMLHE, BBOX1, CPT1A, and CACT were comparable between the two groups (Figure 1F,G). Consistent with previous findings [13], CPT2 was significantly downregulated in MASH‐HCC. Notably, the carnitine transporter OCTN2 showed a striking increase: OCTN2 mRNA levels were ∼2‐fold higher and protein levels ∼10‐fold higher in MASH‐HCC compared with non‐MASH‐HCC (Figure 1F,G; Figure S1F,G), suggesting a potentially critical role for OCTN2 in MASH‐HCC progression. We therefore further assessed OCTN2 expression. IHC staining demonstrated markedly increased OCTN2 protein in MASH‐HCC tumors (Figure 1H). qPCR, Western blotting, and IHC collectively confirmed that OCTN2 expression is significantly higher in tumor tissues than in adjacent non‐tumor tissues from MASH‐HCC patients (Figure 1I,J; Figure S1H). Likewise, in the MASH‐HCC mouse model, OCTN2 mRNA and protein levels were elevated by approximately twofold and eightfold, respectively, compared with non‐MASH‐HCC mice (Figure S1I,J). Large‐cohort bioinformatic analyses further demonstrated that high OCTN2 expression is significantly associated with poor overall survival (OS) and disease‐free survival (DFS) in HCC (Figure 1K,L). Finally, in our 41‐case MASH‐HCC cohort, univariate and multivariate Cox regression analyses evaluating the relationships of age, sex, BMI, diabetes, hypertension, and OCTN2 expression with OS and PFS revealed that OCTN2 serves as an independent prognostic predictor for both endpoints (Tables S4 and S5; Figure 1M,N). In summary, these findings identify enhanced carnitine transport as a distinct metabolic feature of MASH‐HCC and highlight OCTN2 as a critical driver of MASH‐HCC progression.

### 2.2 OCTN2 Promotes MASH‐HCC Progression via a Non‐Canonical Carnitine Metabolic Pathway

To recapitulate hepatic lipid accumulation and steatosis observed in vivo while concurrently modeling the inflammatory milieu, we established an in vitro MASH‐HCC model by treating HuH‐7 cells with palmitic acid (PA, 0.4 mM), oleic acid (OA, 0.8 mM), along with IL‐6 (10 ng/mL), TNF‐α (10 ng/mL), and LPS (100 ng/mL) as previously described [19]. Under this metabolic stimulation, BODIPY staining and qPCR analysis revealed markedly increased lipid accumulation and inflammatory gene expression in HuH‐7 cells (Figure S2A,B), confirming successful model establishment. Consistent with previous findings [9, 10, 11, 12], fatty acid β‐oxidation (FAO) activity was significantly suppressed in MASH‐HCC cells relative to non‐MASH‐HCC cells (Figure S2C). Concurrently, immunofluorescence staining demonstrated a pronounced increase in OCTN2 expression in MASH‐HCC cells (Figure S2D).

**FIGURE 2:**
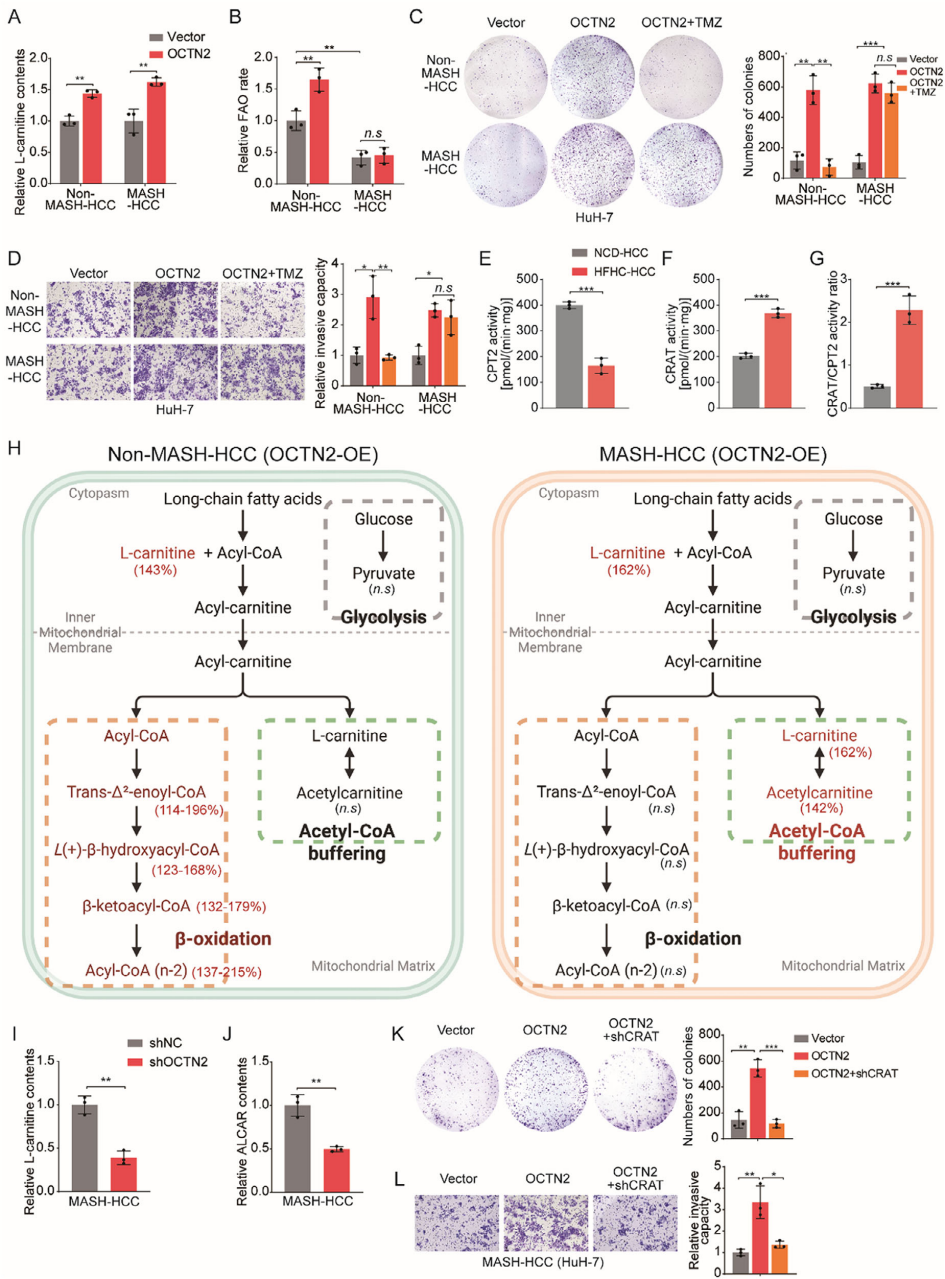
OCTN2 Promotes MASH‐HCC Progression via a Non‐canonical Carnitine Metabolic Pathway. (A) Quantification of relative L‐carnitine levels in non‐MASH‐HCC and MASH‐HCC cells transfected with control vector or OCTN2 plasmids (n = 3 per group). (B) Fatty acid oxidation (FAO) capacity in non‐MASH‐HCC and MASH‐HCC cells following transfection with empty vector or OCTN2 plasmid (n = 3 per group). (C) Colony formation assays evaluating the proliferative capacity of MASH‐HCC cells (PA/OA and IL‐6/TNF‐α/LPS–treated HuH‐7 cells) and non‐MASH‐HCC cells transfected with vector or OCTN2, with or without trimetazidine (TMZ, 1 mM) (n = 3 per group). (D) Transwell Matrigel invasion assays assessing invasive capacity under the same conditions as in panel C (n = 3 per group). (E) Mitochondrial CPT2 enzymatic activity was measured in liver tumors from NCD‐HCC and HFHC‐HCC mice (n = 3 per group). (F) Mitochondrial CRAT enzymatic activity was measured in liver tumors from NCD‐HCC and HFHC‐HCC mice (n = 3 per group). (G) CRAT/CPT2 activity ratio in liver tumors from NCD‐HCC and HFHC‐HCC mice (n = 3 per group). (H) Schematic diagram illustrating metabolic pathway alterations in non‐MASH‐HCC and MASH‐HCC cells upon OCTN2 overexpression, including changes in Trans‐Δ^2^‐enoyl‐CoA, L (+)‐β‐hydroxyacyl‐CoA, β‐ketoacyl‐CoA, acetyl‐L‐carnitine (ALCAR), and pyruvate levels (n = 3 per group). (I,J) Intracellular L‐carnitine and acetyl‐L‐carnitine (ALCAR) levels in MASH‐HCC cells transfected with shNC or shOCTN2 plasmids (n = 3 per group). (K,L) Colony formation and Transwell invasion assays evaluating proliferation and invasiveness in MASH‐HCC cells transfected with vector, OCTN2, or OCTN2 + shCRAT plasmids (n = 3 per group). The data are expressed as the mean ± SD. *p*‐values were determined by two‐tailed Student's *t*‐test (A, B, E, F, G, I, J) or one‐way ANOVA followed by a post hoc Tukey test (C, D, K, L). Statistical significance: *n.s* means not significant, ^*^ *p* < 0.05, ^**^ *p* < 0.01, ^***^ *p* < 0.001.

Previous studies have shown that OCTN2 enhances non‐MASH‐HCC progression by promoting FAO through increased L‐carnitine uptake [8]. However, this contradicts our observation of elevated OCTN2 expression alongside suppressed FAO in MASH‐HCC. To address this, we overexpressed OCTN2 in MASH‐HCC cells and observed a significant increase in intracellular L‐carnitine levels (Figure 2A; Figure S2E). Surprisingly, despite the L‐carnitine elevation, OCTN2 overexpression failed to restore FAO activity in MASH‐HCC cells, in contrast to its FAO‐promoting effect in non‐MASH‐HCC cells (Figure 2B). Functional assays further showed that OCTN2 overexpression significantly enhanced proliferation, migration, and invasion in both non‐MASH‐HCC and MASH‐HCC cells. However, treatment with the FAO inhibitor trimetazidine (TMZ) abrogated these tumor‐promoting effects in non‐MASH‐HCC cells but not in MASH‐HCC cells (Figure 2C,D; Figure S2E). These findings suggest that although OCTN2 increases intracellular L‐carnitine levels in MASH‐HCC, its oncogenic effects are likely mediated through FAO‐independent mechanisms due to the inherent suppression of FAO in this tumor context [13].

L‐carnitine normally facilitates the mitochondrial import of long‐chain fatty acids for β‐oxidation and also plays a role in acetyl group buffering and detoxification [20]. We therefore measured the enzymatic activities of CPT2 (the key enzyme for FAO) and CRAT (the central enzyme for acetyl‐group buffering) in liver tumors from MASH‐HCC and non‐MASH‐HCC mice. Mitochondria isolated from tumor tissues were subjected to LC–MS‐based quantification of reaction products. CPT2 activity was markedly reduced in MASH‐HCC mitochondria, whereas CRAT activity was significantly increased, resulting in a dramatically elevated CRAT/CPT2 activity ratio (Figure 2E–G). These findings indicate suppressed FAO in MASH‐HCC and enhanced conversion of acetyl‐CoA to acetyl‐L‐carnitine (ALCAR) to support acetyl‐group buffering. To further delineate the metabolic pathways involving L‐carnitine in MASH‐HCC, we performed LC–MS/MS‐based metabolomic profiling in non‐MASH‐HCC and MASH‐HCC cells overexpressing OCTN2. The results showed that β‐oxidation‐related metabolites were significantly elevated in non‐MASH‐HCC cells but remained unchanged in MASH‐HCC cells. In contrast, the acetyl‐group buffering product ALCAR was markedly increased only in MASH‐HCC cells, whereas pyruvate, the end product of glycolysis, showed no significant changes in either cell type (Figure 2H). Moreover, OCTN2 knockdown significantly reduced L‐carnitine and ALCAR levels in MASH‐HCC cells (Figure 2I,J; Figure S2G). Importantly, CRAT knockdown abrogated the OCTN2 overexpression–induced increases in MASH‐HCC cell proliferation, migration, and invasion (Figure 2K,L; Figure S2H,I).

Collectively, these findings support a model in which, under conditions of FAO suppression, OCTN2 drives MASH‐HCC progression through a non‐canonical carnitine metabolic pathway characterized by enhanced acetyl‐group buffering rather than β‐oxidation, thereby promoting tumor growth and aggressiveness.

### 2.3 OCTN2 Inhibits P53 Acetylation to Promote MASH‐HCC Progression

**FIGURE 3:**
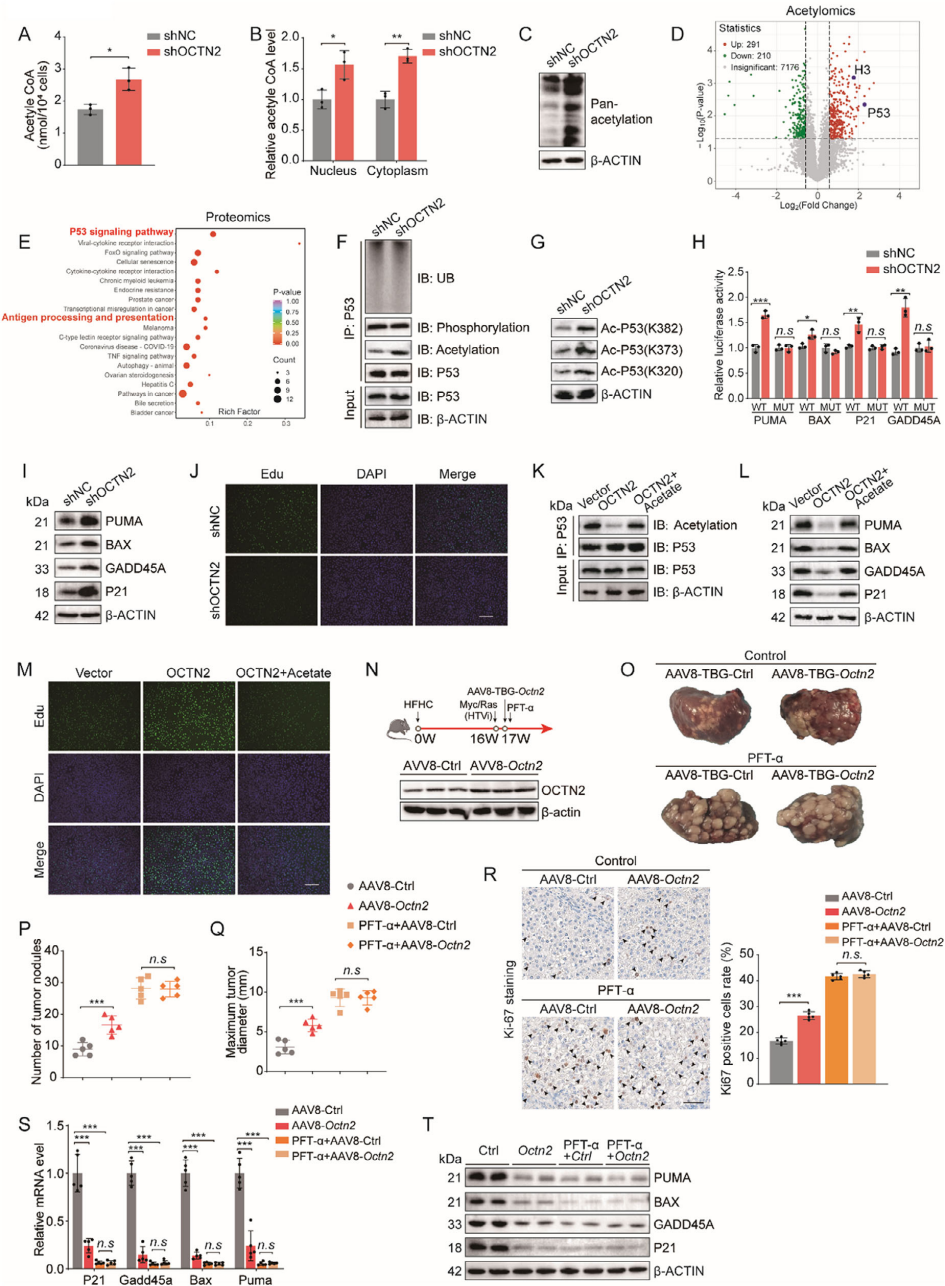
OCTN2 inhibits p53 acetylation to promote MASH‐HCC progression. (A) Quantification of intracellular acetyl‐CoA levels in MASH‐HCC cells transfected with shNC or shOCTN2 plasmids (n = 3 per group). (B) Relative acetyl‐CoA levels in nuclear and cytoplasmic fractions of MASH‐HCC cells transfected with shNC or shOCTN2 plasmids (n = 3 per group). (C) Western blot analysis of pan‐acetylation in MASH‐HCC cells transfected with shNC or shOCTN2 plasmids (n = 3 per group). (D) Volcano plot of acetylomics data showing differentially expressed proteins in MASH‐HCC cells transfected with shNC or shOCTN2 plasmids (n = 3 per group). (E) KEGG pathway enrichment analysis based on proteomics data of MASH‐HCC cells transfected with shNC or shOCTN2 plasmids (n = 3 per group). (F) Western blot analysis of total protein, and acetylated, phosphorylated, and ubiquitinated p53 in MASH‐HCC cells transfected with shNC or shOCTN2 plasmids (n = 3 per group). (G) Western blot analysis of p53 acetylation at lysine 320 (K320), lysine 373 (K373), and lysine 382 (K382) in MASH‐HCC cells transfected with shNC or shOCTN2 plasmids (n = 3 per group). (H) Dual‐luciferase reporter assays measuring transcriptional activity of p53 target genes (P21, GADD45A, BAX, PUMA) in MASH‐HCC cells transfected with shNC or shOCTN2 plasmids (n = 3 per group). (I) Western blot analysis of p53 downstream target proteins (P21, GADD45A, BAX, PUMA) in MASH‐HCC cells transfected with shNC or shOCTN2 plasmids (n = 3 per group). (J) EdU incorporation assays showing proliferation rates of MASH‐HCC cells transfected with shNC or shOCTN2 plasmids (n = 3 per group). Scale bars, 50 µm. (K) Western blot analysis of p53 acetylation in MASH‐HCC cells transfected with vector, OCTN2 plasmids, or OCTN2 plasmids plus sodium acetate (5 mM) (n = 3 per group). (L) Western blot analysis of p53 downstream targets (P21, GADD45A, BAX, PUMA) in MASH‐HCC cells transfected with vector, OCTN2 plasmids, or OCTN2 plasmids plus sodium acetate (5 mM) (n = 3 per group). (M) EdU incorporation assays showing proliferation rates in MASH‐HCC cells transfected with vector, OCTN2, or OCTN2 plus sodium acetate (5 mM) (n = 3 per group). Scale bars, 50 µm. (N) Schematic overview of the generation of a hepatocyte‐specific AAV8‐TBG‐*Octn2* MASH‐HCC mouse model and the administration schedule of the p53 inhibitor PFT‐α. (O) Representative liver images from HFHC‐HCC mice injected with AAV8‐TBG‐Ctrl or AAV8‐TBG‐*Octn2*, with or without PFT‐α treatment (n = 5 per group). (P,Q) Quantification of tumor nodule number and maximum tumor diameter in HFHC‐HCC mice from the same treatment groups as in O (n = 5 per group). (R) Ki67 immunostaining showing tumor cell proliferation in liver tumors from the same groups as in O (n = 5 per group). Scale bars, 50 µm. (S,T) qRT‐PCR and Western blot analysis of p53 downstream targets mRNA and protein level in liver tumor tissues from the same groups as in O (n = 5 per group). The data are expressed as the mean ± SD. *p*‐values were determined by two‐tailed Student's *t*‐test (A, B, H) or one‐way ANOVA followed by a post hoc Tukey test (P, Q,R, S). Statistical significance: n.s. means not significant, ^*^ *p* < 0.05, ^**^ *p* < 0.01, ^***^ *p* < 0.001.

The primary function of the acetyl group buffering system is to maintain the dynamic balance of intracellular acetyl‐CoA levels [14]. Excess acetyl‐CoA can be temporarily stored by forming ALCAR with L‐carnitine, preventing metabolic imbalance and supporting essential cellular functions [21]. We therefore measured acetyl‐CoA levels in MASH‐HCC cells and found that OCTN2 overexpression caused a marked reduction in acetyl‐CoA, whereas OCTN2 knockdown significantly increased acetyl‐CoA levels (Figure 3A; Figure S3A). Subcellular fractionation further revealed that these changes were present in both nuclear and cytoplasmic compartments (Figure 3B; Figure S3B). Since acetyl‐CoA is primarily derived from the end product of glycolysis, pyruvate, and fatty acid β‐oxidation [14], and our data showed no significant changes in either pathway in OCTN2‐overexpressing MASH‐HCC cells (Figure 2H), we reasonably speculate that the reduction in acetyl‐CoA levels is mainly due to its consumption through the acetyl group buffering function with L‐carnitine. Intracellular acetyl‐CoA is not only a key intermediate in fatty acid oxidation, glycolysis, and amino acid metabolism but also serves as the primary acetyl group donor for acetylation modifications, playing a crucial role in essential epigenetic and protein modification processes [14, 15]. We thus further examined the changes in pan‐acetylation levels. Western blot analysis revealed that pan‐acetylation levels were significantly decreased in OCTN2‐overexpressing cells, whereas OCTN2 knockdown led to a marked increase in pan‐acetylation (Figure 3C; Figure S3C). These findings suggest that OCTN2‐mediated increases in L‐carnitine promote acetyl‐CoA utilization, thereby reducing acetylation of key regulatory proteins and facilitating tumor progression.

We next performed acetylome profiling in control and OCTN2‐knockdown MASH‐HCC cells and identified a significant increase in the acetylation of p53 (Figure 3D; Figure S3D). Given that acetylation is a key post‐translational modification that primarily affects protein stability and function, we further conducted proteomic analysis in control and OCTN2‐knockdown MASH‐HCC cells. Volcano plot analysis revealed widespread changes in protein abundance (Figure S3E), and KEGG pathway enrichment identified significant activation of the p53 signaling pathway (Figure 3E). The p53 signaling pathway is centered on p53, a key tumor suppressor transcription factor whose function can be enhanced through acetylation, thereby promoting the transcriptional activation of downstream genes involved in cell cycle arrest, DNA repair, and apoptosis [22]. Western blot analysis showed that OCTN2 overexpression markedly decreased p53 acetylation, whereas OCTN2 knockdown increased it, while total p53 protein and other post‐translational modifications remained unchanged (Figure 3F; Figure S3F). qPCR further confirmed that p53 mRNA levels were unaffected by OCTN2 (Figure S3G). Although our MASH‐HCC model is based on HuH‐7 cells, which carry a gain‐of‐function p53 mutation, multiple studies have shown that acetylation at key lysines (K320, K373, K382) can partially restore mutant‐p53 function by enhancing its DNA binding and transcriptional activity while reducing aggregation and improving solubility [23, 24, 25, 26, 27, 28, 29]. Guided by these findings, we assessed the acetylation status of these lysine sites and found that OCTN2 overexpression reduced acetylation at K320, K373, and K382, whereas OCTN2 knockdown enhanced acetylation at these sites (Figure 3G; Figure S3H). Consistently, detergent‐fractionation assays showed that OCTN2 overexpression reduced the soluble fraction of p53 and increased its insoluble fraction, whereas OCTN2 knockdown increased soluble p53 and decreased its insoluble component (Figure S3I,J). Luciferase reporter assays further demonstrated that OCTN2 significantly suppressed, while its knockdown enhanced, the transcriptional activity of p53 target genes, including P21 and GADD45A (cell cycle arrest), and BAX and PUMA (apoptosis) (Figure 3H; Figure S3K). Western blotting confirmed reduced protein expression of these downstream targets in OCTN2‐overexpressing cells and increased expression upon OCTN2 knockdown (Figure 3I; Figure S3L). Moreover, EdU assays revealed that OCTN2 knockdown significantly reduced the proportion of MASH‐HCC cells in S phase (Figure 3J).

To determine whether OCTN2 exerts its effects directly through depletion of acetyl‐CoA, we supplemented OCTN2‐overexpressing MASH‐HCC cells with sodium acetate (5 mM), an acetyl‐CoA precursor. Acetate supplementation restored the OCTN2‐induced reduction in p53 acetylation and reinstated the expression of canonical p53 target genes (PUMA, BAX, GADD45A, and p21). Moreover, EdU assays showed that acetate treatment reversed the proliferative advantage conferred by OCTN2 overexpression (Figure 3K–M). Collectively, these results indicate that OCTN2 suppresses p53 signaling in MASH‐HCC by modulating acetyl‐CoA availability and protein acetylation.

To further evaluate this mechanism in vivo, we generated a hepatocyte‐specific *Octn2*‐overexpressing MASH‐HCC model using AAV8‐TBG‐*Octn2* (Figure 3N). No significant differences in body, liver, or spleen weight were observed between the AAV8‐TBG‐Ctrl and AAV8‐TBG‐*Octn2* groups (Figure S3M–O). Importantly, *Octn2* overexpression significantly increased both the number and the maximum diameter of tumor nodules (Figure 3O–Q). Consistent with the increased tumor burden, immunohistochemistry of Ki67 staining revealed enhanced tumor cell proliferation (Figure 3R). However, upon treatment with the p53 inhibitor Pifithrin‐α (PFT‐α), tumor burden was markedly increased in both control and *Octn2*‐overexpressing groups, and the previously significant difference between them was completely abolished (Figure 3O–R). Consistently, qPCR and Western blot analyses showed that *Octn2* overexpression led to decreased expression of several canonical p53 downstream targets at both the mRNA and protein levels. Notably, this *Octn2*‐induced suppression was abolished following p53 inhibition, as no significant differences were observed between the ctrl and *Octn2* groups under PFT‐α treatment, suggesting that the tumor‐promoting effect of OCTN2 relies on functional p53 activity (Figure 3S,T).

Taken together, these findings demonstrate that OCTN2 promotes MASH‐HCC progression by elevating L‐carnitine levels, reducing nuclear acetyl‐CoA availability, inhibiting p53 acetylation, and consequently impairing p53‐mediated tumor‐suppressive functions.

### 2.4 OCTN2 Suppresses the MHC‐I Pathway and Contributes to Immunotherapy Resistance in MASH‐HCC

**FIGURE 4:**
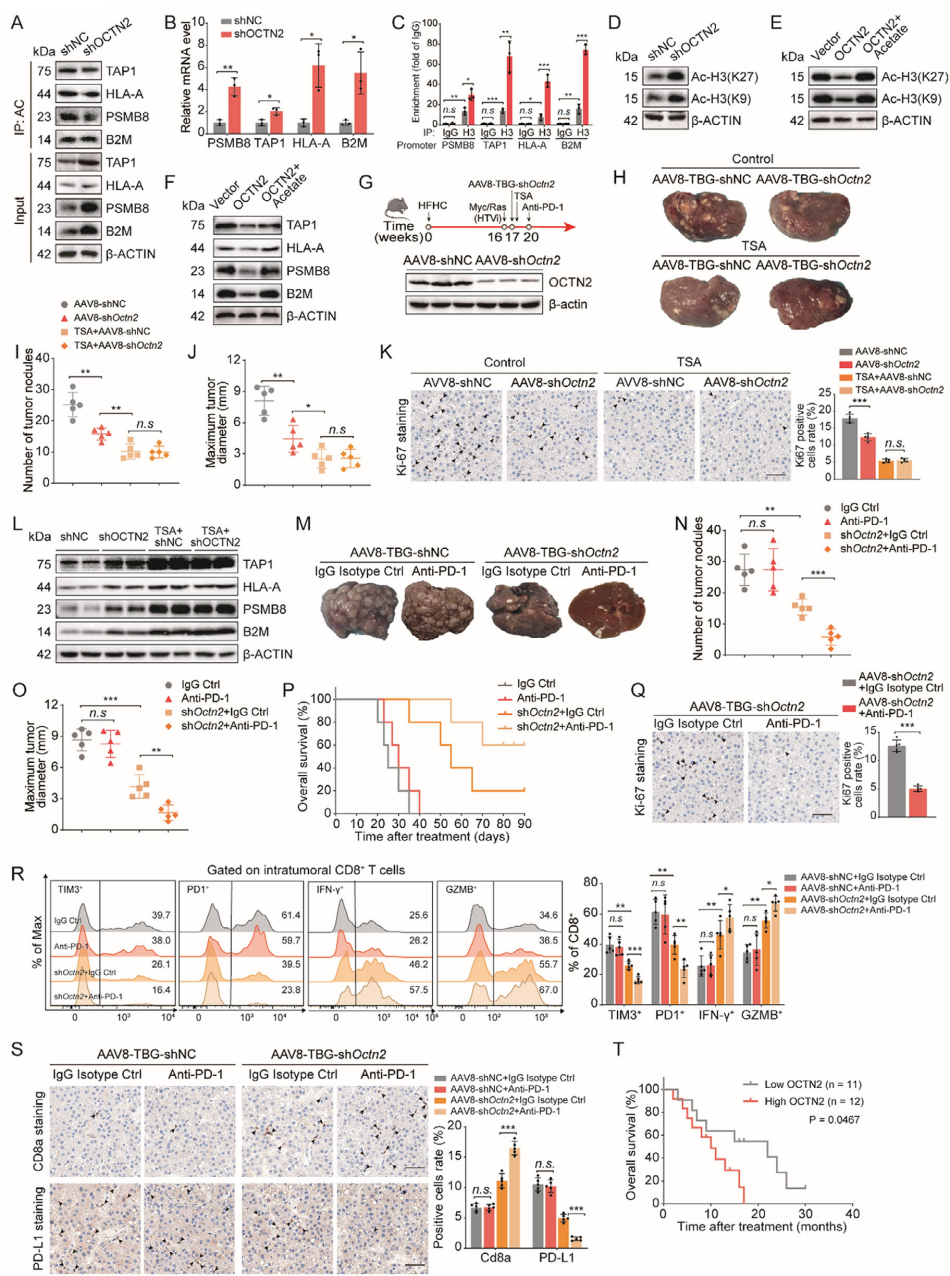
OCTN2 Suppresses the MHC‐I Pathway and Contributes to Immunotherapy Resistance in MASH‐HCC. (A) Western blot analysis of MHC‐I pathway–related proteins (PSMB8, TAP1, HLA‐A, B2M) and their acetylation status in MASH‐HCC cells transfected with shNC or shOCTN2 plasmids (n = 3 per group). (B) qRT‐PCR analysis of MHC‐I pathway gene expression in MASH‐HCC cells transfected with shNC or shOCTN2 plasmids (n = 3 per group). (C) ChIP‐qPCR analysis of H3 occupancy at the promoters of MHC‐I pathway genes in MASH‐HCC cells transfected with shNC or shOCTN2 plasmids (n = 3 per group). (D) Western blot analysis of H3K9ac and H3K27ac levels in MASH‐HCC cells transfected with shNC or shOCTN2 plasmids (n = 3 per group). (E) Western blot analysis of H3K9ac and H3K27ac in MASH‐HCC cells transfected with vector, OCTN2 plasmid, or OCTN2 plasmid plus sodium acetate (5 mM) (n = 3 per group). (F) Western blot analysis of MHC‐I pathway proteins in MASH‐HCC cells under the same conditions as in E (n = 3 per group). (G) Schematic of the hepatocyte‐specific *Octn2*‐knockdown MASH‐HCC mouse model and TSA or anti–PD‐1 treatment regimen. (H) Representative liver tumor images from mice treated with AAV8‐TBG‐shNC, AAV8‐TBG‐sh*Octn2*, TSA + AAV8‐TBG‐shNC, or TSA + AAV8‐TBG‐sh*Octn2* (n = 5 per group). (I,J) Quantification of tumor nodule number and maximum tumor diameter in the groups shown in H (n = 5 per group). (K) Representative immunohistochemistry images of Ki‐67 staining, accompanied by corresponding quantitative analysis, showing tumor proliferation in the groups shown in H (n = 5 per group). Scale bars, 50 µm. (L) Western blot analysis of MHC‐I pathway proteins in liver tumors from the groups shown in H (n = 5 per group). (M) Representative liver tumor images from mice treated with AAV8‐TBG ‐shNC + IgG isotype, AAV8‐TBG ‐shNC + anti–PD‐1, AAV8‐TBG ‐sh*Octn2* + IgG, or AAV8‐TBG ‐sh*Octn2* + anti–PD‐1 (n = 5 per group). (N,O) Quantification of tumor nodule number and maximum tumor diameter in the groups shown in M (n = 5 per group). (P) Kaplan–Meier survival curves for mice in the treatment groups are shown in M (n = 5 per group). (Q) Ki‐67 immunohistochemistry, together with corresponding quantitative analysis, showing tumor proliferation in the AAV8‐TBG ‐sh*Octn2* + IgG and AAV8‐TBG ‐sh*Octn2* + anti–PD‐1 groups (n = 5 per group). Scale bars, 50 µm. (R) Flow cytometric analysis of tumor‐infiltrating CD8^+^ T cells expressing TIM3, PD1, IFN‐γ, or GZMB in the groups shown in M (n = 5 per group). (S) CD8a and PD‐L1 immunohistochemistry with quantification, showing CD8^+^ T‐cell infiltration and immune suppression status in the groups shown in M (n = 5 per group). Scale bars, 50 µm. (T) Kaplan–Meier survival curves for MASH‐HCC patients receiving anti–PD‐1 therapy in the low‐OCTN2–expression group (n = 11) and the high‐OCTN2–expression group (n = 12). The data are expressed as the mean ± SD. *p*‐values were determined by two‐tailed Student's *t*‐test (B, C) or one‐way ANOVA followed by a post hoc Tukey test (I, J, K, N, O, R, S). Statistical significance: n.s. means not significant, ^*^ *p* < 0.05, ^**^ *p* < 0.01, ^***^ *p* < 0.001.

KEGG pathway analysis of the proteomic data revealed not only activation of the p53 signaling pathway but also a significant upregulation of the antigen processing and presentation pathway in MASH‐HCC cells following OCTN2 knockdown (Figure 3E; Figure S3E). Consistently, qPCR and Western blot analyses showed that key MHC‐I components—including PSMB8, TAP1, HLA‐A, and B2M—were suppressed by OCTN2 overexpression and elevated by OCTN2 knockdown, whereas the acetylation status of these proteins remained unchanged (Figure 4A,B; Figure S4A,B). These findings suggested that OCTN2 may regulates MHC‐I pathway activity primarily at the transcriptional level. We previously demonstrated that OCTN2 markedly reduces protein pan‐acetylation level in MASH‐HCC cells (Figure 3C; Figure S3C). Given that histones account for the majority of intracellular acetylation events [30], this observation led us to hypothesize that OCTN2 may repress MHC‐I transcription through histone deacetylation. Supporting this hypothesis, prior studies have established that acetylation of histone H3—particularly at K9 and K27 (H3K9ac and H3K27ac)—promotes the transcription of MHC‐I–related genes [31, 32, 33]. In line with this mechanistic framework, our acetylome profiling revealed that OCTN2 knockdown produced the most pronounced increase in histone H3 acetylation (Figure 3D; Figure S3D). To directly test this mechanism, ChIP assays showed that OCTN2 overexpression reduced the enrichment of MHC‐I pathway gene promoters at H3, whereas OCTN2 knockdown increased the enrichment of these promoters (Figure 4C; Figure S4C). Western blotting further confirmed that OCTN2 overexpression decreased H3K9ac and H3K27ac levels, while OCTN2 knockdown led to increased acetylation at these sites (Figure 4D; Figure S4D). Moreover, restoring acetyl‐CoA availability in OCTN2‐overexpressing cells via sodium acetate supplementation rescued H3K9ac and H3K27ac levels and reactivated MHC‐I gene expression (Figure 4E,F). Furthermore, treatment with the histone deacetylase inhibitor trichostatin A (TSA) abolished the differences in MHC‐I gene expression between OCTN2 knockdown and control cells (Figure S4E,F), demonstrating that histone acetylation underlies OCTN2‐mediated transcriptional repression of the MHC‐I pathway.

To evaluate this mechanism in vivo, we established a hepatocyte‐specific *Octn2*‐knockdown MASH‐HCC mouse model using AAV8‐TBG‐sh*Octn2* (Figure 4G). Body, liver, and spleen weights were comparable between AAV8‐TBG‐shNC and AAV8‐TBG‐sh*Octn2* mice (Figure S4G–I). *Octn2* knockdown significantly reduced both the number and maximum diameter of liver tumor nodules (Figure 4H–J). Ki67 immunohistochemistry revealed decreased tumor cell proliferation in *Octn2*‐knockdown tumors (Figure 4K). However, treatment with TSA eliminated these differences, resulting in comparable tumor burden and proliferative activity between the two groups (Figure 4H–K). Consistently, qRT‐PCR and Western blot analyses showed that *Octn2* knockdown increased MHC‐I gene expression at both the mRNA and protein levels, whereas the difference between the two groups was completely abolished following TSA administration (Figure 4L; Figure S4J). Collectively, these findings demonstrate that OCTN2 suppresses MHC‐I pathway activation in MASH‐HCC by reducing histone H3 acetylation, thereby promoting tumor progression.

Since MHC‐I pathway activity directly affects CD8^+^ T cell‐mediated tumor recognition and clearance [34], and MASH‐HCC has been reported to exhibit resistance to immune checkpoint inhibitors (ICIs), such as anti‐PD‐1/PD‐L1 antibodies [5], we hypothesized that OCTN2 knockdown might enhance the efficacy of anti‐PD‐1 immunotherapy by restoring MHC‐I expression. To isolate this mechanism from p53‐related effects, we inhibited p53 expression in MASH‐HCC cells. Under these conditions, OCTN2 knockdown had no effect on intrinsic tumor cell proliferation in colony formation or CCK‐8 assays. However, when co‐cultured with CD8^+^ T cells, OCTN2 knockdown significantly enhanced CD8^+^ T cell–mediated cytotoxicity against MASH‐HCC cells (Figure S4L,M). These findings suggest that OCTN2 promotes MASH‐HCC progression not only via the p53 signaling pathway but also by impairing antigen presentation and reducing CD8^+^ T cell–mediated immune surveillance. We next tested this mechanism in vivo by treating AAV8‐TBG‐shNC and AAV8‐TBG‐sh*Octn2* MASH‐HCC mice with either an IgG control or an anti‐PD‐1 antibody. No significant differences in body, liver, or spleen weight were observed across the four groups (Figure S4M–O). Consistent with previous reports, MASH‐HCC mice showed poor response to anti‐PD‐1 monotherapy [5]; however, combining AAV8‐sh*Octn2* with anti‐PD‐1 significantly improved therapeutic efficacy (Figure 4M). This combination markedly reduced tumor nodule number and maximal tumor diameter and substantially extended overall survival (Figure 4N–P). Ki67 staining further confirmed that sh*Octn2* synergized with anti‐PD‐1 to reduce tumor proliferation (Figure 4Q). We next examined remodeling of the tumor immune microenvironment. Flow cytometric analysis demonstrated that anti–PD‐1 monotherapy caused minimal changes in T‐cell exhaustion or effector function, whereas the combination of AAV8‐sh*Octn2* and anti–PD‐1 significantly reduced TIM3^+^CD8^+^ and PD1^+^CD8^+^ exhausted T‐cell subsets and increased IFNγ^+^CD8^+^ and GZMB^+^CD8^+^ effector populations (Figure 4R; Figure S4P). Immunohistochemistry for CD8a and PD‐L1 further supported these findings: anti–PD‐1 alone had little impact on immune infiltration or PD‐L1 expression, whereas the combination treatment markedly enhanced intratumoral CD8^+^ T‐cell infiltration and reduced PD‐L1 levels (Figure 4S). Finally, we retrospectively analyzed clinical data from 23 MASH‐HCC patients treated with anti–PD‐1 therapy. Stratification based on OCTN2 expression revealed that patients with low OCTN2 expression had significantly longer overall survival than those with high OCTN2 expression (Figure 4T), underscoring the clinical relevance of OCTN2 as a determinant of immunotherapy responsiveness in MASH‐HCC.

Collectively, these findings demonstrate that OCTN2 suppresses the MHC‐I antigen presentation pathway by reducing histone H3 acetylation, thereby promoting immune evasion in MASH‐HCC. Conversely, OCTN2 knockdown restores MHC‐I expression, reactivates an immunologically responsive TME, and markedly enhances the therapeutic efficacy of anti–PD‐1 immunotherapy.

### 2.5 DZIP3 Promotes OCTN2 Degradation via K48‐Linked Polyubiquitination

**FIGURE 5:**
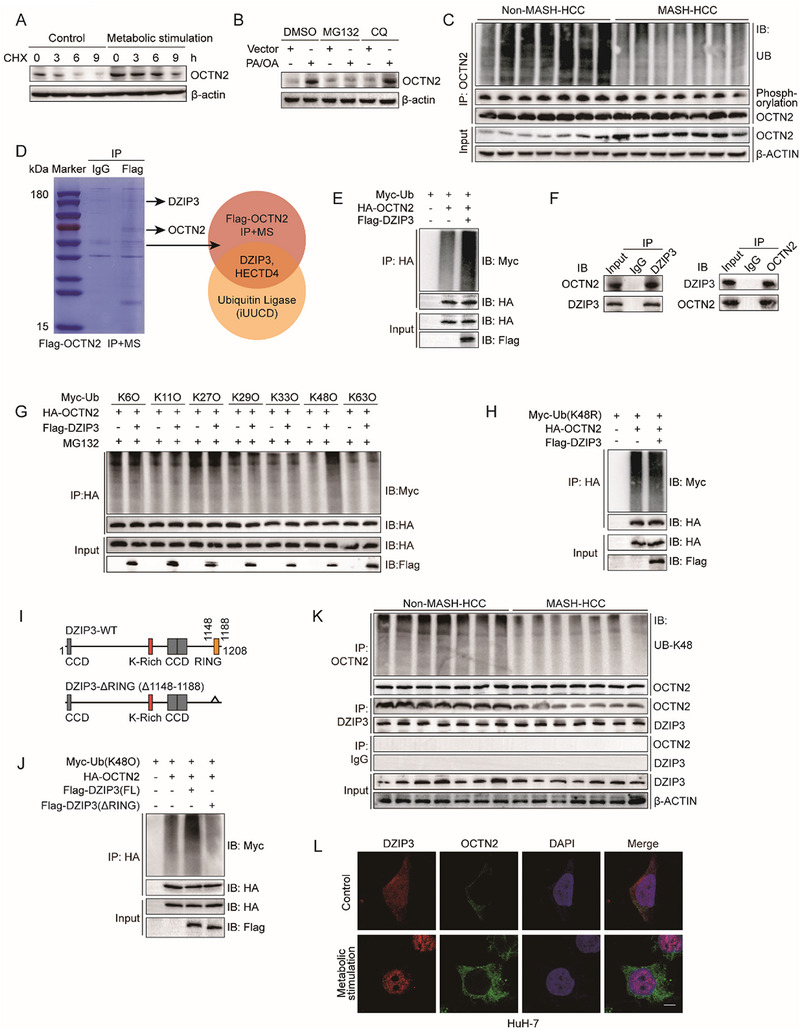
DZIP3 Promotes OCTN2 Degradation via K48‐Linked Polyubiquitination. (A) Western blot analysis of OCTN2 protein expression in HuH‐7 cells treated with control (BSA) or metabolic stimulation (PA/OA and IL‐6/TNF‐α/LPS), followed by cycloheximide (CHX, 100 µg/mL) treatment for the indicated time points (n = 3 per group). (B) Western blot analysis of OCTN2 protein levels in HuH‐7 cells treated with BSA or PA/OA, followed by treatment with either 25 µM DMSO, MG132, or chloroquine (CQ) for 6 h before cell harvesting (n = 3 per group). (C) Western blot analysis showing OCTN2 protein, phosphorylation, and ubiquitination levels in clinical tumor tissues from MASH‐HCC and non‐MASH‐HCC patients (n = 7 per group). (D) Venn diagram showing the intersection between OCTN2‐binding proteins identified by IP/MS and ubiquitination‐related proteins from the iUUCD database, highlighting DZIP3 and HECTD4 as candidates. (E) Western blot analysis showing OCTN2 ubiquitination levels in MASH‐HCC cells co‐transfected with Flag‐tagged DZIP3 (n = 3 per group). (F) Endogenous co‐IP analysis confirming the interaction between DZIP3 and OCTN2 in MASH‐HCC cells (n = 3 per group). (G) Western blot analysis of OCTN2 ubiquitination in MASH‐HCC cells co‐transfected with HA‐tagged OCTN2, Flag‐tagged DZIP3, and Myc‐tagged ubiquitin variants (K6O, K11O, K27O, K29O, K33O, K48O, and K63O) (n = 3 per group). (H) Western blot analysis of OCTN2 ubiquitination in MASH‐HCC cells co‐transfected with HA‐OCTN2, Flag‐DZIP3, and a K48R‐mutant Myc‐ubiquitin plasmid (n = 3 per group). (I) Schematic diagram illustrating the construction of DZIP3 with RING domain deletion (ΔRING). (J) Western blot analysis of K48‐linked ubiquitination of OCTN2 in MASH‐HCC cells co‐transfected with either Flag‐ΔRING DZIP3 or full‐length DZIP3 plasmids (n = 3 per group). (K) Western blot analysis showing DZIP3‐OCTN2 interaction and OCTN2 K48‐linked polyubiquitination in clinical tumor tissues from MASH‐HCC and non‐MASH‐HCC patients (n = 7 per group). (L) Immunofluorescence analysis of subcellular localization and fluorescence intensity of DZIP3 and OCTN2 in HuH‐7 cells treated with control (BSA) or metabolic stimulation (PA/OA and IL‐6/TNF‐α/LPS) (n = 3 per group). Scale bars, 25 µm).

Compared to non‐MASH‐HCC, the fold increase in OCTN2 protein levels in MASH‐HCC was substantially greater than that of its mRNA expression (Figure 1F,G; Figure S1F,G), suggesting that the elevated OCTN2 expression in MASH‐HCC may not be solely attributed to transcriptional regulation, but is likely also influenced by post‐transcriptional or protein‐level regulatory mechanisms. To investigate this, we treated MASH‐HCC cells with cycloheximide (CHX) to inhibit protein synthesis. The results showed that OCTN2 protein was markedly more stable in MASH‐HCC cells than in non‐MASH‐HCC cells (Figure 5A), indicating a role for post‐translational regulation. Furthermore, treatment with the proteasome inhibitor MG132–but not the lysosomal inhibitor chloroquine (CQ)–restored OCTN2 protein levels (Figure 5B), suggesting that OCTN2 degradation is proteasome‐dependent and suppressed in MASH‐HCC. Immunoprecipitation (IP) of clinical tumor samples confirmed that OCTN2 ubiquitination was significantly reduced in MASH‐HCC, whereas phosphorylation remained unchanged (Figure 5C). These findings indicate that impaired ubiquitination may underlie the elevated OCTN2 protein levels in MASH‐HCC.

Subsequently, we overexpressed Flag‐tagged OCTN2 in MASH‐HCC cells and performed immunoprecipitation followed by mass spectrometry (IP/MS). By intersecting the identified proteins with ubiquitination‐related proteins from the iUUCD database, we identified two potential E3 ubiquitin ligases: DZIP3 and HECTD4 (Figure 5D). However, overexpression of HECTD4 failed to alter the ubiquitination level of OCTN2 (Figure S5A), whereas overexpression of Flag‐tagged DZIP3 significantly increased OCTN2 ubiquitination (Figure 5E). In addition, co‐immunoprecipitation using both endogenous and exogenous systems further confirmed the interaction between DZIP3 and OCTN2 (Figure 5F, Figure S5B). Moreover, Ubiquitination profiling showed that DZIP3 primarily promoted K48‐linked polyubiquitination of OCTN2, while the effect was abolished when ubiquitin was mutated at lysine 48 (K48R) (Figure 5G,H). DZIP3 contains an RNA‐binding motif called lysine (K)‐rich region (or KR motif), a RING domain, and several coiled–coiled domains (CCD; Figure S5C). As reported, the RING domain of DZIP3 is essential for its E3 ligase activity [35]. Consistently, the RING domain–deficient mutant of DZIP3 (Flag‐ΔRING DZIP3) failed to promote OCTN2 K48‐linked ubiquitination compared to full‐length DZIP3 (Figure 5I,J). Taken together, these results demonstrate that DZIP3 facilitates OCTN2 degradation through K48‐linked polyubiquitination mediated by its RING domain.

Interestingly, analysis of clinical samples revealed no significant difference in DZIP3 expression between MASH‐HCC and non‐MASH‐HCC tissues. However, the interaction between DZIP3 and OCTN2 was markedly reduced in MASH‐HCC, accompanied by diminished K48‐linked ubiquitination of OCTN2 (Figure 5K). Immunofluorescence staining showed that under normal conditions, DZIP3 is distributed in both the cytoplasm and nucleus. Upon metabolic stimulation, however, DZIP3 predominantly localized to the nucleus. Notably, OCTN2 remained predominantly localized to the cytoplasm and plasma membrane under both conditions; however, its fluorescence intensity was markedly increased in MASH‐HCC cells, consistent with enhanced protein accumulation (Figure 5L). These results suggest that in MASH‐HCC, DZIP3 is sequestered in the nucleus, reducing its interaction with cytoplasmic OCTN2 and thereby limiting K48‐linked ubiquitination‐mediated degradation of OCTN2. This subcellular relocalization of DZIP3 likely contributes to the accumulation of OCTN2 protein in MASH‐HCC.

### 2.6 LINCMD1 Regulates OCTN2 Ubiquitin‐Mediated Degradation via DZIP3 in MASH‐HCC

**FIGURE 6:**
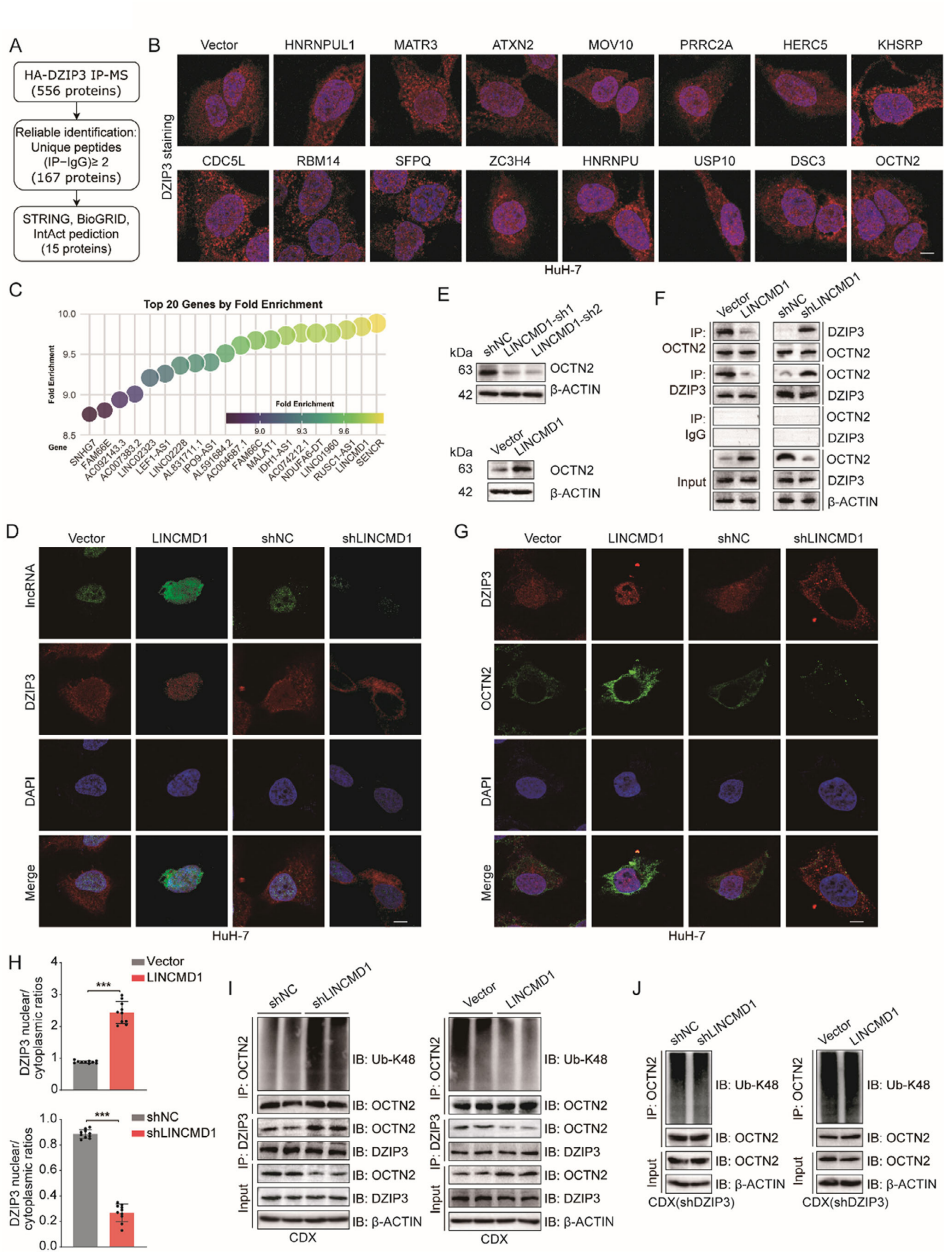
LINCMD1 Regulates OCTN2 Ubiquitin‐Mediated Degradation via DZIP3 in MASH‐HCC. (A) Workflow for identifying potential DZIP3‐interacting proteins using IP/MS combined with bioinformatics analysis. (B) Immunofluorescence staining of DZIP3 following transfection of plasmids encoding candidate interacting proteins (n = 3 per group). (C) RIP assay showing the top 20 lncRNAs enriched by DZIP3 in MASH‐HCC cells. (D) Combined FISH and immunofluorescence staining showing subcellular localization and fluorescence intensity of LINCMD1 and DZIP3 in MASH‐HCC cells after LINCMD1 overexpression or knockdown (n = 3 per group). Scale bars, 25 µm. (E) Western blot analysis of OCTN2 protein levels in MASH‐HCC cells following LINCMD1 knockdown or overexpression (n = 3 per group). (F) Co‐IP analysis showing the interaction between DZIP3 and OCTN2 in MASH‐HCC cells following LINCMD1 overexpression or knockdown (n = 3 per group). (G) Immunofluorescence analysis showing subcellular localization and fluorescence intensity of DZIP3 and OCTN2 in MASH‐HCC cells after LINCMD1 overexpression or knockdown (n = 10 per group). Scale bars, 25 µm. (H) Quantification of the nuclear‐to‐cytoplasmic ratio of DZIP3 based on immunofluorescence analysis in MASH‐HCC cells following LINCMD1 overexpression or knockdown (n = 10 per group). (I) Western blot analysis showing the interaction between DZIP3 and OCTN2, as well as K48‐linked polyubiquitination levels of OCTN2, in HFHC‐fed CDX tumors following LINCMD1 knockdown or overexpression (n = 3 per group). (J) Western blot analysis showing changes in K48‐linked polyubiquitination of OCTN2 in HFHC‐fed CDX tumors derived from DZIP3‐knockdown HuH‐7 cells after LINCMD1 overexpression or knockdown (n = 3 per group). The data are expressed as the mean ± SD. *p*‐values were determined by a two‐tailed Student's *t*‐test (H). Statistical significance: n.s. means not significant, ^*^ *p* < 0.05, ^**^ *p* < 0.01, ^***^ *p* < 0.001.

To investigate the mechanism underlying the altered subcellular localization of DZIP3 in non‐MASH‐HCC vs. MASH‐HCC, we overexpressed HA‐tagged DZIP3 in MASH‐HCC cells and performed IP/MS to identify potential interacting proteins (Figure 6A). However, immunofluorescence analysis showed that these candidate proteins failed to alter the intracellular localization of DZIP3 (Figure 6B). Given that DZIP3 is a poorly characterized RNA‐binding RING‐H2 E3 ubiquitin ligase [35], and that among various types of RNAs, lncRNAs can act as molecular scaffolds or guides to alter protein localization through direct binding [36], we hypothesized that lncRNAs might be involved in regulating the subcellular localization of DZIP3 in MASH‐HCC. To explore this possibility, we performed RIP‐seq analysis and identified numerous lncRNAs interacting with DZIP3. These lncRNAs were ranked based on the extent of their binding enrichment with DZIP3 (Figure 6C). Among the top five candidates, only one specific lncRNA (LINCMD1) was able to alter the intracellular localization of DZIP3, while the others had no effect (Figure 6D; Figure S6A). Combined FISH and immunofluorescence staining demonstrated that lncRNA LINCMD1 was predominantly localized in the nucleus. Overexpression of LINCMD1 promoted nuclear accumulation of DZIP3, whereas its knockdown redirected DZIP3 to the cytoplasm (Figure 6D). In addition, Western blot analysis confirmed that modulating LINCMD1 expression did not alter either the protein abundance or phosphorylation level of DZIP3 (Figure S6B–D). Direct binding between LINCMD1 and DZIP3 was validated by RIP and RNA pull‐down assays (Figure S6E,F). Together, these data indicate that LINCMD1 regulates the subcellular localization of DZIP3.

We next examined whether LINCMD1 regulates OCTN2 expression via DZIP3. As DZIP3 primarily functions as an E3 ubiquitin ligase [35], we first assessed its ubiquitination regulatory spectrum. Western blot analysis of ubiquitination assays revealed that DZIP3 ubiquitinates ATXN2, MOV10, KHSRP, SFPQ, HNRNPU, and OCTN2, but not other interacting candidates (Figure S7A). Upon LINCMD1 overexpression, the ubiquitination of ATXN2, MOV10, and OCTN2 decreased, whereas the ubiquitination of KHSRP and SFPQ increased (Figure S7B). To determine the functional relevance of these substrates in MASH‐HCC, we performed colony formation assays. Notably, among the five DZIP3 substrates modulated by LINCMD1, only OCTN2 significantly enhanced the proliferative capacity of MASH‐HCC cells (Figure S7C). This identifies OCTN2 as the primary functional effector in this context. Accordingly, subsequent analyses focused on dissecting the LINCMD1–DZIP3–OCTN2 regulatory axis. Ubiquitination site mapping further confirmed that LINCMD1 reduces K48‐linked polyubiquitination of OCTN2 (Figure S7D,E). Consistently, overexpression of LINCMD1 increased OCTN2 protein levels and intracellular L‐carnitine content, whereas knockdown of LINCMD1 produced the opposite effects (Figure 6E; Figure S7F,G). Co‐IP studies further revealed that LINCMD1 overexpression diminished the interaction between DZIP3 and OCTN2, while LINCMD1 knockdown enhanced this interaction (Figure 6F). Immunofluorescence analysis and quantitative assessment showed that LINCMD1 overexpression promoted nuclear localization of DZIP3 and elevated cytoplasmic OCTN2 levels, whereas LINCMD1 knockdown resulted in cytoplasmic redistribution of DZIP3 and reduced OCTN2 expression (Figure 6G,H). To validate these findings in vivo, we employed HFHC‐fed cell‐derived xenograft (CDX) models. In these models, LINCMD1 silencing enhanced DZIP3–OCTN2 interaction and increased K48‐linked OCTN2 ubiquitination, whereas LINCMD1 overexpression reversed these changes (Figure 6I). Importantly, DZIP3 knockdown abolished the effects of LINCMD1 overexpression or depletion on OCTN2 expression and ubiquitination in vivo (Figure 6J; Figure S7H).

Taken together, our results indicate that in MASH‐HCC, LINCMD1 binds to DZIP3 and promotes its nuclear localization, thereby competitively reducing its interaction with OCTN2. This in turn inhibits DZIP3‐mediated K48‐linked ubiquitination and proteasomal degradation of OCTN2, leading to increased OCTN2 protein stability and enhanced L‐carnitine uptake.

### 2.7 LINCMD1 Promotes MASH‐HCC Progression by Regulating the Carnitine‐Acetyl Group Buffering System via OCTN2

**FIGURE 7:**
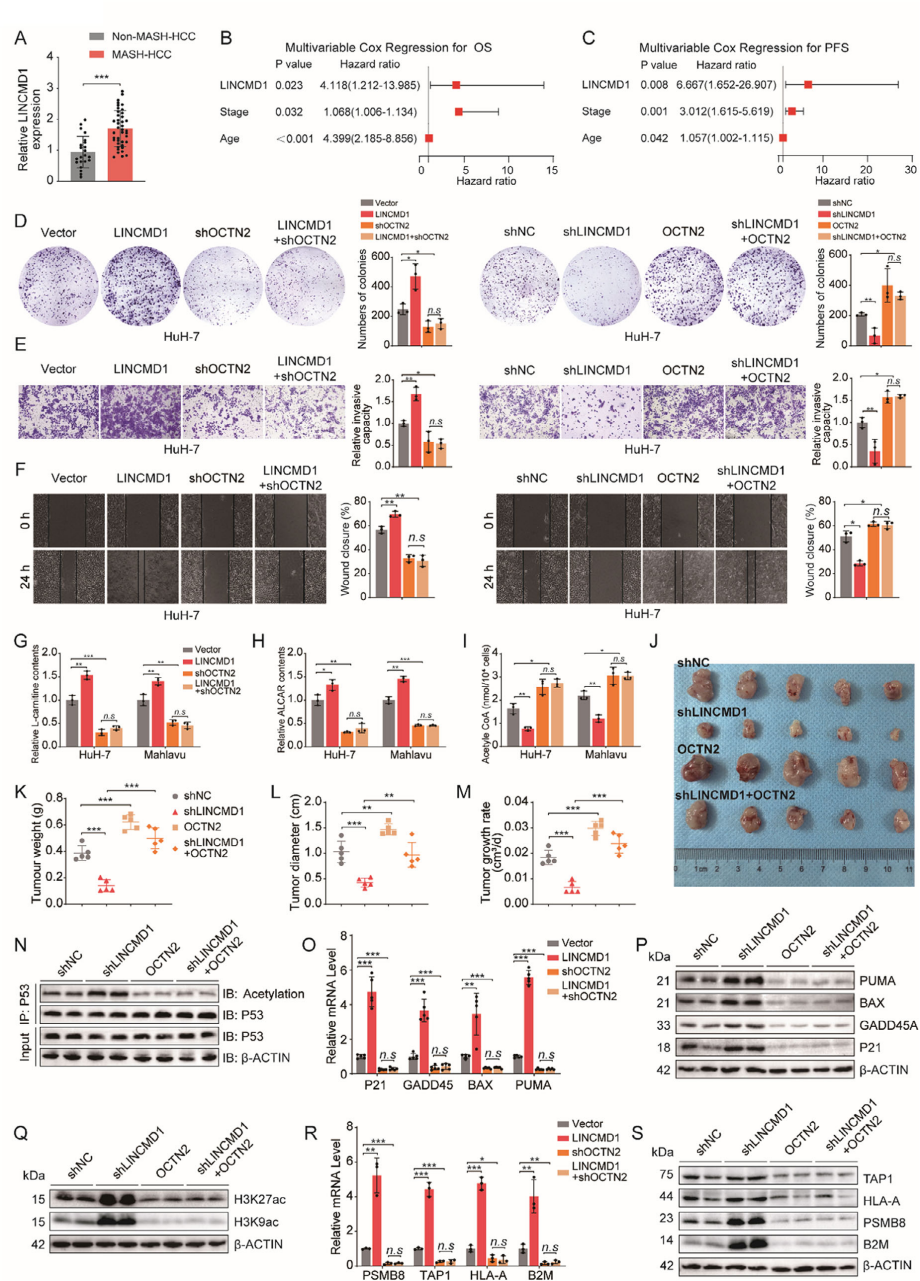
LINCMD1 promotes MASH‐HCC progression through OCTN2. (A) qRT‐PCR analysis of LINCMD1 expression in clinical tumor samples from non‐MASH‐HCC (n = 22) and MASH‐HCC (n = 41) patients. (B,C) Multivariate Cox regression analyses evaluating LINCMD1 expression, tumor stage, and age with overall survival (OS) (B) and progression‐free survival (PFS) (C) in the MASH‐HCC cohort (n = 41). (D) Colony formation assay showing the proliferative capacity of MASH‐HCC cells transfected with vector, LINCMD1, shOCTN2, or LINCMD1 + shOCTN2 plasmids, and with shNC, shLINCMD1, OCTN2, or shLINCMD1 + OCTN2 (n = 3 per group). (E) Transwell invasion assay showing the invasive capacity of MASH‐HCC cells under the same treatments as in D (n = 3 per group). (G–I) Quantification of intracellular levels of L‐carnitine, ALCAR, and acetyl‐CoA in MASH‐HCC cells (HuH‐7 and Mahlavu) transfected with vector, LINCMD1, shOCTN2, or LINCMD1 + shOCTN2 (n = 3 per group). (J) Representative images of subcutaneous CDX tumors established in HFHC‐fed mice using HuH‐7 cells transfected with shNC, shLINCMD1, OCTN2, or shLINCMD1 + OCTN2 plasmids (n = 5 per group). (K–M) Quantification of tumor weights, maximum tumor diameters, and tumor growth rates in CDX models is shown in J (n = 5 per group). (N) Western blot analysis showing p53 acetylation levels in CDX tumors from the groups in J (n = 5 per group). (O,P) qRT‐PCR and western blot analyses showing mRNA and protein expression of canonical p53 downstream target genes (P21, GADD45A, BAX, PUMA) in CDX tumors from the groups in J (n = 5 per group). (Q) Western blot analysis showing histone H3 acetylation levels in CDX tumors from the groups in J (n = 5 per group). (R,S) qRT‐PCR and western blot analyses showing mRNA and protein expression of MHC‐I pathway genes (PSMB8, TAP1, HLA‐A, B2M) in CDX tumors from the groups in J (n = 5 per group). The data are expressed as the mean ± SD. *p*‐values were determined by two‐tailed Student's *t*‐test (A) or one‐way ANOVA followed by a post hoc Tukey test (D, E, F, G, H, I, K, L, M, O, R). Statistical significance: n.s. means not significant, ^*^ *p* < 0.05, ^**^ *p* < 0.01, ^***^ *p* < 0.001.

To further elucidate the functional role of LINCMD1 in MASH‐HCC progression, we first examined its expression in clinical samples and cell lines. qPCR analysis showed that LINCMD1 was markedly upregulated in MASH‐HCC tumor tissues and cell lines compared with non‐MASH‐HCC counterparts (Figure 7A; Figure S8A,B). In our 41‐patient MASH‐HCC cohort, univariate and multivariate Cox regression analyses further demonstrated that LINCMD1 is an independent prognostic predictor for both OS and PFS (Tables S4 and S5; Figure 7B,C). To define the functional contribution of LINCMD1 to MASH‐HCC progression, we next conducted a series of in vitro and in vivo assays. These experiments consistently demonstrated that LINCMD1 promotes MASH‐HCC cell proliferation, migration, and invasion (Figure S8C–H).

To determine whether OCTN2 mediates these oncogenic effects, we performed rescue experiments. Knockdown of OCTN2 abolished the pro‐tumorigenic effects of LINCMD1 overexpression, whereas reintroduction of OCTN2 restored the malignant phenotypes in LINCMD1‐silenced cells (Figure 7D–F). In line with this, the L‐carnitine and ALCAR increases induced by LINCMD1 overexpression were completely abrogated by OCTN2 knockdown. Similarly, the reduction in intracellular acetyl‐CoA levels caused by LINCMD1 was reversed upon OCTN2 depletion (Figure 7G–I). In vivo, LINCMD1 knockdown significantly impaired tumor growth in CDX models, as reflected by decreased tumor volume, diameter, and growth rate. Importantly, OCTN2 overexpression restored tumor growth in LINCMD1‐deficient tumors (Figure 7J–M). Western blot analysis of CDX tumor tissues showed that LINCMD1 knockdown increased the acetylation levels of p53 and histone H3, while OCTN2 overexpression reversed these effects (Figure 7N,Q). In parallel, knockdown of LINCMD1 upregulated both the mRNA and protein levels of canonical p53 downstream tumor suppressor genes as well as key components of the MHC‐I antigen presentation pathway—effects that were abolished upon OCTN2 overexpression (Figure 7O,P,R,S).

Taken together, these findings demonstrate that LINCMD1 promotes MASH‐HCC progression by regulating the carnitine‐acetyl group buffering system via OCTN2. Mechanistically, this axis suppresses the p53 signaling pathway, facilitating tumor growth, while concurrently downregulating MHC‐I pathway activity, thereby contributing to immune evasion in MASH‐HCC.

### 2.8 LNP‐ASO‐LINCMD1 Reduces Tumor Burden and Increases the Efficacy of anti‐PD‐1 in MASH‐HCC

**FIGURE 8:**
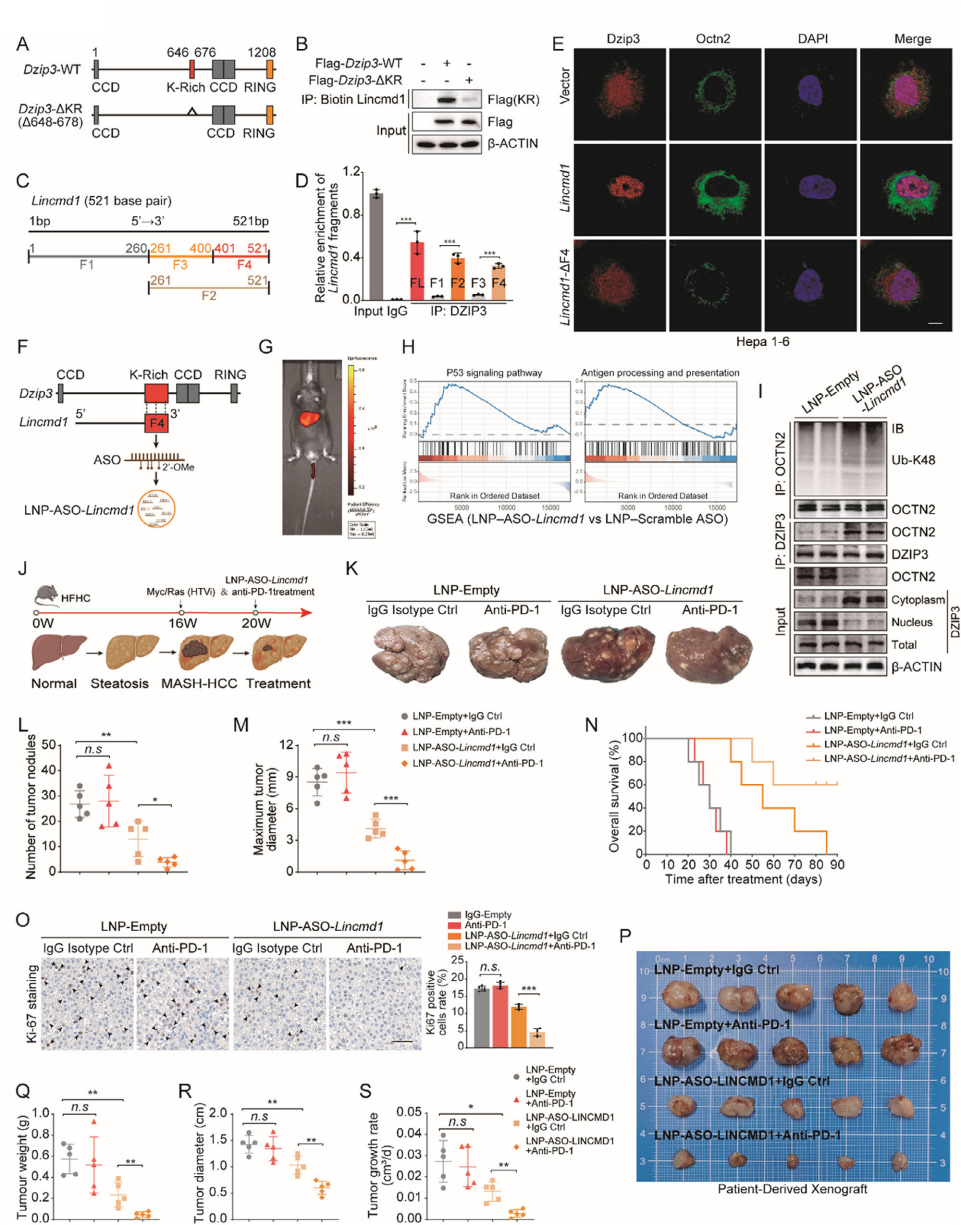
LNP‐ASO‐LINCMD1 reduces tumor burden and increases the efficacy of anti‐PD‐1 in MASH‐HCC. (A) Schematic illustration of the domain structure of *Dzip3* and construction of the KR motif–deficient mutant (*Dzip3*‐ΔKR). (B) Biotin RNA pull‐down assays showing that deletion of the KR motif impairs *Dzip3* binding to *Lincmd1* (n = 3 per group). (C) Schematic showing predicted functional regions (F1‐F4) of *Lincmd1*. (D) RIP‐qPCR showing *Dzip3* enrichment of full‐length *Lincmd1* and individual fragments (n = 3 per group). (E) Representative immunofluorescence images of *Dzip3* and *Octn2* in Hepa1‐6 cells co‐transfected with vector, *Lincmd1*, or *Lincmd1*‐ΔF4 (n = 3 per group). Scale bar, 25 µm. (F) Schematic of ASO design targeting the F4 region of *Lincmd1* with 2’‐O‐methyl (2’‐OMe) chemical modifications. (G) *In* *vivo* imaging demonstrating liver accumulation of LNP‐ASO‐*Lincmd1* following tail vein injection. (H) GSEA analysis showing activation of the p53 signaling pathway and the antigen processing and presentation pathway in MASH‐HCC tumors treated with LNP‐ASO‐*Lincmd1* compared with those treated with LNP‐Scramble‐ASO (n = 3 per group). (I) Western blot analysis assessing *Dzip3* abundance in different cellular fractions, the interaction between *Dzip3* and *Octn2*, and the K48‐linked polyubiquitination status of *Octn2* in mice treated with LNP‐Empty or LNP‐ASO‐*Lincmd1* (n = 3 per group). (J) Schematic of treatment protocol for anti‐PD‐1 monotherapy or combination with LNP‐ASO‐*Lincmd1* in MASH‐HCC mice. (K) Representative images of orthotopic liver tumors in mice treated with LNP‐Empty + IgG isotype control, LNP‐ Empty + anti–PD‐1, LNP‐ASO‐*Lincmd1* + IgG, or LNP‐ASO‐*Lincmd1* + anti‐PD‐1 (n = 5 per group). (L,M) Quantification of tumor nodule number and maximum diameter in liver tissues from the same treatment groups is shown in K. (N) Kaplan–Meier survival analysis of mice from the treatment groups in K. (O) Representative immunohistochemistry images of Ki‐67 staining, accompanied by corresponding quantitative analysis, showing tumor proliferation in the groups shown in K (n = 5 per group). Scale bars, 50 µm. (P) Representative images of subcutaneous patient‐derived xenograft (PDX) tumors established in HFHC‐fed humanized mice and treated with the same groups as in K (n = 5 per group). (Q–S) Quantification of tumor weights, maximum tumor diameters, and tumor growth rates in the PDX models is shown in P (n = 5 per group). The data are expressed as the mean ± SD. *p*‐values were determined by two‐tailed Student's *t*‐test (D) or one‐way ANOVA followed by a post hoc Tukey test (L, M, O, Q, R, S). Statistical significance: n.s. means not significant, ^*^ *p* < 0.05, ^**^ *p* < 0.01, ^***^ *p* < 0.001.

To further delineate how LINCMD1 interacts with DZIP3, we next mapped the specific binding regions between the two molecules. Previous studies have reported that DZIP3 binds RNA through a conserved KR motif (Figure S9A) [35, 37]. Consistent with this, biotin RNA pull‐down and RIP assays demonstrated that deletion of the KR motif (*Dzip3*‐ΔKR) markedly impaired its ability to bind *Lincmd1* (Figure 8A,B; Figure S9B). Next, we employed the RPISeq tool to predict potential binding sequences of *Lincmd1* to *Dzip3*. Notably, the region spanning nucleotides 401–500 bp exhibited the highest binding probability, with a prediction score of 0.60 using the Random Forest (RF) classifier and 0.81 using the Support Vector Machine (SVM) classifier (both > 0.5, indicating positive interaction). Based on these predictions, we divided the full‐length *Lincmd1* transcript into four fragments: F1 (1–260 bp), F2 (261–521 bp), F3 (261–400 bp), and F4 (401–521 bp) (Figure 8C). We then performed RIP followed by qPCR to validate these predictions. The results showed that F2 and F4 were significantly enriched by *Dzip3*, while F1 and F3 were not (Figure 8D), supporting the computational prediction. Immunofluorescence staining further showed that deletion of the F4 region abolished the ability of *Lincmd1* to alter the subcellular localization of *Dzip3* or regulate *Octn2* expression (Figure 8E). Collectively, these results demonstrate that the F4 region of *Lincmd1* directly interacts with the KR motif of *Dzip3*.

Lipid nanoparticle (LNP)‐based delivery of nucleic acid therapeutics represents a promising strategy for disease treatment [38]. Given that aberrant expression of OCTN2 is implicated in severe conditions such as primary carnitine deficiency [39], and that no pharmacological inhibitors of DZIP3 are currently available, we employed an FDA‐approved LNP formulation to deliver antisense oligonucleotides (ASOs) specifically targeting the *Dzip3*‐binding region of *Lincmd1*. Building on the identified functional binding region of *Lincmd1*, we designed an ASO targeting the F4 domain. To enhance in vivo stability, all pyrimidine bases in both strands of the ASO were modified with 2’‐O‐methyl (2’‐OMe) (Figure 8F). RIP assays confirmed that LNP‐delivered ASO (LNP‐ASO‐*Lincmd1*) efficiently disrupted the *Lincmd1*–*Dzip3* interaction, whereas LNP‐empty and LNP‐Scramble‐ASO had no effect (Figure S9C). In vitro, co‐culture of metabolically stimulated Hepa1‐6 cells with CD8^+^ T cells demonstrated that LNP‐ASO‐*Lincmd1* treatment significantly enhanced CD8^+^ T cell–mediated cytotoxicity, as shown by CCK‐8 and colony formation assays (Figure S9D,E). Living imaging revealed that, following tail vein injection, LNP‐ASO‐*Lincmd1* preferentially accumulated in the liver (Figure 8G). To evaluate potential off‐target effects, we performed RNA‐seq on MASH‐HCC tumors treated with LNP‐Empty, LNP‐Scramble‐ASO, or LNP‐ASO‐*Lincmd1*. Principal component analysis demonstrated minimal transcriptomic differences between the LNP‐Empty and LNP‐Scramble‐ASO groups (Figure S9F). GSEA revealed selective activation of the p53 signaling pathway and the antigen presentation pathway in LNP‐ASO‐*Lincmd1*–treated group, with no evidence of widespread transcriptomic perturbation, indicating minimal off‐target effects (Figure 8H; Figure S9G). H&E staining and serum biochemical analyses further confirmed the absence of hepatotoxicity, nephrotoxicity, or cardiotoxicity (Figure S9H–N). Mechanistically, LNP‐ASO‐*Lincmd1* treatment promoted the relocalization of *Dzip3* from the nucleus to the cytoplasm, thereby enhancing its interaction with *Octn2* and facilitating K48‐linked ubiquitination and degradation of *Octn2* (Figure 8I). This led to a marked reduction in intracellular L‐carnitine and ALCAR, a concomitant increase in acetyl‐CoA levels, and elevated protein pan‐acetylation levels (Figure S10A–D). Enhanced acetylation of both p53 and histone H3 was also observed, resulting in activation of the p53 tumor suppressive pathway and the MHC‐I antigen presentation pathway (Figure S10E–J).

To assess therapeutic efficacy in vivo, we treated MASH‐HCC mice with anti–PD‐1 antibodies (Figure 8J). No significant differences in body, liver, or spleen weight were observed across treatment groups (Figure S10K–M). While anti‐PD‐1 monotherapy exhibited limited efficacy and reflected immunotherapy resistance in MASH‐HCC, treatment with LNP‐ASO‐*Lincmd1* alone significantly reduced tumor burden, suppressed tumor cell proliferation, and extended survival. Notably, the combination of LNP‐ASO‐*Lincmd1* with anti‐PD‐1 therapy further amplified these effects, resulting in the most substantial tumor inhibition and the greatest improvement in overall survival (Figure 8L–O).

To enhance clinical relevance, we next established a humanized patient‐derived xenograft (hu‐PDX) model based on huHSC‐NCG‐hIL15 mice. Briefly, NCG‐hIL15 immunodeficient mice were reconstituted with human CD34^+^ hematopoietic stem cells to generate huHSC‐NCG‐hIL15 humanized mice that develop functional human T cells and NK cells, which are central mediators of antitumor immunity. Following confirmation of stable engraftment and functional activity of these human immune effector cells, freshly resected MASH‐HCC tumor specimens were engrafted subcutaneously into these mice, resulting in the successful establishment of huHSC‐NCG‐hIL15–based MASH‐HCC hu‐PDX models, which enable evaluation of immunotherapeutic responses in a human immune context. Consistent with findings in the MASH‐HCC mouse model, anti–PD‐1 monotherapy exhibited minimal antitumor activity in hu‐PDX tumors. In contrast, combined treatment with anti–PD‐1 and LNP‐ASO‐LINCMD1 significantly reduced tumor volume, maximal diameter, and tumor growth rate, demonstrating a marked enhancement of immunotherapeutic efficacy in this clinically relevant humanized model (Figure 8P–S).

These findings highlight the therapeutic potential of targeting the LINCMD1‐DZIP3‐OCTN2 axis using LNP‐ASO‐LINCMD1 to enhance the efficacy of anti‐PD‐1 immunotherapy in MASH‐HCC.

## 3 Discussion

Over the past decade, driven by the increasing global burden of obesity, metabolic dysfunction‐associated steatohepatitis (MASH) has rapidly emerged as the leading contributor to the rising incidence of hepatocellular carcinoma (HCC) [40]. Compared to HCCs arising from other etiologies, MASH‐HCC exhibits distinct features. First, inflammation within the MASH‐HCC microenvironment is characterized by persistent, chronic activation, which markedly differs from that in other HCC subtypes [41]. Second, MASH‐HCC displays a unique lipid metabolism profile, marked by reprogramming of lipogenesis pathways, enhanced de novo fatty acid (FA) synthesis, and impaired FA oxidation (FAO) [42]. Additionally, alterations in the tumor microenvironment and potential shifts in energy utilization may drive MASH‐HCC away from the classical Warburg effect typically seen in many cancers [6]. Multiple studies, including our previous work, have demonstrated that FAO is significantly inhibited in MASH‐HCC, contributing to lipid accumulation in hepatocytes and creating a metabolically permissive niche for tumorigenesis [9, 10, 11]. These findings are in line with our current study, in which we observed that although L‐carnitine–a well‐established initiator of FAO [17]–was significantly elevated in MASH‐HCC, its increase did not enhance FAO activity in these cells, further confirming the suppression of FAO in MASH‐HCC.

Fujiwara et al. reported that in MASH‐HCC, CPT2 is markedly downregulated. As a key FAO mitochondrial enzyme that converts long‐chain acylcarnitines back to acyl‐CoA for β‐oxidation, CPT2 suppression leads to impaired FAO and accumulation of L‐carnitine–related metabolites [13]. Consistent with this, we also observed significant CPT2 suppression in MASH‐HCC, supporting a model in which disruption of canonical FAO contributes to L‐carnitine buildup. In addition, our findings extend beyond passive accumulation: we uncovered a striking activation of carnitine uptake machinery in MASH‐HCC, indicating that L‐carnitine elevation is an actively maintained metabolic state rather than merely a consequence of FAO inhibition. Classically, L‐carnitine shuttles long‐chain fatty acids into mitochondria for β‐oxidation, fueling ATP production and energy homeostasis. Additionally, L‐carnitine can bind to various short‐chain acyl groups, particularly acetyl groups, thus serving as an important regulator of acetyl group homeostasis [18]. Therefore, in the FAO‐suppressed state characteristic of MASH‐HCC, it is plausible that L‐carnitine may be diverted from its canonical FAO role toward an alternative function in buffering acetyl groups. Our current study confirmed this hypothesis by demonstrating that L‐carnitine accumulation in MASH‐HCC is linked to acetyl group buffering rather than FAO enhancement. Based on these findings, we propose a novel metabolic bypass hypothesis in MASH‐HCC: under conditions of impaired FAO, excess L‐carnitine is redirected to buffer acetyl groups, facilitating tumor progression. Furthermore, this discovery raises intriguing questions about whether other metabolic pathways might also be reprogrammed in MASH‐HCC to compensate for the suppressed FAO. For instance, glycolysis, amino acid metabolism, or alternative lipid pathways could potentially become upregulated, contributing to cellular energy balance and tumor growth. Elucidating these potential bypass mechanisms will be critical for a comprehensive understanding of metabolic plasticity in MASH‐HCC and for developing targeted therapeutic strategies.

Further investigation revealed that elevated L‐carnitine in MASH‐HCC contributes to acetyl group buffering, leading to the depletion of intracellular acetyl‐CoA pools. Notably, acetyl‐CoA is not only a key intermediate in energy metabolism–including β‐oxidation, glycolysis, and amino acid catabolism–but also serves as the principal donor for protein acetylation, thereby influencing a variety of epigenetic and post‐translational modifications critical for tumor suppression [14, 15]. Our subsequent experiments demonstrated that reduced acetyl‐CoA levels in MASH‐HCC cells were associated with decreased acetylation of crucial tumor suppressors such as p53, leading to diminished transcriptional activity and enhanced tumor progression. These findings emphasize the critical role of metabolic‐epigenetic cross‐talk in cancer progression.

Meanwhile, MASH‐HCC is increasingly recognized as an immunologically “cold” tumor subtype, exhibiting poor response to immune checkpoint blockade [5]. Importantly, multiple large‐scale clinical studies have demonstrated that patients with MASH‐driven HCC derive little to no survival benefit from PD‐1/PD‐L1 blockade, unlike patients with viral HCC who show clear therapeutic responses, firmly establishing MASH‐HCC as a clinically validated immunotherapy‐resistant subtype [1, 4, 5]. Unlike virally driven HCCs, MASH‐HCC develops in a fibrotic and lipotoxic hepatic microenvironment characterized by chronic inflammation, myeloid cell enrichment, and T cell exclusion, all of which contribute to impaired antigen presentation and reduced cytotoxic T cell infiltration [4]. This immunosuppressive landscape likely underlies the limited efficacy of anti–PD‐1/PD‐L1 therapies in MASH‐HCC patients [1]. Our results reveal that elevated intracellular L‐carnitine levels in MASH‐HCC disrupt immune surveillance by depleting acetyl groups required for protein acetylation. This depletion impairs activation of the MHC‐I antigen processing and presentation pathway by suppressing H3 acetylation. Consistent with previous studies and our findings, loss of MHC‐I pathway activity substantially diminishes tumor cell visibility to cytotoxic CD8^+^ T lymphocytes, thereby promoting immune evasion [43]. This metabolic‐epigenetic interference shapes an immunologically silent phenotype that not only facilitates tumor progression but also confers resistance to immune checkpoint therapy.

To explain the accumulation of L‐carnitine, we observed a significant upregulation of its key transporter, OCTN2, in MASH‐HCC. Interestingly, previous studies have predominantly linked OCTN2 overexpression to enhanced FAO [8, 44], yet our observation contrasts sharply with the suppressed FAO in MASH‐HCC, highlighting a unique metabolic adaptation. This discrepancy prompted us to further investigate the underlying regulatory mechanisms driving OCTN2 upregulation under FAO‐suppressed conditions.

To further investigate the mechanism driving upregulation of the carnitine transporter OCTN2 in MASH‐HCC, we examined its expression. Interestingly, OCTN2 protein levels were markedly elevated despite minimal changes in its mRNA expression, suggesting post‐translational regulation. Integrating multi‐omics analyses with experimental validation, we found that the E3 ubiquitin ligase DZIP3 exhibited reduced interaction with OCTN2 in MASH‐HCC, resulting in decreased K48‐linked ubiquitination and diminished proteasomal degradation of OCTN2. Further mechanistic exploration identified the long non‐coding RNA LINCMD1—not a protein—as the upstream regulator. LINCMD1 competitively binds to DZIP3, sequestering it in the nucleus and thereby preventing its interaction with OCTN2 in the cytoplasm, ultimately stabilizing OCTN2 protein levels. This finding expands our understanding of the molecular mechanisms regulating OCTN2 expression, explaining its sustained activation in MASH‐HCC despite suppressed FAO. Traditionally, lncRNAs regulate gene expression through diverse mechanisms such as chromatin remodeling, transcriptional modulation, RNA sponging, and control of protein stability [36, 45]. In the context of MASH‐HCC, several lncRNAs—including LINC01468 [46], LncARSR [47], and lnc‐OXAR [48]—have been shown to remodel lipid metabolism, alter fatty acid utilization, or influence ubiquitin‐dependent protein turnover. These studies underscore the central role of lncRNAs in metabolic reprogramming within tumors and highlight LINCMD1 as a previously unrecognized, newly identified regulator that expands this emerging class of metabolic modulators in MASH‐HCC.

Building on this mechanistic insight into the LINCMD1/DZIP3/OCTN2 axis, we next explored its therapeutic potential in MASH‐HCC. OCTN2 dysregulation has been implicated in several severe disorders, including primary systemic carnitine deficiency, secondary cardiomyopathy, and epilepsy [39]. Moreover, no approved or investigational drugs currently target the E3 ligase DZIP3 specifically. Given these challenges, we shifted our focus to LINCMD1 as a therapeutic entry point. RNA‐based therapies offer several key advantages, including high sequence specificity, chemical modifiability, and compatibility with advanced delivery platforms such as lipid nanoparticles (LNPs) [49]. To selectively disrupt the LINCMD1‐DZIP3 interaction without altering overall LINCMD1 expression, we designed antisense oligonucleotides (ASOs) targeting the DZIP3‐binding region of LINCMD1 rather than employing shRNA or siRNA approaches. We encapsulated these ASOs using an FDA‐approved LNP formulation to ensure efficient and liver‐specific delivery in vivo. Remarkably, treatment with LINCMD1‐targeting LNP‐ASOs significantly reduced OCTN2 protein expression, alleviated intracellular L‐carnitine accumulation, and restored the activity of both the p53 signaling and MHC‐I antigen presentation pathways. Furthermore, combination therapy with LNP‐ASO‐LINCMD1 and anti‐PD‐1 antibody substantially enhanced antitumor efficacy, suggesting that targeting this metabolic‐epigenetic axis can effectively sensitize MASH‐HCC to immune checkpoint blockade.

Therefore, targeting the LINCMD1/DZIP3/OCTN2‐L‐carnitine axis may thus represent a novel strategy to interrupt the metabolic adaptations that sustain tumor growth in MASH‐HCC. Moreover, this regulatory axis underscores the critical role of metabolic–epigenetic–immune crosstalk in cancer biology. Nevertheless, several limitations should be acknowledged. First, although our study incorporated both orthotopic and humanized PDX models, these systems cannot fully reproduce the complex metabolic, fibrotic, and immunologic landscape of human MASH. Second, due to ethical constraints, the therapeutic efficacy of LNP‐ASO‐LINCMD1 could not be assessed in human subjects. Third, while our findings establish a noncanonical metabolic role for L‐carnitine, further work is required to delineate how this pathway integrates with other metabolic nodes dysregulated in MASH‐HCC, including glycolysis, amino‐acid metabolism, and related metabolic circuits. Clarifying how these pathways converge or compensate for one another will be essential for refining therapeutic strategies.

In conclusion, our study uncovers a previously unrecognized metabolic adaptation in MASH‐HCC, whereby elevated L‐carnitine is redirected from FAO to intracellular acetyl group buffering, leading to suppressed p53 signaling and impaired MHC‐I antigen presentation‐thereby promoting tumor progression and immune evasion. This reprogramming is orchestrated by the LINCMD1/DZIP3/OCTN2 axis: LINCMD1 competitively binds DZIP3 and retains it in the nucleus, preventing its interaction with cytoplasmic OCTN2. This disrupts DZIP3‐mediated K48‐linked ubiquitination, resulting in OCTN2 protein stabilization and increased intracellular L‐carnitine accumulation. To therapeutically exploit this mechanism, we developed liver‐targeted, LNP‐encapsulated ASOs against LINCMD1, which significantly sensitized tumors to anti‐PD‐1 therapy. These findings not only advance our understanding of MASH‐HCC pathogenesis but also identify a promising metabolic‐epigenetic‐immune axis for therapeutic intervention.

## 4 Materials and Methods

### 4.1 Clinical Specimens

Tumor and matched adjacent non‐tumor tissues were collected from 63 patients who underwent surgical resection for HCC at The Second Affiliated Hospital of Kunming Medical University, West China Hospital of Sichuan University, and The Second Affiliated Hospital of Nanchang University. The cohort included 41 patients with MASH‐HCC and 22 patients with non‐MASH‐HCC, comprising 19 females and 44 males aged between 40 and 65 years (Tables S1 and S2). All specimens underwent independent pathological review by two experienced pathologists blinded to clinical information to ensure diagnostic consistency. The diagnosis of MASH‐HCC was established based on both histological and clinical criteria. Histologically, MASH was defined according to the Kleiner scoring system, with a nonalcoholic steatohepatitis activity score (NAS) ≥5, or NAS scores of 3–4 accompanied by stage ≥1 fibrosis [2]. Clinically, patients exhibited one or more features of metabolic dysfunction, including overweight/obesity, type 2 diabetes mellitus, or another metabolic dysregulation [50]. Patients with significant alcohol intake (>140 g/week for males or >70 g/week for females), drug‐induced liver injury, or positive hepatitis B or C viral markers were excluded from the MASH‐HCC group. Conversely, the non‐MASH‐HCC group included patients with histologically confirmed HCC without steatohepatitis (NAS <3) and without metabolic dysfunction, mainly of viral (HBV/HCV‐related) etiology, serving as non‐metabolic controls. Patients with other potential causes of liver disease, such as autoimmune hepatitis, alcoholic liver disease, or genetic/metabolic disorders, were excluded from both groups to ensure accurate classification. All participants provided written informed consent prior to inclusion. The study protocol was conducted in accordance with the Declaration of Helsinki and approved by the Ethics Committees of all participating centers (#Review‐PJ‐Science‐2024‐186).

### 4.2 Mouse Models

All animal procedures were approved by the Institutional Animal Care and Use Committee of Kunming Medical University (#kmmu20241594) and conducted in accordance with the Guide for the Care and Use of Laboratory Animals. Male C57BL/6J mice (8–10 weeks old) were housed in a temperature‐controlled environment (23°C ± 2°C) under a 12 h light/dark cycle. A spontaneous liver cancer model was established by hydrodynamic tail vein injection (HTVi) of oncogenic genes (Myc/Ras) together with the Sleeping Beauty transposase at 16 weeks. The experimental group was fed a high‐fat/high‐cholesterol (HFHC) diet (TP26304, Trophic Diet, Nantong, China) consisting of 14 % protein, 42 % fat, 44 % carbohydrates, and 0.2 % cholesterol to establish a MASH‐HCC model. In contrast, the control group received a normal chow (NC) diet (XTI01WC‐010, Xietong, Jiangsu, China) containing 18.3 % protein, 10.2 % fat, and 71.5 % carbohydrates in the non‐MASH‐HCC model.

To achieve hepatocyte‐specific overexpression or knockdown of *Octn2*, adeno‐associated virus (AAV) vectors‐AAV8‐TBG‐ZsGreen‐*Octn2* and AAV8‐TBG‐ZsGreen‐sh*Octn2*‐were constructed using the thyroxine‐binding globulin (TBG) promoter to ensure liver‐specific expression. The primer sequences for *Octn2* and sh*Octn2* are listed in Table S6. At week 17 of the HFHC feeding protocol, mice were intravenously injected via the tail vein with 1 × 10^1^ ^1^ genome copies of the respective AAV particles. Control mice on the same HFHC diet received AAV8‐TBG‐ZsGreen‐*Ctrl* or AAV8‐TBG‐ZsGreen‐shNC as negative control vectors. For each experiment, mice were randomly assigned to treatment groups (n = 5 per group). At the experimental endpoint, mice were euthanized, and livers were excised, rinsed in PBS, and photographed on a calibrated background. Tumor nodules were initially identified by gross inspection as well‐demarcated pale or whitish lesions on the liver surface, followed by histological confirmation on representative H&E‐stained liver sections to verify malignant features. The maximal tumor diameter was determined by measuring the longest axis of the largest nodule using digital calipers. All measurements were independently performed by two investigators blinded to group allocation.

To generate cell‐derived xenograft (CDX) models, we first established HuH‐7 cell lines with stable overexpression or knockdown of the indicated genes. A total of 1 × 10^6^ genetically modified HuH‐7 cells were then resuspended in PBS/Matrigel (1:1) and injected subcutaneously into the flanks of BALB/c nude mice (4–5 weeks old, 18–22 g). Mice were randomly assigned to the designated treatment groups (n = 5 per group). For patient‐derived xenograft (PDX) studies, huHSC‐NCG‐hIL15 humanized mice were generated by transplanting human CD34^+^ hematopoietic stem cells into NCG‐hIL15 immunodeficient recipient mice (T066463, Gempharmatech, Jiangsu, China) following preconditioning, resulting in the development of functional human T‐cell and NK‐cell populations, which are key mediators of antitumor immunity. After confirmation of stable engraftment and functional activity of these human immune effector cells, freshly resected MASH‐HCC tumor specimens obtained from patients undergoing surgical treatment were cut into 2–3 mm^3^ fragments and implanted subcutaneously into the flanks of the humanized mice. This huHSC‐NCG‐hIL15–based PDX model enables evaluation of immunotherapeutic responses in a human immune cell–supported context. Once tumors reached approximately 100–150 mm^3^, mice were randomly assigned to treatment groups (n = 5 per group). To mimic the metabolic‐inflammatory environment characteristic of MASH‐HCC, both nude and humanized mice were fed on an HFHC diet throughout the experimental period. Tumor dimensions were measured every three days using digital calipers, and tumor volume was calculated as V = (length × width^2^) / 2. Animals were euthanized three weeks after inoculation or treatment, and tumor weight, maximum diameter, and volume were recorded. All tumor measurements and analyses were performed by investigators blinded to group allocation.

### 4.3 In Vivo Experiments and LNP‐ASO Treatment

In the AAV8‐TBG‐Ctrl and AAV8‐TBG‐*Octn2* groups, to inhibit *p53* activity in vivo, mice were administered PFT‐α (2.2 mg/kg, intraperitoneally, three times per week) starting one day after AAV8 injection at week 17, while control mice received vehicle (DMSO diluted in physiological saline) [51].

In the AAV8‐TBG‐shNC and AAV8‐TBG‐sh*Octn2* groups, to inhibit histone deacetylase (HDAC) activity in vivo, mice were administered trichostatin A (TSA; 1 mg/kg, intraperitoneally, every other day) starting one day after AAV8 injection at week 17, while control mice received vehicle (DMSO diluted in physiological saline).

In the AAV8‐TBG‐Ctrl and AAV8‐TBG‐sh*Octn2* groups, to evaluate the synergistic effect of immunotherapy, mice were treated with anti‐PD‐1 antibody (300 µg, intraperitoneally, every 5 days) starting at week 20. Mice in the control group received IgG isotype control using the same injection schedule.

Antisense oligonucleotides (ASOs) specifically targeting the DZIP3‐binding region of LINCMD1 were synthesized by Tsingke Biotechnology Co., Ltd. (Beijing, China), with all pyrimidine bases modified by 2’‐O‐methyl (2’‐OMe). The mouse‐specific ASO sequence was 5′‐CAUAAGCAAGGCUGUA‐3′, while the human‐specific ASO sequence was 5′‐CAACAAUUUCAAUGAU‐3′. Lipid nanoparticles (LNPs) encapsulating ASOs or left empty (LNP‐ Empty) were formulated by Guangzhou Kelan Biotechnology Co., Ltd. (Guangzhou, China). For treatment, MASH‐HCC mice and humanized PDX mice were randomly divided into LNP‐ASO‐LINCMD1 and LNP‐Empty groups. LNPs were administered via tail vein injection at a dose of 1 mg/kg every 3 days starting at week 20.

### 4.4 Cell Culture and Treatment

The normal human hepatocyte cell line THLE‐2 (CL‐0833), the human HCC cell lines HuH‐7 (CL‐0120), Li‐7 (CL‐0139), Mahlavu (CL‐0735), HepG2 (CL‐0103), and the murine HCC cell line Hepa1‐6 (CL‐0105) were obtained from Wuhan Pricella Biotechnology Co., Ltd. Cells were routinely cultured in either Dulbecco's Modified Eagle Medium (DMEM) or RPMI‐1640 medium supplemented with 10 % fetal bovine serum (FBS), 1 % penicillin‐streptomycin, and maintained at 37°C in an atmosphere containing 5 % CO_2_. Because this study focuses on the function of the carnitine transporter OCTN2 and conventional culture media contain negligible or only trace amounts of L‐carnitine, L‐carnitine was supplemented to the culture medium at 50 µM. This concentration approximates physiological plasma levels of free L‐carnitine and did not affect cell viability or proliferation in preliminary assays.

To recapitulate hepatic lipid accumulation and steatosis observed in vivo and to concomitantly model the inflammatory milieu, an in vitro MASH‐HCC model was established by incubating cells with palmitic acid (PA, 0.4 mM) and oleic acid (OA, 0.8 mM) together with IL‐6 (10 ng/mL), TNF‐α (10 ng/mL), and LPS (100 ng/mL); fatty acid‐free BSA served as the control treatment.

To inhibit fatty acid oxidation (FAO) in the MASH‐HCC cell model, cells were treated with β‐oxidation inhibitor Trimetazidine (TMZ, 1 mM) (5011‐34‐7, MCE, USA) dissolved in dimethyl sulfoxide (DMSO). An equivalent volume of DMSO was added to another group as a control.

### 4.5 Plasmid Construction and Lentiviral Packaging

Full‐length coding sequences of target genes were amplified and cloned into the pcDNA3.1(+) expression vector using standard molecular cloning techniques to generate overexpression plasmids. For gene knockdown, specific short hairpin RNA (shRNA) sequences targeting the genes of interest were designed and inserted into the pGPU6‐GFP‐Neo vector for transient transfection experiments. For stable gene expression or silencing, coding sequences were re‐cloned into the pCDH‐CMV‐MCS‐EF1α‐Puro lentiviral vector. Separately, shRNA target sequences were synthesized and inserted into the pLKO.1‐puro vector to construct lentiviral knockdown plasmids. Lentiviral particles were produced by co‐transfecting HEK 293T cells with each transfer plasmid and the second‐generation packaging plasmids psPAX2 and pMD2.G, using Neofect transfection reagent (Neofect, Beijing, China). Viral supernatants were harvested at 48 and 72 h post‐transfection, filtered through a 0.45 µm membrane, and used for infection of target cells. The sequences of the primers employed are detailed in Table S6.

### 4.6 Western Blotting

Total protein was extracted from human and mouse hepatic tissues, as well as cultured cell lines, using radioimmunoprecipitation assay (RIPA) buffer comprising 65 mM Tris‐HCl (pH 7.5), 150 mM NaCl, 1 mM EDTA, 1 % Nonidet P‐40, 0.5 % sodium deoxycholate, and 0.1 % SDS. The lysis buffer was supplemented with protease inhibitor cocktail (04693132001, Roche) and phosphatase inhibitor tablets (4906837001, Roche) to preserve protein integrity. Protein concentrations were quantified using a bicinchoninic acid (BCA) protein assay kit (23225, Thermo Fisher Scientific). For protein stability analysis, cells were treated with cycloheximide (CHX, 20 µg/mL; Sigma–Aldrich, USA) for the indicated durations to inhibit de novo protein synthesis. Cell lysates collected at each time point were subjected to Western blotting to evaluate the degradation kinetics of the target proteins. For protein solubility assays, cells were washed twice with PBS and lysed in ice‐cold RIPA buffer supplemented with phosphatase inhibitors for 30 min. Lysates were centrifuged at 12 000 rpm for 15 min at 4°C to separate the soluble supernatant from the insoluble pellet. The supernatant fraction was collected, whereas the insoluble pellet was washed once with RIPA buffer and subsequently resuspended in loading buffer. Both fractions were denatured by boiling at 100°C prior to Western blotting analysis.

Equal amounts of protein were resolved by SDS‐polyacrylamide gel electrophoresis (SDS‐PAGE) using either 8 % or 10 % gels and subsequently transferred onto polyvinylidene difluoride (PVDF) membranes (Millipore). Membranes were blocked with 5 % skimmed milk in TBST for 1 h at room temperature to reduce nonspecific binding, followed by overnight incubation at 4°C with primary antibodies. After washing, membranes were probed with species‐specific horseradish peroxidase (HRP)‐conjugated secondary antibodies. Immunoreactive bands were visualized using enhanced chemiluminescence (ECL) reagents (170‐5061, Bio‐Rad) and detected with the ChemiDoc MP Imaging System (Bio‐Rad, Hercules, CA, USA). A full list of primary and secondary antibodies employed in this study is provided in Table S7.

### 4.7 Untargeted Metabolomics Analysis

Untargeted metabolomics profiling was performed on six human hepatocellular carcinoma (HCC) tissue samples, including three MASH‐HCC and three non‐MASH‐HCC cases. Frozen tumor tissues (∼20 mg each) were homogenized under liquid nitrogen and extracted with 400 µL of pre‐chilled methanol:water (7:3, v/v) containing internal standards. After vortexing (1500 rpm, 5 min) and incubation on ice (15 min), the samples were centrifuged at 12 000 rpm for 10 min at 4°C. The supernatants were then incubated at −20°C for 30 min and centrifuged again (12 000 rpm, 3 min, 4°C), and 200 µL of the clarified extract was used for analysis.

Liquid chromatography–tandem mass spectrometry (LC‐MS/MS) and data processing were performed by Metware Biotechnology Co., Ltd. (Wuhan, China). Chromatographic separation was carried out on a Waters ACQUITY UPLC HSS T3 C18 column (1.8 µm, 2.1 mm × 100 mm) using a binary gradient of 0.1 % formic acid in water (solvent A) and 0.1 % formic acid in acetonitrile (solvent B). Metabolites were detected using a Triple TOF 6600 system (AB Sciex) operated in both positive and negative ionization modes.

Raw data were converted to mzML format using ProteoWizard and processed with XCMS for peak detection, alignment, and area correction. Metabolites detected in fewer than 50 % of samples per group were removed. Annotation was performed using public spectral libraries and the metDNA platform. Multivariate (PCA, OPLS‐DA) and univariate (Student's *t*‐test, fold change, VIP score) statistical analyses were conducted using the R package MetaboAnalystR, with pathway enrichment based on KEGG and HMDB databases.

### 4.8 Immunoprecipitation (IP) and Mass Spectrometry (MS)

HEK 293T and HuH‐7 cells were co‐transfected with the appropriate plasmids and incubated for 36 h prior to lysis. Cell lysates were homogenized under constant agitation and centrifuged to remove debris. For each immunoprecipitation (IP), 500 µL of the supernatant was incubated overnight at 4°C with 20 µL of Protein A/G magnetic beads (HY‐K0202, MCE, USA) and 1 µg of the corresponding antibody on a rocking platform. Beads were washed with high‐salt buffer, and bound proteins were eluted with SDS loading buffer and boiled at 96°C for 10 min. Eluted proteins and whole‐cell lysates were analyzed by SDS‐PAGE and immunoblotting with specific antibodies.

For IP‐MS, HuH‐7 cells cultured under PA/OA conditions were transfected with Flag‐tagged OCTN2 or HA‐tagged DZIP3 plasmids. Immunoprecipitation was performed using anti‐Flag magnetic beads (HY‐K0207, MCE, USA) or anti‐HA magnetic beads (HY‐K0201, MCE, USA). Eluted proteins were separated by SDS‐PAGE and visualized by silver staining using the Pierce Silver Stain Kit (24600, Thermo Fisher, USA). Bands of interest were excised and subjected to LC‐MS/MS analysis for protein identification.

### 4.9 Quantitative Proteomic Analysis and Acetylome Profiling

Label‐free quantitative proteomic profiling was performed on six PA/OA‐treated HuH‐7 cell samples, including three with shOCTN2 transfection and three shNC controls. Sample preparation, LC‐MS/MS acquisition, and subsequent bioinformatic analyses were conducted using a quantitative proteomics workflow.

Cells were lysed in a buffer containing SDS, Tris‐HCl, urea, thiourea, and protease inhibitors. Total proteins were extracted, reduced with dithiothreitol (DTT), alkylated with iodoacetamide (IAA), and precipitated using pre‐chilled acetone. The precipitated proteins were resolubilized and enzymatically digested overnight with trypsin. Peptide mixtures were desalted using C18 cartridges, dried under vacuum, and reconstituted prior to LC‐MS/MS analysis. Peptides were separated on a nanoElute UHPLC system and analyzed using a Bruker timsTOF Pro2 mass spectrometer operating in PASEF mode. Raw data were processed using FragPipe (v22.0), with MSFragger for peptide identification and IonQuant for label‐free quantification. Protein identification was performed against the UniProt human protein database. Search parameters included carbamidomethylation (C) as a fixed modification, and oxidation (M) and acetylation (protein N‐term) as variable modifications. A false discovery rate (FDR) <1 % was applied at both the peptide and protein levels. Differentially expressed proteins were determined using fold change (≥1.5 or ≤0.667) and p < 0.05. Subsequent Gene Ontology (GO), KEGG pathway, and protein domain enrichment analyses were performed using R.

For acetylome profiling, PA/OA‐treated HuH‐7 cells were processed identically to the proteomic samples up to the stage of total protein extraction. Following lysis and clarification, equal amounts of protein from each sample were subjected to IP using anti–acetylated lysine antibody (9814, CST, USA) together with Protein A/G magnetic beads (HY‐K0202, MCE, USA), following the same IP procedure described above. The enriched acetylated proteins were submitted directly for LC–MS/MS analysis using the same instrumentation and data‐processing workflow as the global proteome samples.

### 4.10 RNA Immunoprecipitation (RIP) and ncRNA Sequencing

To investigate RNA species interacting with DZIP3, RNA immunoprecipitation (RIP) combined with high‐throughput sequencing was performed. PA/OA‐treated HuH‐7 were lysed in RIP buffer supplemented with RNase inhibitors. Lysates were incubated with an anti‐DZIP3 antibody (Table S7) and Protein A/G magnetic beads (HY‐K0202, MCE, USA) overnight at 4°C with gentle rotation. Hepa1‐6 cells were co‐transfected with Flag‐Dzip3‐WT or Flag‐Dzip3‐ΔKR, followed by IP using anti‐Flag magnetic beads (HY‐K0207, MCE, USA). An anti‐mouse IgG antibody served as a negative control. Following magnetic separation, the RNA–protein complexes were washed, and the bound RNAs were eluted.

One portion of the purified RNAs was subjected to non‐coding RNA (ncRNA) sequencing. The sequencing library was constructed using the Ribo‐Zero rRNA removal method to enrich for ncRNAs, and sequencing was performed on the Illumina NovaSeq platform (PE150). Clean reads were aligned to the human reference genome using HISAT2, and transcript quantification was conducted using StringTie. Differentially enriched transcripts were identified with DESeq2 based on TPM values.

In parallel, another portion of the immunoprecipitated RNA was reverse transcribed and subjected to qRT‐PCR to detect LINCMD1 enrichment.

### 4.11 RNA‐pull Down

LINCMD1 was transcribed and amplified in vitro using the MegaScript T7 Transcription Kit Plus (AM1333, Thermo Fisher, USA) according to the manufacturer's instructions. Biotin‐labeled LINCMD1 was refolded in NEB enzyme buffer containing RNaseOUT (Invitrogen, USA) to preserve RNA structure. The refolded RNA was then incubated with cell lysates prepared from the indicated target cells in the presence of streptavidin magnetic beads. Lysates were pre‐cleared with beads before incubation to reduce non‐specific binding.

### 4.12 Liquid Chromatography–Tandem Mass Spectrometry (LC–MS/MS)

Equal numbers of cultured tumor cells (approximately 1 × 10^7^ cells) were harvested and rapidly washed with cold PBS to remove residual medium. To analyze fatty acid β‐oxidation intermediates, intracellular metabolites were extracted using a cold methanol‐based protocol. Briefly, cells were resuspended in 1 mL of pre‐chilled 80 % methanol, vortexed vigorously, and incubated on ice for 10 min to quench metabolism. The lysates were centrifuged at 14 000 × g for 10 min at 4°C to remove cell debris, and the supernatants were collected for subsequent analysis. The extracted samples were analyzed by LC‐MS/MS (Agilent 1290 Infinity II coupled with an Agilent 6470 triple quadrupole, Agilent Technologies, USA) equipped with a reversed‐phase C18 column for targeted detection of fatty acid β‐oxidation intermediates. LC‐MS/MS analysis and subsequent statistical analysis were performed according to established standard protocols.

### 4.13 Mitochondrial Enzymatic Activity Assays

Mitochondria were isolated from liver tissues of non‐MASH‐HCC and MASH‐HCC mouse models using a Mitochondrial Isolation and Protein Extraction Kit (PK10016, Proteintech, China) following the manufacturer's protocol. Briefly, liver samples were homogenized and subjected to sequential centrifugation steps to obtain crude and subsequently high‐purity mitochondrial fractions. Mitochondrial integrity and purity were verified using Janus Green B staining. A portion of the mitochondrial suspension was lysed using the mitochondrial lysis buffer included in the kit, and mitochondrial protein concentrations were determined by BCA.

For enzymatic assays, equal amounts of mitochondria (0.5 mg/mL) were incubated with the respective substrates in reaction buffer. CPT2 activity was measured by incubating mitochondria with palmitoyl‐carnitine (50 µM) and CoA‐SH (0.5 mM), whereas CRAT activity was assessed using acetyl‐CoA (0.3 mM) and L‐carnitine (5 mM) under identical conditions. Reactions were performed at 30°C for 5 min. The formation of palmitoyl‐CoA was quantified by LC–MS to determine CPT2 activity. The production of acetyl‐carnitine (ALCAR) was similarly quantified to measure CRAT activity.

Enzymatic activities were calculated based on product formation and expressed as pmol product/ (min·mg mitochondrial protein). Heat‐inactivated mitochondrial samples were used as negative controls to correct for non‐enzymatic background.

### 4.14 Flow Cytometric Analysis

Fresh tumor tissues were excised and immediately transferred into pre‐chilled PBS to remove blood and necrotic material. Tissues were minced into 1–2 mm^3^ fragments using sterile scissors and enzymatically dissociated in digestion buffer containing collagenase type IV (1 mg/mL) and DNase I (100 U/mL). Samples were incubated at 37°C on a horizontal shaker for 30–60 min, with gentle pipetting every 15 min to facilitate single‐cell release. The digested suspension was passed through a 70 µm cell strainer to obtain single‐cell suspensions, followed by centrifugation (500 × g, 5 min, 4°C). Red blood cells were removed by incubation with RBC lysis buffer at room temperature for 5–10 min. After washing twice with PBS, cells were resuspended in PBS containing 2 % FBS and adjusted to a density of 1 × 10^6^ cells/mL.

To exclude dead cells, single‐cell suspensions were incubated with a viability dye for 10 min at room temperature, washed, and resuspended in staining buffer. Surface staining was performed by incubating cells with fluorochrome‐conjugated antibodies against CD3, CD8, TIM3, and PD1 for 30 min on ice in the dark. After two washes, cells were fixed and permeabilized using fixation/permeabilization buffer according to the manufacturer's instructions. Intracellular staining was then performed by incubating permeabilized cells with antibodies against IFN‐γ and Granzyme B for 30 min on ice in the dark. After two additional washes with permeabilization buffer, cells were resuspended in staining buffer for flow‐cytometric acquisition.

Data were collected on a BD flow cytometer and analyzed using FlowJo software. Sequential gating was applied to identify CD45^+^CD3^+^CD8^+^ T cells, followed by quantification of exhaustion markers (TIM3, PD1) and effector‐function markers (IFN‐γ, Granzyme B).

### 4.15 RNA Extraction and Quantitative Reverse‐transcription PCR (qRT‐PCR) Analysis

Total RNA was extracted using TRIzol reagent (T9424, Sigma–Aldrich, USA). Complementary DNA (cDNA) synthesis was conducted via reverse transcription with the HiScript II Q RT SuperMix (R222‐01, Vazyme Biotech Co., Ltd., Nanjing, China). PCR amplification products were detected using SYBR Green PCR Master Mix (04887352001, Roche, Switzerland). The mRNA expression levels of the target genes were normalized to β‐actin expression. The specific primer sequences used in this study are provided in Table S8.

### 4.16 Chromatin Immunoprecipitation (ChIP)

The potential binding of histone H3 to promoter regions of MHC‐I pathway genes (PSMB8, TAP1, HLA‐A, B2M) was assessed in PA/OA‐treated HuH‐7 cells using ChIP assays. Briefly, cells were crosslinked with 1 % formaldehyde, quenched with glycine, and lysed. Chromatin was sonicated to generate DNA fragments ranging from 200 to 1000 bp. Immunoprecipitation was performed using anti‐H3 antibodies or normal IgG as a negative control. After reversal of crosslinking, DNA was purified and analyzed by qRT‐PCR to quantify the enrichment of target gene promoters. Primer sequences and antibody details are provided in Tables S7 and S8.

### 4.17 Histological and Immunohistochemical (IHC) Staining

Liver tissues were collected and processed for histological and immunohistochemical analyses. For routine histopathological evaluation, liver samples were fixed in 4% paraformaldehyde, embedded in paraffin, sectioned at 4 µm, and subjected to hematoxylin and eosin (H&E) staining according to standard protocols to assess tissue architecture and pathological alterations. For lipid deposition analysis, fresh liver tissues were embedded in optimal cutting temperature (OCT) (abs9756, absin, Shanghai, China) compound, cryosectioned, and stained with Oil Red O (HY‐D1168, MCE, USA) to visualize neutral lipid accumulation, followed by hematoxylin counterstaining.

For immunohistochemical (IHC) staining, paraffin sections (4 µm) were deparaffinized and rehydrated, followed by antigen retrieval using pH 9.0 EDTA buffer (C1038, Solarbio, Beijing, China). Endogenous peroxidase activity was quenched with 3% hydrogen peroxide, and sections were incubated overnight at 4°C with the corresponding primary antibodies. Signal detection was carried out using species‐matched Polymer HRP–Goat recombinant secondary antibodies (RGAM011 and RGAR011, Proteintech, Wuhan, China), according to the host species of the primary antibodies; detailed primary and secondary antibodies information is provided in Supplementary Table S7. Immunoreactivity was visualized using DAB (ZLI‐9018, ZSGB‐BIO, Beijing, China) as the chromogen, followed by hematoxylin counterstaining. Images were acquired using a digital slide scanner (Aperio Versa 200, Leica) or a light microscope (ECLIPSE 80i, Nikon, Tokyo, Japan).

### 4.18 Immunofluorescence (IF) Staining and Quantitative Analysis of Subcellular Distribution

For cell immunofluorescence analysis, treated cells were fixed with paraformaldehyde. After fixation, the cells were blocked with 8 % goat serum albumin and permeabilized using 0.2 % Triton X‐100. They were then sequentially incubated with primary antibodies (Table S7), followed by fluorescein‐conjugated secondary antibodies. Nuclei were stained with DAPI, and images were captured using a confocal laser‐scanning microscope (TCS SP8, Leica, Germany).

Quantification of DZIP3 subcellular distribution was performed using ImageJ. Nuclear regions were defined by DAPI staining, and cytoplasmic regions were generated by subtracting nuclear masks from whole‐cell boundaries. The mean fluorescence intensity of DZIP3 within nuclear and cytoplasmic compartments was measured, and the nuclear‐to‐cytoplasmic (N/C) ratio was calculated for 10 cells per group.

### 4.19 Fluorescence In Situ Hybridization (FISH)

Cells were cultured overnight in 6‐well plates, then fixed with 4 % paraformaldehyde for 30 min and permeabilized with 0.5 % Triton X‐100 for 20 min. Following a 10 min wash with 2× SSC, cells were incubated with the LINCMD1 probe (sequence: 5'‐CCCATACATCGTGAAGACTG‐3'), followed by anti‐digoxigenin‐fluorescein Fab fragments (Roche Diagnostics, Indianapolis, IN, USA). Slides were mounted with ProLong Gold Antifade Reagent and counterstained with DAPI (D1306, Thermo Fisher, Shanghai, China). LINCMD1 localization and staining intensity was visualized using a confocal laser‐scanning microscope (TCS SP8, Leica, Germany).

### 4.20 Dual‐Luciferase Reporter Assay

To evaluate the transcriptional activity of the target promoter, DNA fragments containing either the wild‐type (with the native promoter sequence) or mutated promoter sequence (used as a negative control) were cloned into the pGL4 basic vector (Promega, USA), which lacks eukaryotic promoter and enhancer elements. The recombinant plasmids were co‐transfected into cultured cells along with a Renilla luciferase control plasmid using Neofect transfection reagent (Neofect, Beijing, China), serving as an internal control to normalize for transfection efficiency and cell viability. 48 h post‐transfection, cells were lysed using 1× Passive Lysis Buffer. Luciferase activities were measured using the Dual‐Luciferase Reporter Assay System (E1910, Promega, USA) following the manufacturer's instructions. Briefly, firefly luciferase activity was quantified by adding Luciferase Assay Reagent II to the cell lysates. Subsequently, Stop & Glo Reagent was added to quench the firefly signal and simultaneously initiate Renilla luciferase activity. Relative luciferase activity was calculated by normalizing the firefly luminescence to Renilla luminescence, reflecting the transcriptional activity of the promoter under investigation. The specific primer sequences used for this assay are provided in Table S9.

### 4.21 Cell Proliferation

Cell proliferation was evaluated using the Cell Counting Kit‐8 (CCK‐8) (C0042, Beyotime, Shanghai, China) following the manufacturer's protocol.

For EdU incorporation analysis (C0071S, Beyotime, Shanghai, China), approximately 1 × 10^5^ HCC cells were seeded onto glass coverslips in 12‐well plates. After cell attachment, 10 µM EdU was added, and cells were incubated for 2 h. Subsequently, cells were fixed, permeabilized, stained with Hoechst 33342, and imaged using a fluorescence microscope.

For clonogenic assays, logarithmically growing stably transfected and control cells were seeded into 6‐well plates. After 2–3 weeks of culture, colonies were fixed with 4 % paraformaldehyde, stained with crystal violet, and counted to evaluate colony‐forming efficiency.

### 4.22 Cell Invasion and Migration Assay

Transwell invasion assays were conducted using Matrigel‐coated chambers. Growth factor‐reduced Matrigel (diluted in DMEM with 1 % FBS) was added to the upper chambers (80 µL per well) and allowed to gel at 37°C. Following 24 h of serum starvation, 4 × 10^4^ cells were seeded into the upper chambers. The lower chambers were filled with DMEM containing 10 % FBS. After 24 h, cells on the upper membrane surface were removed, and the invaded cells were fixed in methanol, stained with crystal violet, and quantified under a microscope.

For migration assessment, a wound healing (scratch) assay was performed. A linear scratch was introduced into confluent monolayers using a sterile pipette tip. After washing with PBS, cells were cultured in serum‐reduced medium. Wound closure was monitored and imaged at 24, 48, and 72 h to evaluate cell migration capacity.

### 4.23 Biochemical and Metabolite Assays

Serum biochemical parameters, including alanine aminotransferase (ALT), aspartate aminotransferase (AST), blood urea nitrogen (BUN), creatinine (Cr), creatine kinase MB isoenzyme (CK‐MB), and cardiac troponin T (cTnT), were measured using commercial kits from Nanjing Jiancheng Bioengineering Institute, namely the ALT Assay Kit (C009‐2‐1), AST Assay Kit (C010‐2‐1), Urea Assay Kit (C013‐2‐1), Creatinine (Sarcosine Oxidase) Assay Kit (C011‐2‐1), CK‐MB Assay Kit (H197‐1‐1), and Cardiac Troponin Assay Kit (H149‐4‐1), in accordance with the manufacturer's protocols.

Intracellular and intratumoral metabolites were also quantified. L‐carnitine levels were measured using the L‐Carnitine Assay Kit (MAK063, Sigma‐Aldrich, Germany). Acetylcarnitine (ALCAR) concentrations were assessed using the Acetylcarnitine ELISA Kit (CEO400Ge, Cloud‐Clone Corp., Wuhan, China). Pyruvate levels and fatty acid oxidation (FAO) capacity were determined using the Pyruvate Fluorometric Assay Kit (E‐BC‐F058, Elabscience, Wuhan, China) and the FAO Colorimetric Assay Kit (E‐BC‐K318‐M, Elabscience, Wuhan, China), according to the manufacturers’ instructions.

### 4.24 Quantification of Cellular and Subcellular Acetyl‐CoA

Total and compartment‐specific acetyl‐CoA levels were quantified using a commercial colorimetric assay kit (BC0985, Solarbio, Beijing, China). For total cellular acetyl‐CoA, cells were collected, washed twice with ice‐cold PBS, and lysed directly in 80 % ice‐cold methanol to precipitate proteins. After incubation on ice for 10 min, samples were centrifuged (12 000 × g, 10 min, 4°C), and the supernatants were vacuum‐dried and reconstituted in the assay buffer provided by the kit. Acetyl‐CoA levels were measured at 340 nm according to the manufacturer's instructions and normalized to protein concentration. For subcellular quantification, nuclear and cytosolic fractions were isolated under metabolite‐compatible conditions. Briefly, cells were resuspended in ice‐cold hypotonic buffer (10 mM HEPES‐KOH, pH 7.4; 10 mM KCl; 1.5 mM MgCl_2_; protease inhibitor cocktail, without detergents or EDTA) and gently homogenized using a Dounce homogenizer (10–15 strokes). After low‐speed centrifugation (800 × g, 5 min, 4°C), the supernatant was collected as the cytosolic fraction, and the pellet was washed once with the same buffer to obtain the nuclear fraction. Each fraction was immediately extracted and analyzed for acetyl‐CoA using the same kit and protocol described above. Fraction purity was verified by immunoblotting for Lamin A/C (nuclear marker) and GAPDH (cytosolic marker), confirming minimal cross‐contamination. All procedures were carried out on ice or at 4°C to preserve metabolite stability.

### 4.25 Bioinformatics Analysis

All data analyses and visualizations were performed using R software (version 4.4.1) and relevant R packages. Functional enrichment analyses, including KEGG pathway enrichment and Gene Set Enrichment Analysis (GSEA), were conducted using the clusterProfiler package. The GEPIA (Gene Expression Profiling Interactive Analysis) online tool was employed to perform survival analyses by inputting gene names and adjusting appropriate parameters. The iUUCD (Integrative Ubiquitin and Ubiquitin‐like Conjugation Database) was used to identify ubiquitin‐related proteins. In addition, potential protein interactors of DZIP3 were predicted using the STRING (Search Tool for the Retrieval of Interacting Genes/Proteins), BioGRID (Biological General Repository for Interaction Datasets), and IntAct (IntAct Molecular Interaction Database) databases. The RPISeq (RNA–Protein Interaction Prediction) tool was used to predict potential binding fragments of LINCMD1 that interact with DZIP3. URLs of all online tools and databases are provided in Table S10.

### 4.26 Data Statistics

All experiments included at least three biological replicates. Data are presented as mean ± SD. Statistical analyses were performed using Prism 10.0 (GraphPad Software Inc., La Jolla, CA). Comparisons between two groups used unpaired two‐tailed *t*‐tests. Comparisons among three or more groups used one‐way analysis of variance (ANOVA) followed by post hoc Tukey tests. Survival analyses were performed using the Kaplan–Meier method and log‐rank (Mantel–Cox) test to evaluate statistical differences between groups. Statistical significance was defined as ^*^ *p* < 0.05, ^**^ *p* < 0.01, and ^***^ *p* < 0.001. The specific statistical tests applied to each experiment are detailed in the corresponding figure legends.

Additional methodological details are provided in the Supporting Tables.

## Author Contributions

**Chuqi Xia**: Writing – review & editing, Writing – original draft, Visualization, Validation, Methodology, Formal analysis, Data curation, Funding acquisition, Conceptualization. **Xiao Zhang**: Writing – original draft, Validation, Supervision, Resources, Formal analysis, Data curation. **Jinze Li**: Writing – original draft, Methodology, Formal analysis, Data curation. **Ning Xu**: Resources, Data curation. **Sheng Hu**: Resources, Data curation. **Luqi Yu**: Resources, Data curation. **Yuxuan Li**: Software. **Taifu Xiao**: Resources, Data curation. **Xu Li**: Writing – review & editing, Writing – original draft, Resources, Investigation, Formal analysis, Conceptualization. **Xue Wang**: Writing – review & editing, Writing – original draft, Resources, Investigation, Formal analysis, Conceptualization. **Kequan Xu**: Writing – review & editing, Writing – original draft, Visualization, Conceptualization. **Daoming Liang**: Writing – review & editing, Writing – original draft, Supervision, Software, Resources, Project administration, Investigation, Funding acquisition, Conceptualization.

## Funding

Supported by the National Natural Science Foundation of China (82160114), Yunnan Fundamental Research Kunming Medical University Joint Projects (202301AY070001‐025, 202401AY070001‐363), Yunnan Revitalization Talent Support Program (XDYC‐MY‐2022‐0100).

## Ethics Statement

This study was conducted in accordance with the Declaration of Helsinki and was approved by the Ethics Committee of the Second Affiliated Hospital of Kunming Medical University (Approval No. #Review‐PJ‐Science‐2024‐186). All patients provided written informed consent prior to enrollment. All animal experiments were approved by the Institutional Animal Care and Use Committee of Kunming Medical University (Approval No. #kmmu20241594) and performed in compliance with the Guide for the Care and Use of Laboratory Animals.

## Conflicts of Interest

The authors declare no conflict of interest.

## Supporting information




**Supporting File 1**: advs73753‐sup‐0001‐Figures.docx.

**Supporting File 2**: advs73753‐sup‐0002‐Tables.docx.

**Supporting File 3**: advs73753‐sup‐0003‐TableS1.xlsx.

**Supporting File 4**: advs73753‐sup‐0004‐TableS3.xlsx.
[Correction added on 5th February, after first online publication: Supplementary File 1 and 2 are updated.]

## Data Availability

The data that support the findings of this study are available from the corresponding author upon reasonable request.

## References

[1] J. Wen , X. Zhang , C. C. Wong , et al., “Targeting Squalene Epoxidase Restores Anti‐PD‐1 Efficacy In Metabolic Dysfunction‐Associated Steatohepatitis‐Induced Hepatocellular Carcinoma,” Gut 73, no. 12 (2024): 2023–2036, 10.1136/gutjnl-2023-331117. 38744443 PMC11671884

[2] M. Xu , J. Zhao , L. Zhu , et al., “Targeting PYK_2_ With Heterobifunctional T6BP Helps Mitigate MASLD and MASH‐HCC Progression,” Journal of Hepatology 82, no. 2 (2025): 277–300, 10.1016/j.jhep.2024.08.029. 39260704

[3] J. M. Llovet , C. E. Willoughby , A. G. Singal , et al., “Nonalcoholic Steatohepatitis‐Related Hepatocellular Carcinoma: Pathogenesis And Treatment,” Nature Reviews Gastroenterology & Hepatology 20, no. 8 (2023): 487–503, 10.1038/s41575-023-00754-7. 36932227 PMC12165718

[4] Y. Ju , K. Xu , X. Chen , T. Wu , and Y. Yuan , “Metabolic‐Immune Microenvironment Crosstalk Mediating ICI Resistance in MASH‐HCC,” Trends in Endocrinology and Metabolism (2025), 10.1016/j.tem.2025.06.008. 40695685

[5] D. Pfister , N. G. Núñez , R. Pinyol , et al., “NASH Limits Anti‐Tumour Surveillance In Immunotherapy‐Treated HCC,” Nature 592, no. 7854 (2021): 450–456, 10.1038/s41586-021-03362-0. 33762733 PMC8046670

[6] X. Wang , L. Zhang , and B. Dong , “Molecular Mechanisms in MASLD/MASH‐Related HCC,” Hepatology 82, no. 5 (2024): 1303–1324, 10.1097/hep.0000000000000786. 38349726 PMC11323288

[7] Y. Liu , F. Wang , G. Yan , et al., “CPT1A Loss Disrupts BCAA Metabolism to Confer Therapeutic Vulnerability in TP53‐Mutated Liver Cancer,” Cancer Letters 595 (2024): 217006, 10.1016/j.canlet.2024.217006. 38823763

[8] T. Yang , N. Liang , J. Zhang , et al., “OCTN2 Enhances PGC‐1α‐Mediated Fatty Acid Oxidation and OXPHOS To Support Stemness In Hepatocellular Carcinoma,” Metabolism 147 (2023): 155628, 10.1016/j.metabol.2023.155628. 37315888

[9] K. Xu , T. Wu , X. Li , et al., “ADH1C Maintains The Homeostasis Of Metabolic Microenvironment To Inhibit Steatotic Hepatocellular Carcinoma,” Metabolism 168 (2025): 156267, 10.1016/j.metabol.2025.156267. 40233847

[10] F. González‐Romero , D. Mestre , I. Aurrekoetxea , et al., “E2F1 and E2F2‐Mediated Repression of CPT2 Establishes a Lipid‐Rich Tumor‐Promoting Environment,” Cancer Research 81, no. 11 (2021): 2874–2887, 10.1158/0008-5472.Can-20-2052. 33771899

[11] D. Huang , T. Li , X. Li , et al., “HIF‐1‐Mediated Suppression of Acyl‐CoA Dehydrogenases and Fatty Acid Oxidation Is Critical for Cancer Progression,” Cell Reports 8, no. 6 (2014): 1930–1942, 10.1016/j.celrep.2014.08.028. 25242319

[12] P. Fernández‐Tussy , M. P. Cardelo , H. Zhang , et al., “miR‐33 Deletion In Hepatocytes Attenuates MASLD‐MASH‐HCC Progression,” JCI Insight 9, no. 19 (2024): 168476, 10.1172/jci.insight.168476. PMC11466198 39190492

[13] N. Fujiwara , H. Nakagawa , K. Enooku , et al., “CPT2 Downregulation Adapts HCC To Lipid‐Rich Environment And Promotes Carcinogenesis Via Acylcarnitine Accumulation In Obesity,” Gut 67, no. 8 (2018): 1493–1504, 10.1136/gutjnl-2017-315193. 29437870 PMC6039238

[14] L. T. Izzo , S. Trefely , C. Demetriadou , et al., “Acetylcarnitine Shuttling Links Mitochondrial Metabolism To Histone Acetylation And Lipogenesis,” Science Advances 9, no. 18 (2023): adf0115, 10.1126/sciadv.adf0115. PMC10156126 37134161

[15] W. He , Q. Li , and X. Li , “Acetyl‐CoA Regulates Lipid Metabolism And Histone Acetylation Modification In Cancer,” Biochimica et Biophysica Acta (BBA)—Reviews on Cancer 1878, no. 1 (2023): 188837, 10.1016/j.bbcan.2022.188837. 36403921

[16] M. Li , L. Wang , L. Cong , et al., “Spatial Proteomics Of Immune Microenvironment In Nonalcoholic Steatohepatitis‐Associated Hepatocellular Carcinoma,” Hepatology 79, no. 3 (2024): 560–574, 10.1097/hep.0000000000000591. 37733002 PMC10871559

[17] S. J. G. Knottnerus , J. C. Bleeker , R. C. I. Wüst , et al., “Disorders Of Mitochondrial Long‐Chain Fatty Acid Oxidation And The Carnitine Shuttle,” Reviews in Endocrine and Metabolic Disorders 19, no. 1 (2018): 93–106, 10.1007/s11154-018-9448-1. 29926323 PMC6208583

[18] M. Dambrova , M. Makrecka‐Kuka , J. Kuka , et al., “Acylcarnitines: Nomenclature, Biomarkers, Therapeutic Potential, Drug Targets, and Clinical Trials,” Pharmacological Reviews 74, no. 3 (2022): 506–551, 10.1124/pharmrev.121.000408. 35710135

[19] C. Ge , J. Tan , X. Dai , et al., “Hepatocyte Phosphatase DUSP22 Mitigates NASH‐HCC Progression by Targeting FAK,” Nature Communications 13, no. 1 (2022): 5945, 10.1038/s41467-022-33493-5. PMC9547917 36209205

[20] J. Chen , T. K. Singh , S. Al Nemri , M. Zaidi , K. L. Billingsley , and J. M. Park , “Hyperpolarized [1‐ 13 C]Acetyl‐ l ‐Carnitine Probes Tricarboxylic Acid Cycle Activity In Vivo,” ACS Sensors 8, no. 8 (2023): 2927–2932, 10.1021/acssensors.3c01046. 37578472 PMC11227661

[21] L. Yuan , H. Jiang , Y. Jia , et al., “Fatty Acid Oxidation Supports Lymph Node Metastasis of Cervical Cancer via Acetyl‐CoA‐Mediated Stemness,” Advanced Science 11, no. 21 (2024): 2308422, 10.1002/advs.202308422. 38520724 PMC11151054

[22] L. Chen , S. Liu , and Y. Tao , “Regulating Tumor Suppressor Genes: Post‐Translational Modifications,” Signal Transduction and Targeted Therapy 5, no. 1 (2020): 90, 10.1038/s41392-020-0196-9. 32532965 PMC7293209

[23] M. Kabir , X. Hu , T. C. Martin , et al., “Harnessing the TAF1 Acetyltransferase for Targeted Acetylation of the Tumor Suppressor p53,” Advanced Science 12, no. 7 (2025): 2413377, 10.1002/advs.202413377. 39716936 PMC11831463

[24] D. Xu , W. Qian , Z. Yang , et al., “Acetylation Halts Missense Mutant p53 Aggregation And Rescues Tumor Suppression In Non‐Small Cell Lung Cancers,” Iscience 26, no. 7 (2023): 107003, 10.1016/j.isci.2023.107003. 37534137 PMC10391690

[25] R. E. Perez , C. D. Knights , G. Sahu , et al., “Restoration of DNA‐Binding And Growth‐Suppressive Activity Of Mutant Forms of p53 via a PCAF‐Mediated Acetylation Pathway,” Journal of Cellular Physiology 225, no. 2 (2010): 394–405, 10.1002/jcp.22285. 20589832 PMC3614009

[26] A. E. Knowell , D. Patel , D. J. Morton , P. Sharma , S. Glymph , and J. Chaudhary , “Id4 Dependent Acetylation Restores Mutant‐p53 Transcriptional Activity,” Molecular Cancer 12 (2013): 161, 10.1186/1476-4598-12-161. 24330748 PMC3866570

[27] D. J. Morton , D. Patel , J. Joshi , A. Hunt , A. E. Knowell , and J. Chaudhary , “ID4 Regulates Transcriptional Activity of Wild Type and Mutant p53 via K373 Acetylation,” Oncotarget 8, no. 2 (2017): 2536–2549, 10.18632/oncotarget.13701. 27911860 PMC5356822

[28] X. H. Li , D. Li , C. Liu , M. M. Zhang , X. J. Guan , and Y. P. Fu , “p33ING1b Regulates Acetylation of p53 in Oral Squamous Cell Carcinoma via SIR2,” Cancer Cell International 20 (2020): 398, 10.1186/s12935-020-01489-0. 32831651 PMC7436958

[29] L. R. Kong , R. W. Ong , T. Z. Tan , et al., “Targeting Codon 158 p53‐Mutant Cancers Via The Induction Of p53 Acetylation,” Nature Communications 11, no. 1 (2020): 2086, 10.1038/s41467-020-15608-y. PMC7190866 32350249

[30] M. Shvedunova and A. Akhtar , “Modulation Of Cellular Processes By Histone And Non‐Histone Protein Acetylation,” Nature Reviews Molecular Cell Biology 23, no. 5 (2022): 329–349, 10.1038/s41580-021-00441-y. 35042977

[31] A. N. H. Khan , C. J. Gregorie , and T. B. Tomasi , “Histone Deacetylase Inhibitors Induce TAP, LMP, Tapasin Genes and MHC Class I Antigen Presentation By Melanoma Cells,” Cancer Immunology, Immunotherapy 57, no. 5 (2008): 647–654, 10.1007/s00262-007-0402-4. 18046553 PMC3146348

[32] I. Pellicciotta , X. Cortez‐Gonzalez , R. Sasik , et al., “Presentation of Telomerase Reverse Transcriptase, a Self‐Tumor Antigen, is Down‐Regulated by Histone Deacetylase Inhibition,” Cancer Research 68, no. 19 (2008): 8085–8093, 10.1158/0008-5472.CAN-08-1014. 18829567 PMC11344586

[33] B. C. Taylor and J. M. Balko , “Mechanisms of MHC‐I Downregulation and Role in Immunotherapy Response,” Frontiers in Immunology 13 (2022): 844866, 10.3389/fimmu.2022.844866. 35296095 PMC8920040

[34] N. Pishesha , T. J. Harmand , and H. L. Ploegh , “A Guide To Antigen Processing And Presentation,” Nature Reviews Immunology 22, no. 12 (2022): 751–764, 10.1038/s41577-022-00707-2. 35418563

[35] S. P. Kolapalli , R. Sahu , N. R. Chauhan , et al., “RNA‐Binding RING E3‐Ligase DZIP3/hRUL138 Stabilizes Cyclin D1 to Drive Cell‐Cycle and Cancer Progression,” Cancer Research 81, no. 2 (2021): 315–331, 10.1158/0008-5472.CAN-20-1871. 33067265 PMC7116596

[36] J. Ferrer and N. Dimitrova , “Transcription Regulation By Long Non‐Coding RNAs: Mechanisms And Disease Relevance,” Nature Reviews Molecular Cell Biology 25, no. 5 (2024): 396–415, 10.1038/s41580-023-00694-9. 38242953 PMC11045326

[37] J. H. Yoon , K. Abdelmohsen , J. Kim , et al., “Scaffold Function of Long Non‐Coding RNA HOTAIR In Protein Ubiquitination,” Nature Communications 4 (2013): 2939, 10.1038/ncomms3939. PMC4556280 24326307

[38] L. Wang , L. Zhu , C. Liang , et al., “Targeting N6‐Methyladenosine Reader YTHDF1 With siRNA Boosts Antitumor Immunity in NASH‐HCC by Inhibiting EZH2‐IL‐6 Axis,” Journal of Hepatology 79, no. 5 (2023): 1185–1200, 10.1016/j.jhep.2023.06.021. 37459919

[39] M. Frigeni , B. Balakrishnan , X. Yin , et al., “Functional And Molecular Studies In Primary Carnitine Deficiency,” Human Mutation 38, no. 12 (2017): 1684–1699, 10.1002/humu.23315. 28841266 PMC5665702

[40] D. Q. Huang , A. G. Singal , Y. Kono , D. J. H. Tan , H. B. El‐Serag , and R. Loomba , “Changing Global Epidemiology of Liver Cancer From 2010 to 2019: NASH Is The Fastest Growing Cause Of Liver Cancer,” Cell Metabolism 34, no. 7 (2022): 969–977, 10.1016/j.cmet.2022.05.003. 35793659 PMC9762323

[41] M. Peiseler , R. Schwabe , J. Hampe , P. Kubes , M. Heikenwälder , and F. Tacke , “Immune Mechanisms Linking Metabolic Injury To Inflammation And Fibrosis In Fatty Liver Disease—Novel Insights Into Cellular Communication Circuits,” Journal of Hepatology 77, no. 4 (2022): 1136–1160, 10.1016/j.jhep.2022.06.012. 35750137

[42] B. Paul , M. Lewinska , and J. B. Andersen , “Lipid Alterations In Chronic Liver Disease And Liver Cancer,” JHEP Reports 4, no. 6 (2022): 100479, 10.1016/j.jhepr.2022.100479. 35469167 PMC9034302

[43] Y. Tang , Z. Chen , Q. Zuo , and Y. Kang , “Regulation of CD8+ T Cells By Lipid Metabolism In Cancer Progression,” Cellular & Molecular Immunology 21, no. 11 (2024): 1215–1230, 10.1038/s41423-024-01224-z. 39402302 PMC11527989

[44] P. Burda , A. Hlavackova , V. Polivkova , et al., “Imatinib Therapy Of Chronic Myeloid Leukemia Significantly Reduces Carnitine Cell Intake, Resulting In Adverse Events,” Molecular Metabolism 88 (2024): 102016, 10.1016/j.molmet.2024.102016. 39182842 PMC11403060

[45] F. Kopp and J. T. Mendell , “Functional Classification and Experimental Dissection of Long Noncoding RNAs,” Cell 172, no. 3 (2018): 393–407, 10.1016/j.cell.2018.01.011. 29373828 PMC5978744

[46] H. Wang , Y. Wang , S. Lai , et al., “LINC01468 Drives NAFLD‐HCC Progression Through CUL4A‐Linked Degradation of SHIP2,” Cell Death Discovery 8, no. 1 (2022): 449, 10.1038/s41420-022-01234-8. 36344496 PMC9640567

[47] Y. Chi , Z. Gong , H. Xin , Z. Wang , and Z. Liu , “Long Noncoding RNA lncARSR Promotes Nonalcoholic Fatty Liver Disease and Hepatocellular Carcinoma by Promoting YAP1 and Activating the IRS2/AKT Pathway,” Journal of Translational Medicine 18, no. 1 (2020): 126, 10.1186/s12967-020-02225-y. 32169080 PMC7071718

[48] Z. Lin , Z. Huang , J. Qiu , et al., “m6A‐Mediated lnc‐OXAR Promotes Oxaliplatin Resistance by Enhancing Ku70 Stability In Non‐Alcoholic Steatohepatitis‐Related Hepatocellular Carcinoma,” Journal of Experimental & Clinical Cancer Research 43, no. 1 (2024): 206, 10.1186/s13046-024-03134-4. 39054531 PMC11271202

[49] S. T. Crooke , J. L. Witztum , C. F. Bennett , and B. F. Baker , “RNA‐Targeted Therapeutics,” Cell Metabolism 27, no. 4 (2018): 714–739, 10.1016/j.cmet.2018.03.004. 29617640

[50] M. E. Rinella , J. V. Lazarus , V. Ratziu , et al., “A Multisociety Delphi Consensus Statement On New Fatty Liver Disease Nomenclature,” Journal of Hepatology 79, no. 6 (2023): 1542–1556, 10.1016/j.jhep.2023.06.003. 37364790

[51] C. Eipel , H. Schuett , C. Glawe , R. Bordel , M. D. Menger , and B. Vollmar , “Pifithrin‐Alpha Induced p53 Inhibition Does Not Affect Liver Regeneration After Partial Hepatectomy In Mice,” Journal of Hepatology 43, no. 5 (2005): 829–835, 10.1016/j.jhep.2005.04.018. 16087272

