# Supplementary material for: OCTN2 Activates a Non‐Canonical Carnitine Metabolic Pathway to Promote MASH‐HCC Progression and Immunotherapy Resistance
Source: Adv Sci (Weinh). 2026 Jan 21;13(16):e17054. doi: 10.1002/advs.202517054 (PMC13042636; doi:10.1002/advs.202517054)
Supplement: Supplementary file 1 — Supporting File 1: advs73753‐sup‐0001‐Figures.docx. [file ADVS-13-e17054-s001.docx]

**Supplementary Figures and Legends**


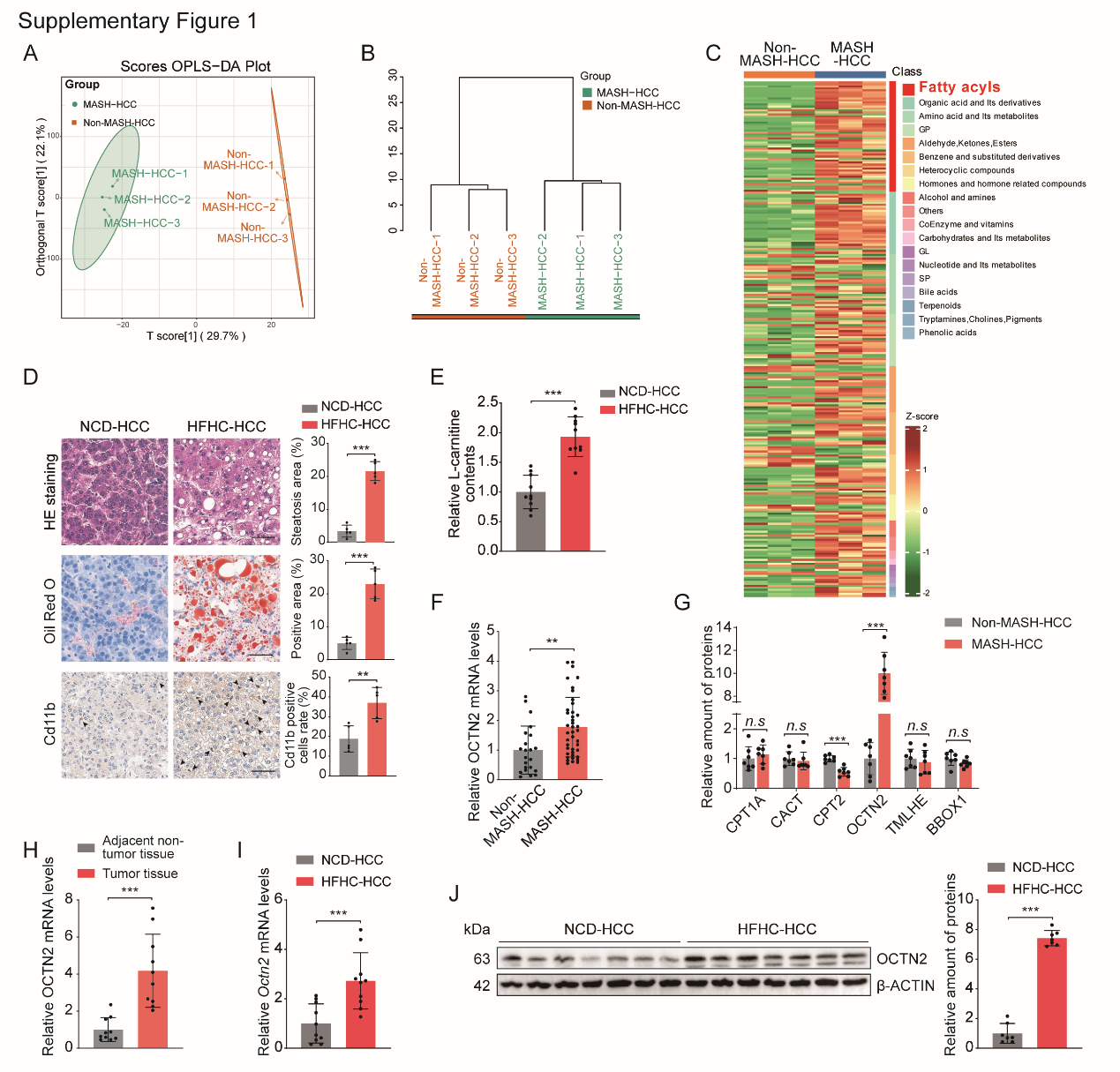


**Supplementary Figure 1. A.** Score plot from orthogonal partial least squares-discriminant analysis (OPLS-DA) showing separation between MASH-HCC and non-MASH-HCC samples based on metabolomics data (n = 3 per group). **B.** Hierarchical clustering analysis of metabolite profiles in MASH-HCC and non-MASH-HCC samples (n = 3 per group). **C.** Heatmap depicting the differential abundance of metabolites between MASH-HCC and non-MASH-HCC samples, with metabolites categorized by their chemical class (n = 3 per group). **D.** Representative H&E, Oil Red O, and Cd11b staining images with corresponding quantitative analyses of tumor tissues from HCC mice fed a normal chow diet (NCD) or a high-fat/high-cholesterol (HFHC) diet (n = 5 per group). Scale bars, 50 μm. **E.** L-carnitine levels in liver tumor tissues from HFHC-HCC and NCD-HCC mice (n = 10 per group). **F.** qRT-PCR analysis of OCTN2 mRNA expression in expanded clinical samples from patients with MASH-HCC (n = 41) and non-MASH-HCC (n = 22). **G.** Relative level of carnitine metabolism-related proteins in MASH-HCC and non-MASH-HCC tissues (n = 7 per group). **H.** qRT-PCR analysis of OCTN2 mRNA expression in paired tumor and adjacent non-tumor tissues from MASH-HCC patients (n = 10 per group). **I.** qRT-PCR analysis of OCTN2 mRNA expression in liver tumors from NCD-HCC and HFHC-HCC mice (n = 10 per group). **J.** Western blot analysis of OCTN2 protein expression in liver tumor tissues from HFHC-HCC and NCD-HCC mice (n = 7 per group). The data are expressed as the mean ± SD. P-values were determined by two-tailed Student’s t-test (Figure S1 D, E, F, G, H, I, J). Statistical significance: *n.s* means not significant, *p < 0.05, **p < 0.01, ***p < 0.001.


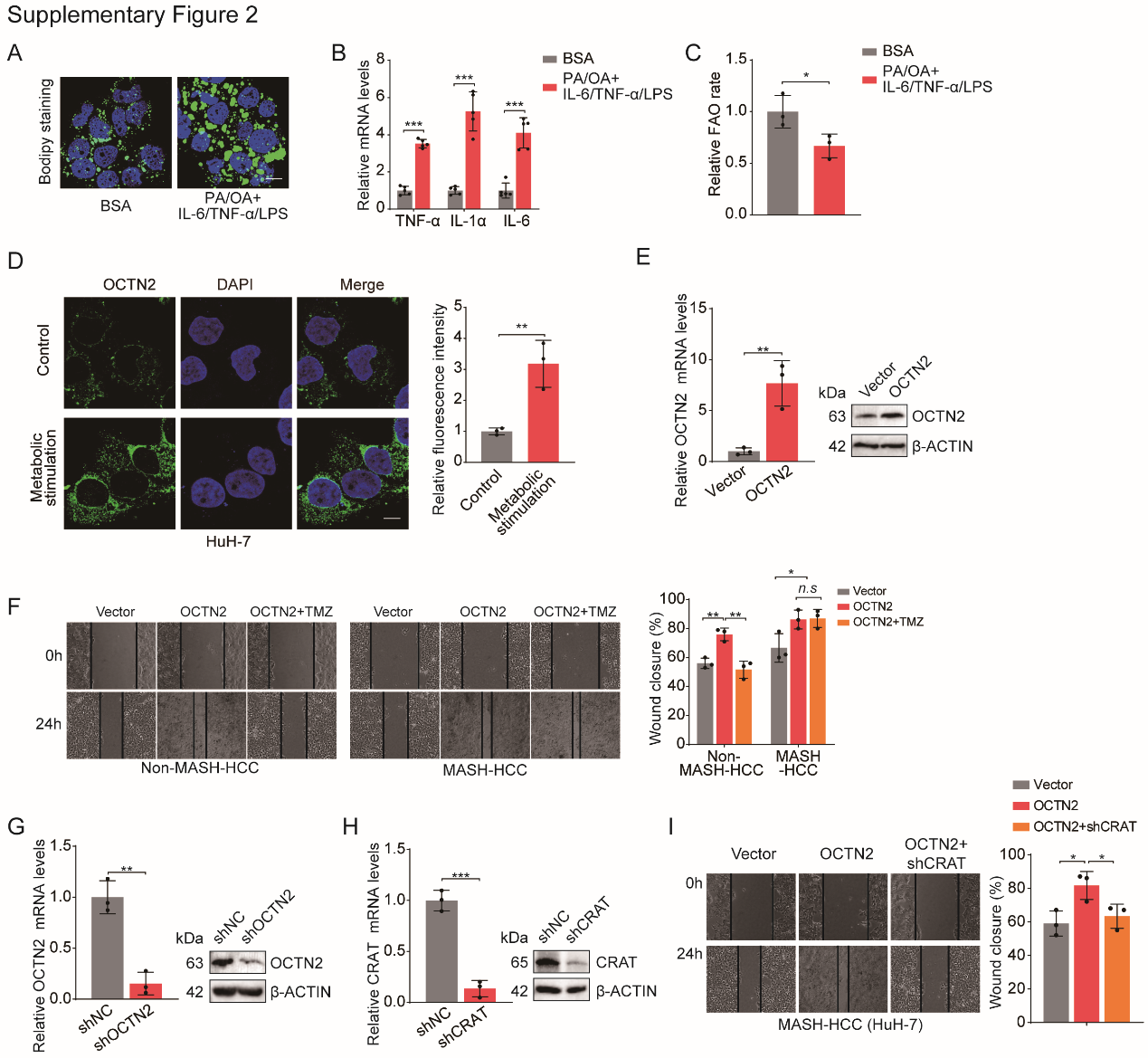


**Supplementary figure 2.** **A.** Bodipy staining showing lipid droplet accumulation in HuH-7 cells treated with BSA or PA/OA combined with IL-6, TNF-α, and LPS (n = 5 per group). Scale bars, 25 μm. **B.** qRT-PCR analysis of inflammatory gene expression (TNF-α, IL-1α, IL-6) under the same treatment conditions as in A (n = 5 per group). **C.** Fatty acid oxidation (FAO) capacity of HuH-7 cells treated as in A (n = 3 per group). **D.** Immunofluorescence staining showing elevated OCTN2 expression in MASH-HCC cells compared to non-MASH-HCC cells (n = 3 per group). Scale bars, 25 μm. **E.** Quantification of relative OCTN2 mRNA and protein levels in MASH-HCC cells transfected with control vector or OCTN2 overexpression plasmids (n = 3 per group). **F.** Wound healing assays evaluating the migration ability of MASH-HCC and non-MASH-HCC cells transfected with vector or OCTN2, with or without trimetazidine (TMZ, 1 mM) treatment (n = 3 per group). **G.** Relative OCTN2 mRNA and protein levels in MASH-HCC cells transfected with shOCTN2 or shNC (n = 3 per group). **H.** Relative CRAT mRNA and protein levels in MASH-HCC cells transfected with shCRAT or shNC (n = 3 per group). **I.** Wound healing assays evaluating migration ability in MASH-HCC cells transfected with vector, OCTN2, or OCTN2 + shCRAT (n = 3 per group). The data are expressed as the mean ± SD. P-values were determined by two-tailed Student’s t-test (Figure S2 B, C, D, E, G, H) or one-way ANOVA followed by a post hoc Tukey test (Figure S2 F, I). Statistical significance: *n.s* means not significant, *p < 0.05, **p < 0.01, ***p < 0.001.


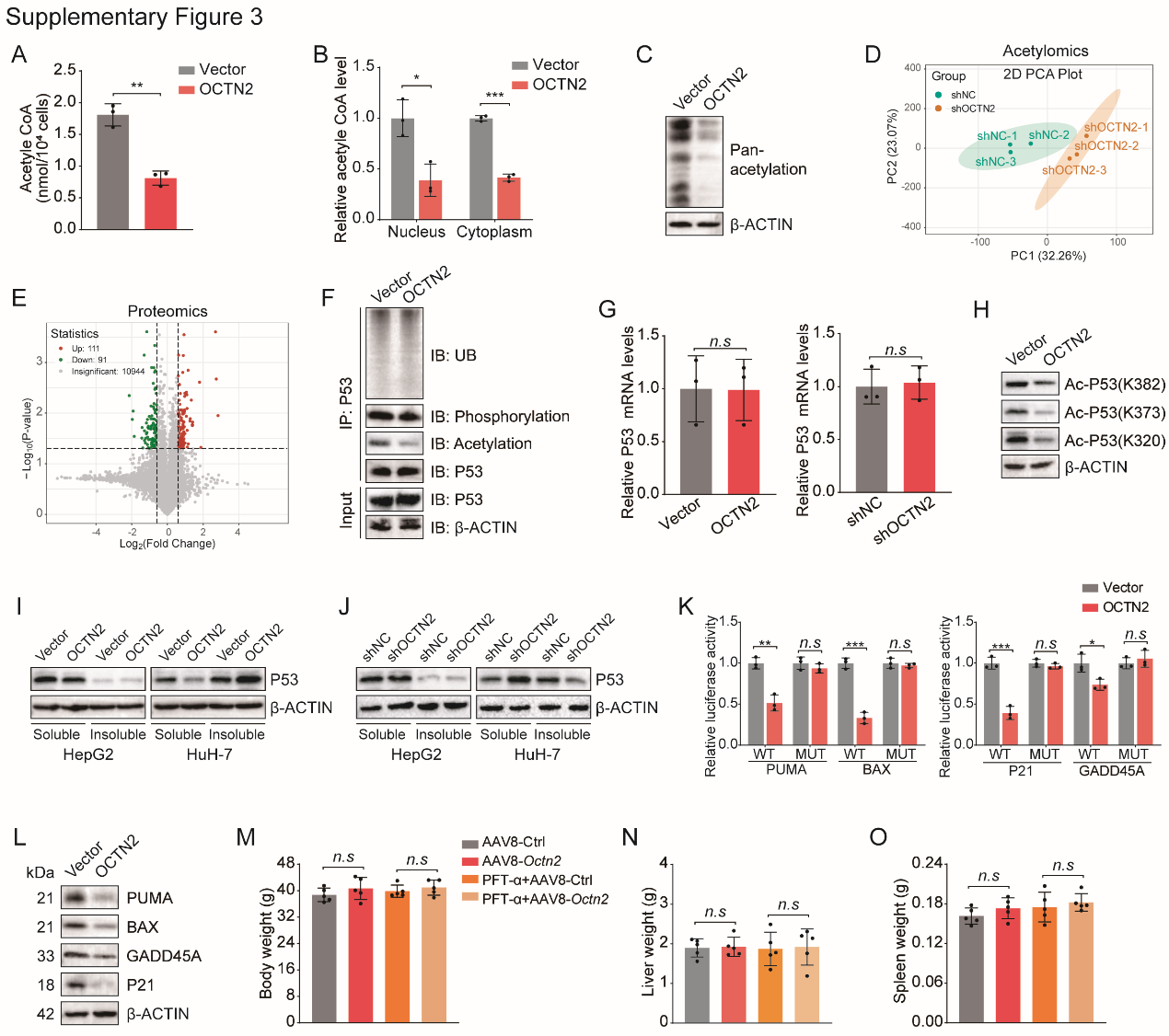


**Supplementary Figure 3. A.** Quantification of intracellular acetyl-CoA levels in MASH-HCC cells transfected with vector or OCTN2 plasmids (n = 3 per group). **B.** Relative acetyl-CoA levels in nuclear and cytoplasmic fractions of MASH-HCC cells transfected with vector or OCTN2 plasmids (n = 3 per group). **C.** Western blot analysis of pan-acetylation in MASH-HCC cells transfected with vector or OCTN2 plasmids (n = 3 per group). **D.** Two-dimensional principal component analysis (PCA) plot showing clear separation between MASH-HCC cells transfected with shNC or shOCTN2 plasmids, based on acetylomics data (n = 3 per group). **E.** Volcano plot of proteomics data showing differentially expressed proteins in MASH-HCC cells transfected with shNC or shOCTN2 plasmids (n = 3 per group). **F.** Western blot analysis of total protein, and acetylated, phosphorylated, ubiquitinated p53 in MASH-HCC cells transfected with vector or OCTN2 plasmids (n = 3 per group). **G.** qRT-PCR analysis of p53 mRNA levels in MASH-HCC cells transfected with OCTN2 or shOCTN2 plasmids (n = 3 per group). **H.** Western blot analysis of p53 acetylation at K320, K373, and K382 in MASH-HCC cells transfected with vector or OCTN2 plasmids (n = 3 per group). **I.** Western blot analysis of detergent-soluble and detergent-insoluble fractions from HepG2 (wild-type p53) and Huh7 (p53-Y220C mutant) cells transfected with vector or OCTN2 expression plasmids (n = 3 per group). **J.** Western blot analysis of detergent-soluble and detergent-insoluble fractions from HepG2 (wild-type p53) and Huh7 (p53-Y220C mutant) cells transfected with shNC or shOCTN2 plasmids (n = 3 per group). **K.** Dual-luciferase reporter assays evaluating transcriptional activity of p53 target genes (P21, GADD45A, BAX, PUMA) in MASH-HCC cells transfected with vector or OCTN2 plasmids (n = 3 per group). **L.** Western blot analysis of p53 downstream target proteins (P21, GADD45A, BAX, PUMA) in MASH-HCC cells transfected with vector or OCTN2 plasmids (n = 3 per group). **M–O.** Measurements of body weight, liver weight, and spleen weight in HFHC-HCC mice injected with AAV8-TBG-Ctrl or AAV8-TBG-*Octn2*, with or without PFT-α treatment (n = 5 per group). The data are expressed as the mean ± SD. P-values were determined by two-tailed Student’s t-test (Figure S3 A, B, F, K) or one-way ANOVA followed by a post hoc Tukey test (Figure S3 M, N, O). Statistical significance: *n.s* means not significant, *p < 0.05, **p < 0.01, ***p < 0.001.


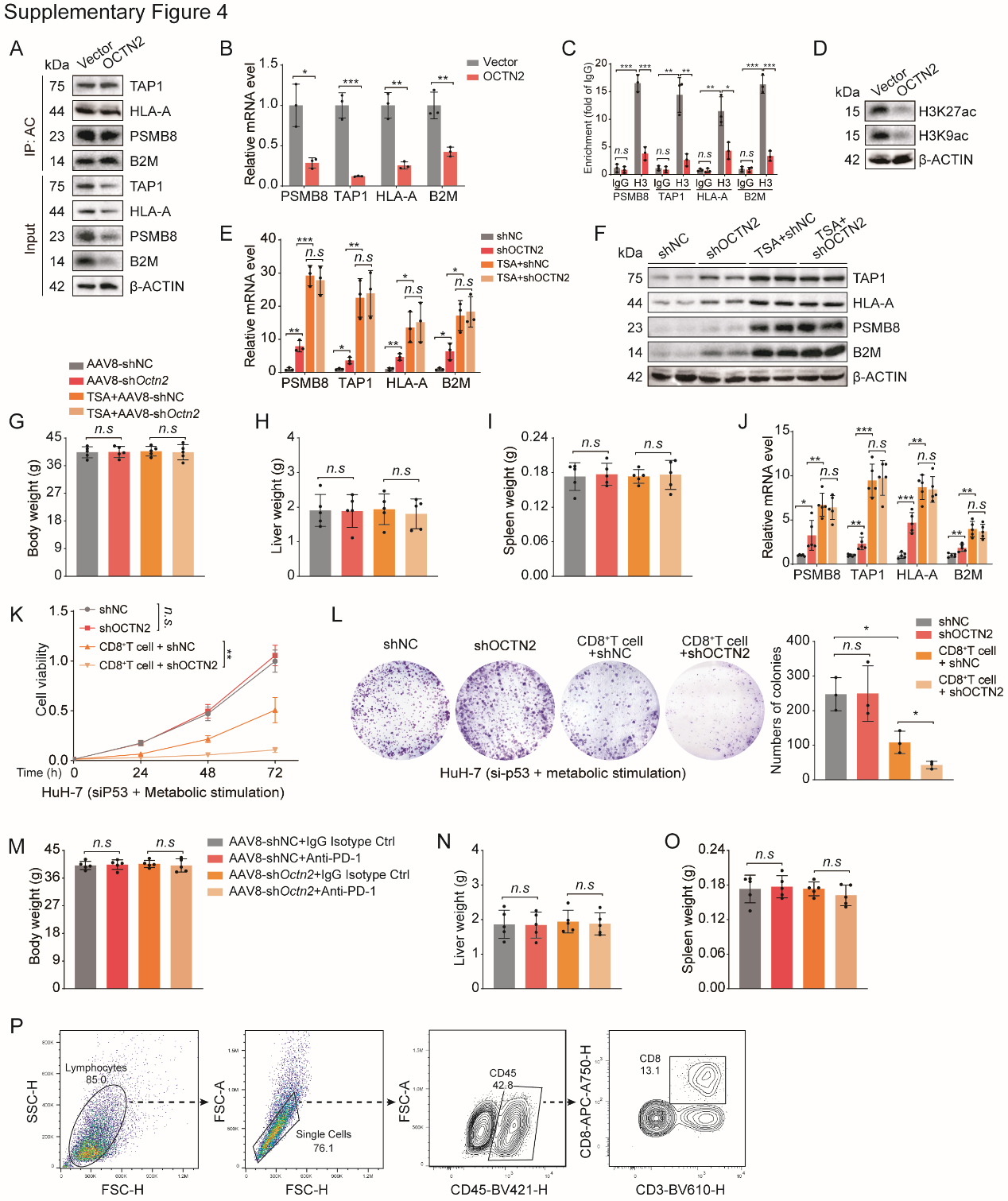


**Supplementary Figure 4. A.** Western blot analysis of MHC-I pathway–related proteins (PSMB8, TAP1, HLA-A, B2M) and their acetylation levels in MASH-HCC cells transfected with vector or OCTN2 plasmid (n = 3 per group). **B.** qRT-PCR analysis of MHC-I pathway gene expression in MASH-HCC cells transfected with vector or OCTN2 plasmid (n = 3 per group). **C.** ChIP-qPCR analysis of H3 occupancy at the promoters of MHC-I pathway genes in MASH-HCC cells transfected with vector or OCTN2 plasmid (n = 3 per group). **D.** Western blot analysis of H3K9ac and H3K27ac levels in MASH-HCC cells transfected with vector or OCTN2 plasmid (n = 3 per group). **E.** qRT-PCR analysis of MHC-I pathway gene mRNA expression in MASH-HCC cells transfected with shNC or shOCTN2, with or without trichostatin A (TSA) treatment (n = 3 per group). **F.** Western blot analysis of MHC-I pathway gene proteins in MASH-HCC cells transfected with shNC or shOCTN2, with or without TSA treatment (n = 3 per group). **G–I.** Body weight, liver weight, and spleen weight measurements in MASH-HCC mice treated with AAV8-TBG-shNC, AAV8-TBG-sh*Octn2*, TSA + AAV8-TBG-shNC, or TSA + AAV8-TBG-sh*Octn2* (n = 5 per group). **J.** qRT-PCR analysis showing mRNA expression levels of MHC-I pathway genes in liver tumors from the same groups as in G (n = 5 per group). **K, L.** CCK-8 and colony formation assays showing the viability and proliferation of P53-knockdown MASH-HCC cells (si-P53), transfected with shNC or shOCTN2 and co-cultured with CD8⁺ T cells (n = 3 per group). **M–O.** Body weight, liver weight, and spleen weight measurements in MASH-HCC mice treated with AAV8-shNC + IgG isotype control, AAV8-shNC + anti–PD-1, AAV8-sh*Octn2* + IgG, or AAV8-sh*Octn2* + anti–PD-1 (n = 5 per group). **P.** Gating strategy for tumor-infiltrating immune cell populations in MASH-HCC tumor tissues from AAV8-shNC + IgG isotype control mice. The data are expressed as the mean ± SD. P-values were determined by two-tailed Student’s t-test (Figure S4 B, C), one-way ANOVA followed by a post hoc Tukey test (Figure S4 E, G, H, I, J, L, M, N, O) or two-way ANOVA with post hoc Tukey tests (Figure S4 K). Statistical significance: *n.s* means not significant, *p < 0.05, **p < 0.01, ***p < 0.001.

**
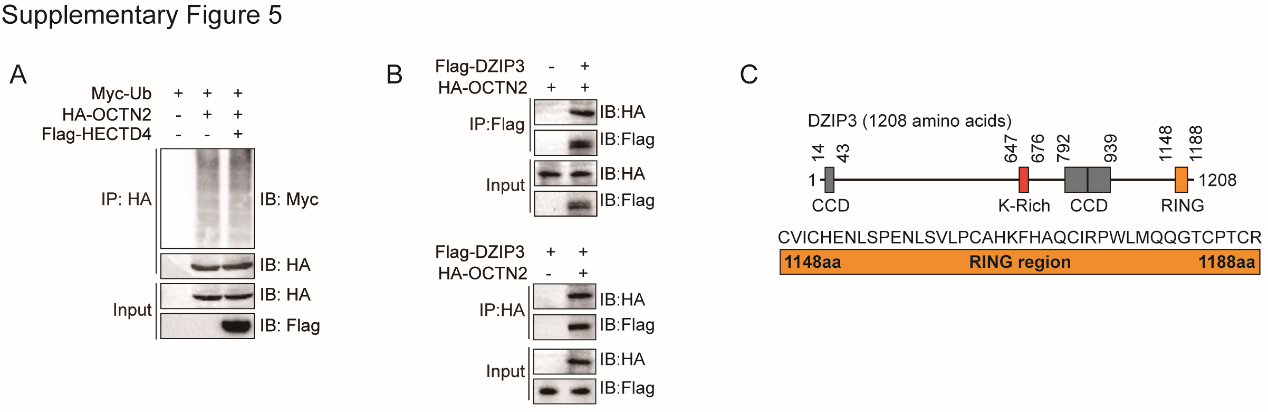
**

**Supplementary Figure 5. A.** Western blot analysis showing OCTN2 ubiquitination levels in MASH-HCC cells co-transfected with Flag-tagged HECTD4 (n = 3 per group). **B.** Exogenous co-IP analysis demonstrating the interaction between OCTN2 and DZIP3 in MASH-HCC cells (n = 3 per group). **C.** Schematic representation of DZIP3 protein domains and the amino acid sequence of the RING domain.

**
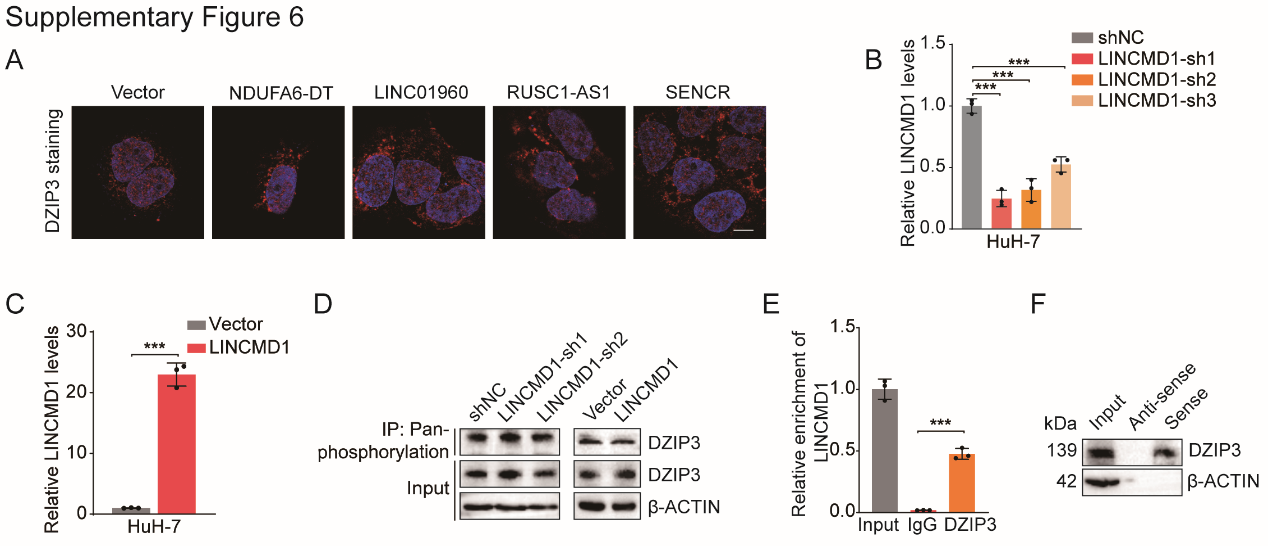
**

**Supplementary Figure 6. A.** Immunofluorescence staining showing the subcellular localization of DZIP3 following overexpression of MALT1, LINC01960, RUSC1-AS1, and AC092143.3 in HuH-7 cells (n = 3 per group). **B, C.** qRT-PCR analysis showing LINCMD1 expression levels in MASH-HCC cells following knockdown or overexpression (n = 3 per group). **D.** Western blot analysis showing total and phosphorylated DZIP3 protein levels in MASH-HCC cells following LINCMD1 overexpression or knockdown (n = 3 per group). **E, F.** RIP and RNA pull-down assays validating the direct interaction between LINCMD1 and DZIP3 in MASH-HCC cells (n = 3 per group). The data are expressed as the mean ± SD. P-values were determined by two-tailed Student’s t-test (Figure S6 C, E) or one-way ANOVA followed by a post hoc Tukey test (Figure S6 B). Statistical significance: *n.s* means not significant, *p < 0.05, **p < 0.01, ***p < 0.001.

**
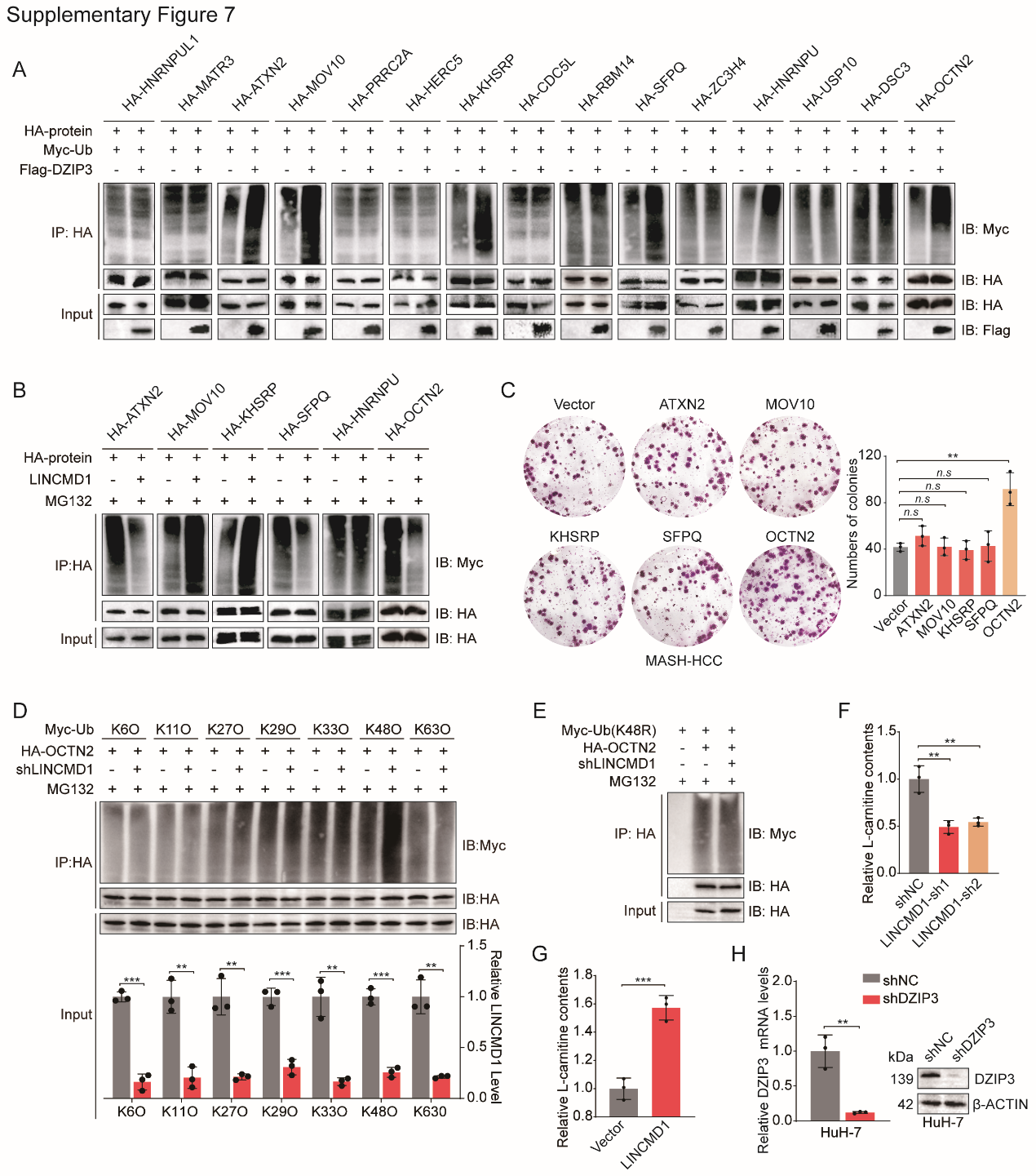
**

**Supplementary Figure 7. A.** Western blot analysis of the ubiquitination levels of potential DZIP3-interacting proteins in MASH-HCC cells transfected with vector or DZIP3 plasmids (n = 3 per group). **B.** Western blot analysis showing the ubiquitination levels of ATXN2, MOV10, KHSRP, SFPQ, HNRNPU, and OCTN2 in MASH-HCC cells transfected with vector or LINCMD1 plasmids (n = 3 per group). **C.** Colony formation assays and corresponding quantification showing the proliferative capacity of MASH-HCC cells transfected with vector, ATXN2, MOV10, KHSRP, SFPQ, or OCTN2 plasmids (n = 3 per group). **D.** Western blot analysis of the ubiquitination pattern of OCTN2 in MASH-HCC cells co-transfected with HA-tagged OCTN2, shLINCMD1, and Myc-tagged ubiquitin mutants (K6O, K11O, K27O, K29O, K33O, K48O, and K63O) (n = 3 per group). **E.** Western blot analysis showing K48-linked ubiquitination of OCTN2 in MASH-HCC cells co-transfected with HA-tagged OCTN2, K48-mutant Myc-tagged ubiquitin, and shLINCMD1 (n = 3 per group). **F, G.** Quantification of intracellular L-carnitine levels in MASH-HCC cells following LINCMD1 knockdown or overexpression (n = 3 per group). **H.** Establishment and validation of stable DZIP3-knockdown MASH-HCC cell lines (n = 3 per group). The data are expressed as the mean ± SD. P-values were determined by two-tailed Student’s t-test (Figure S7 D, G, H) or one-way ANOVA followed by a post hoc Tukey test (Figure S7 C, F). Statistical significance: *n.s* means not significant, *p < 0.05, **p < 0.01, ***p < 0.001.


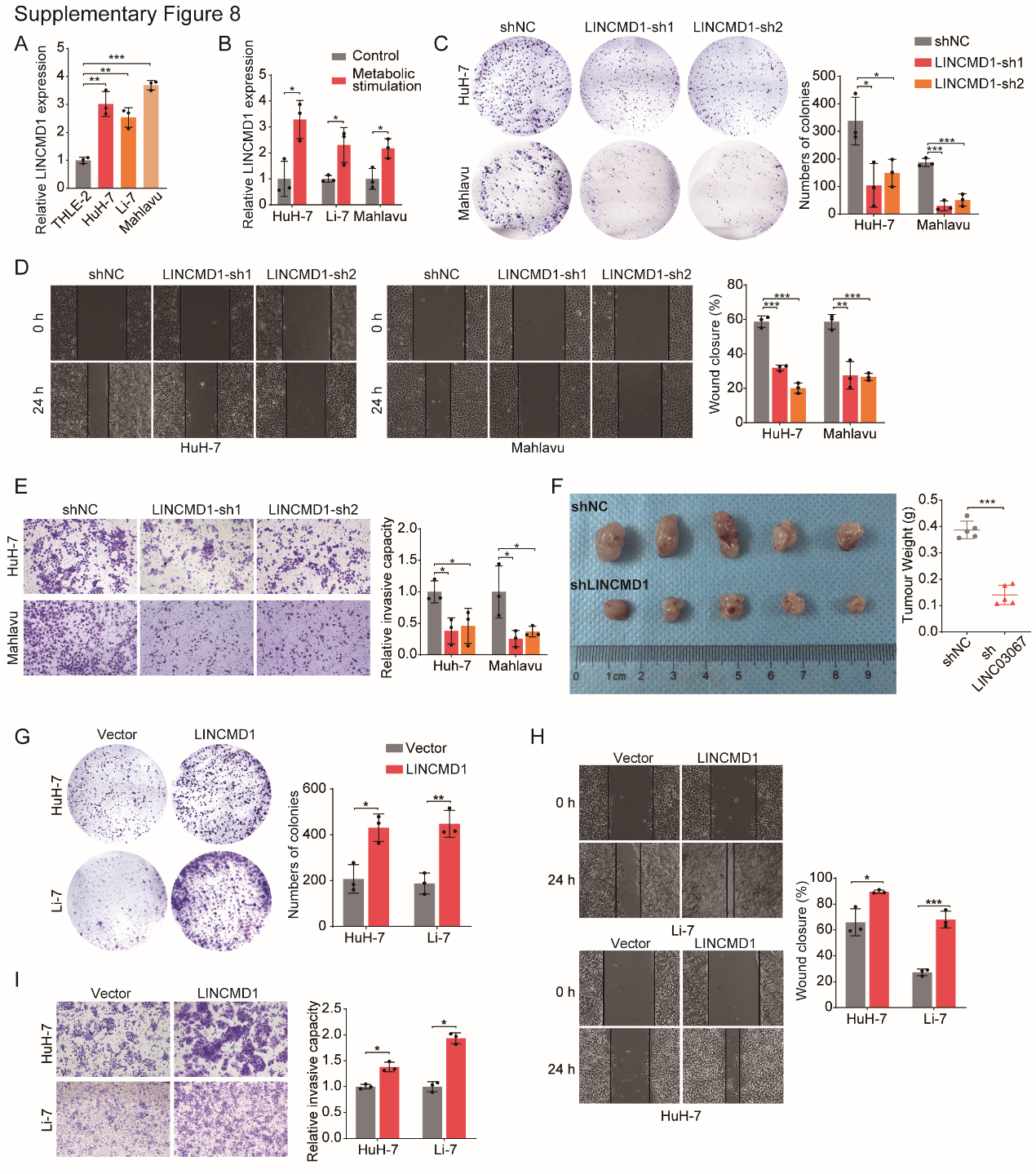


**Supplementary Figure 8. A.** qRT-PCR analysis of LINCMD1 expression in non-viral HCC cell lines (HuH-7, Li7, Mahlavu) compared to normal human hepatocyte cell line (THLE-2) (n = 3 per group). **B.** qRT-PCR analysis of LINCMD1 expression in non-viral HCC cell lines treated with control or metabolic stimulation (n = 3 per group). **C–E.** Colony formation assays, wound healing, and Transwell invasion assays demonstrating the proliferation, migratory, and invasive abilities of MASH-HCC cells with shNC, LINCMD1-sh1, or LINCMD1-sh2 vector (n = 3 per group). **F.** Representative images and quantification of tumor weight in subcutaneous CDX models established using HuH-7 cells transfected with shNC or shLINCMD1, in HFHC-fed BALB/c nude mice (n = 5 per group). **G–H.** Colony formation assays, wound healing, and Transwell invasion assays show the proliferation, migration, and invasion abilities of MASH-HCC cells transfected with LINCMD1 overexpression or empty vector (n = 3 per group). The data are expressed as the mean±SD. P-values were determined by two-tailed Student’s t-test or one-way ANOVA followed by a post hoc Tukey test. Statistical significance: n.s. means not significant, *p < 0.05, **p < 0.01, ***p < 0.001. The data are expressed as the mean ± SD. P-values were determined by two-tailed Student’s t-test (Figure S8 B, F, G, H, I) or one-way ANOVA followed by a post hoc Tukey test (Figure S8 A, C, D, E). Statistical significance: *n.s* means not significant, *p < 0.05, **p < 0.01, ***p < 0.001.


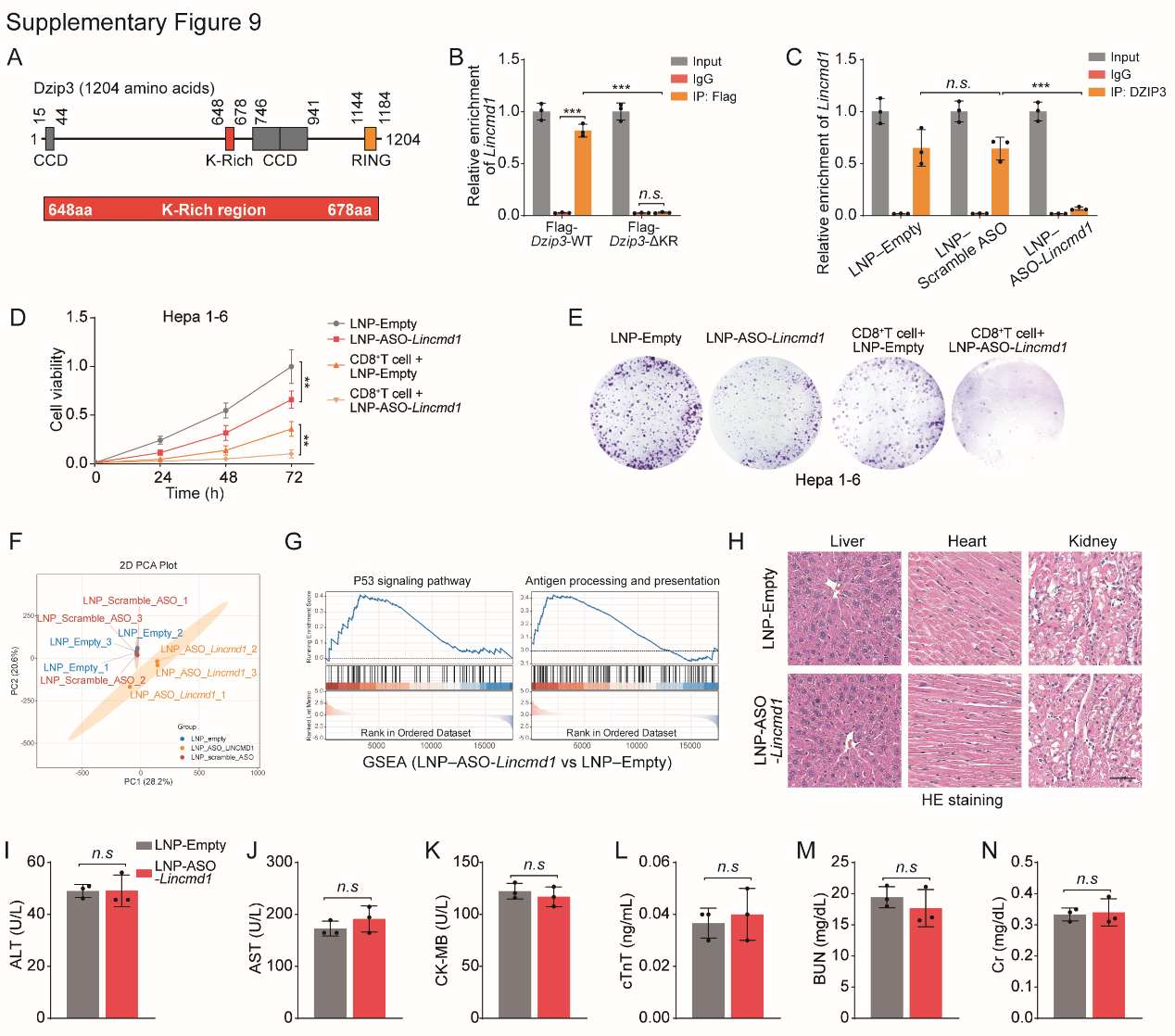


**Supplementary Figure 9. A.** Schematic illustration of *Dzip3* protein domains and amino acid sequence of the lysine-rich (KR) region. **B.** RIP assay showing the binding enrichment of Flag-*Dzip3*-WT to *Lincmd1* and Flag-*Dzip3*-ΔKR to *Lincmd1* (n = 3 per group). **C.** RIP assay showing the binding enrichment of *Dzip3* to *Lincmd1* after transfection with LNP-Empty, LNP-Scramble ASO, LNP-ASO-*Lincmd1* (n = 3 per group). **D, E.** CCK-8 and colony formation assays showing viability and proliferation of MASH-HCC cells co-cultured with CD8⁺ T cells under LNP-ASO-*Lincmd1* or control conditions (n = 3 per group). **F.** Two-dimensional principal component analysis (PCA) plot illustrating global transcriptomic variance among MASH-HCC mouse tumors treated with LNP-Empty, LNP-Scramble-ASO, or LNP-ASO-Lincmd1 (n = 3 per group). **G.** GSEA analysis showing activation of the p53 signaling pathway and the antigen processing and presentation pathway in MASH-HCC tumors treated with LNP-ASO-*Lincmd1* compared with those treated with LNP-Empty (n = 3 per group). **H.** Representative H&E staining images of liver, heart, and kidney tissues from mice treated with LNP-ASO-Lincmd1 or LNP-Empty (n = 3 per group). **I, J.** Serum alanine aminotransferase (ALT) and aspartate aminotransferase (AST) levels indicating hepatic toxicity after LNP-ASO-*Lincmd1* treatment in mice (n = 3 per group). **K, L.** Serum blood urea nitrogen (BUN) and creatinine (Cr) levels assessing renal toxicity after LNP-ASO-*Lincmd1* treatment (n = 3 per group). **M, N.** Serum creatine kinase-MB (CK-MB) and cardiac troponin T (cTnT) levels indicating cardiac toxicity after LNP-ASO-*Lincmd1* treatment (n = 3 per group). The data are expressed as the mean ± SD. P-values were determined by two-tailed Student’s t-test or one-way ANOVA followed by a post hoc Tukey test. Statistical significance: *n.s* means not significant, *p < 0.05, **p < 0.01, ***p < 0.001.


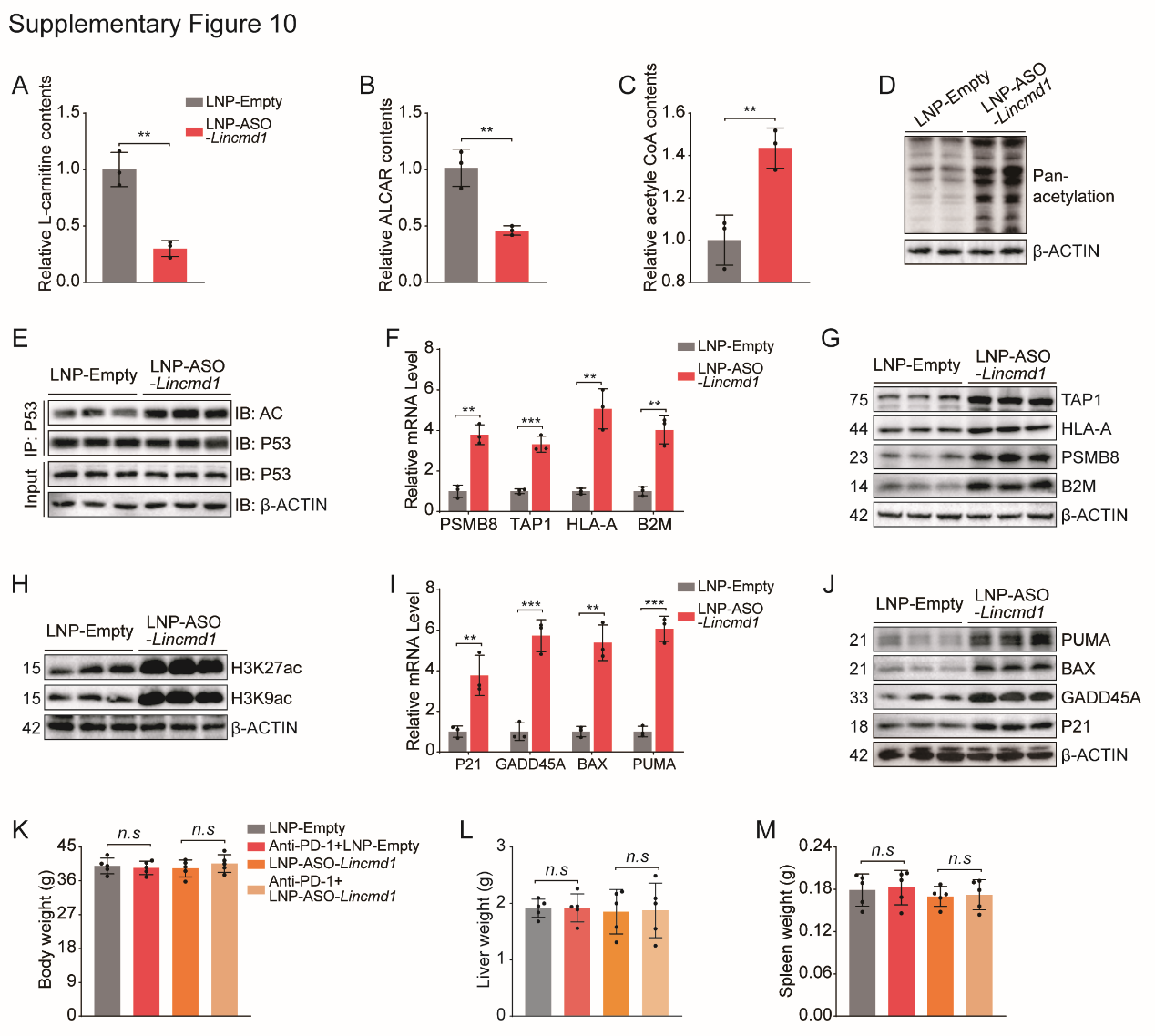


**Supplementary Figure 10. A-C.** Quantification of L-carnitine, ALCAR and acetyl-CoA levels in liver tumor tissues after LNP-ASO-*Lincmd1* treatment (n = 3 per group). **D.** Western blot analysis showing pan-acetylation levels in liver tumor tissues from mice treated with LNP-ASO-*Lincmd1* (n = 3 per group). **E.** Western blot analysis showing total and acetylated p53 protein levels in liver tumor tissues after LNP-ASO-*Lincmd1* treatment (n = 3 per group). **F, G.** qPCR and Western blot analysis showing mRNA and protein expression levels of p53 downstream target genes (P21, GADD45A, BAX, PUMA) in liver tumor tissues after LNP-ASO-*Lincmd1* treatment (n = 3 per group). **H.** Western blot analysis showing histone H3 acetylation levels in liver tumors from LNP-ASO-*Lincmd1* treated mice (n = 3 per group). **I, J.** qPCR and Western blot analysis showing mRNA and protein expression levels of MHC-I pathway–related genes (PSMB8, TAP1, HLA-A, B2M) in liver tumor tissues after LNP-ASO-*Lincmd1* treatment (n = 3 per group). **K-M.** Body weight, liver weight, and spleen weight measurements in MASH-HCC mice treated with LNP-Empty, anti–PD-1+LNP-*Ctrl*, LNP-ASO-*Lincmd1*, anti–PD-1+ LNP-ASO-*Lincmd1* (n = 5 per group). The data are expressed as the mean±SD. The data are expressed as the mean ± SD. P-values were determined by two-tailed Student’s t-test (Figure S10 A, B, C, F, I) or one-way ANOVA followed by a post hoc Tukey test (Figure S8 K, L, M). Statistical significance: *n.s* means not significant, *p < 0.05, **p < 0.01, ***p < 0.001.
