# Supplementary material for: OCTN2 Activates a Non‐Canonical Carnitine Metabolic Pathway to Promote MASH‐HCC Progression and Immunotherapy Resistance
Source: Adv Sci (Weinh). 2026 Jan 21;13(16):e17054. doi: 10.1002/advs.202517054 (PMC13042636; doi:10.1002/advs.202517054)
Supplement: Supplementary file 2 — Supporting File 2: advs73753‐sup‐0002‐Tables.docx. [file ADVS-13-e17054-s004.docx]

**Title**

**OCTN2 Activates a Non-Canonical Carnitine Metabolic Pathway to Promote MASH-HCC Progression and Immunotherapy Resistance**

**Supplementary Tables**

| **Supplementary Table 2. Clinical characteristics of HCC patients.** | | |
| --- | --- | --- |
| Clinical characteristics | Non-MASH-HCC  (n=22) | MASH-HCC  (n=41) |
| Age (year) | 52.1 ± 7.0 | 51.5 ± 7.4 |
| Sex, Male/Female | 15/7 | 25/16 |
| BMI (kg/m^2^) | 23.4 ± 1.2 | 27.7 ± 2.9 |
| Diabetes | 0 | 32 |
| Hypertension | 0 | 27 |
| Hepatitis B Positive | 19 | 0 |
| Hepatitis C Positive | 3 | 0 |
| Tumor size (cm) | 5.1 ± 0.6 | 4.9 ± 0.9 |
| Number of tumors (Solitary/Multiple) | 19/3 | 37/4 |
| Child-Pugh Score(A/B/C) | 14/8/0 | 32/9/0 |
| TNM Stage(I II/III IV) | 15/17 | 30/11 |
| NAS | ≤2 | ≥3 |
| Treatment |  |  |
| Surgery | 22 | 41 |
| Targeted Therapy | 0 | 0 |
| MASH Treatment | 0 | 0 |

HCC: hepatocellular carcinoma; MASH-HCC: metabolic dysfunction-associated steatohepatitis related HCC; SD: standard deviation; BMI: body mass index; NAS: nonalcoholic steatohepatitis activity score, TNM: tumor, node, metastasis classification. The descriptive statistics (age, BMI, and Tumor size) are present as Mean ± SD.

| **Supplementary Table 4. Univariate Cox analysis for overall survival** | | | | | | | | |
| --- | --- | --- | --- | --- | --- | --- | --- | --- |
| Characteristic | β | SE | Wald | df | P | HR | 95% CI | |
| Age | 0.094 | 0.033 | 8.156 | 1 | 0.004 | 1.098 | 1.03 | 1.171 |
| Gender | -0.102 | 0.373 | 0.075 | 1 | 0.784 | 0.903 | 0.435 | 1.876 |
| BMI | 0.043 | 0.075 | 0.336 | 1 | 0.562 | 1.044 | 0.902 | 1.209 |
| Stage | 1.377 | 0.267 | 26.594 | 1 | 0 | 3.965 | 2.349 | 6.693 |
| Diabete | 0.221 | 0.399 | 0.308 | 1 | 0.579 | 1.248 | 0.571 | 2.729 |
| Hypertension | 0.179 | 0.399 | 0.201 | 1 | 0.654 | 1.196 | 0.547 | 2.617 |
| OCTN2 | 1.825 | 0.378 | 23.351 | 1 | 0 | 6.203 | 2.959 | 13.003 |
| LINCMD1 | 2.334 | 0.557 | 17.526 | 1 | 0 | 10.314 | 3.459 | 30.755 |

β: coefficient; SE: standard error; Wald: Wald statistic; df: degrees of freedom; P: P-value; HR: hazard ratio; 95% CI: 95% confidence interval

| **Supplementary Table 5. Univariate Cox analysis for progression-free survival** | | | | | | | | |
| --- | --- | --- | --- | --- | --- | --- | --- | --- |
| Characteristic | β | SE | Wald | df | P | HR | 95% CI | |
| Age | 0.083 | 0.03 | 7.934 | 1 | 0.005 | 1.087 | 1.026 | 1.152 |
| Gender | -0.26 | 0.363 | 0.516 | 1 | 0.473 | 0.771 | 0.379 | 1.569 |
| BMI | 0.057 | 0.069 | 0.671 | 1 | 0.413 | 1.058 | 0.924 | 1.212 |
| Stage | 1.184 | 0.243 | 23.665 | 1 | 0 | 3.267 | 2.028 | 5.263 |
| Diabete | 0.35 | 0.381 | 0.843 | 1 | 0.359 | 1.418 | 0.673 | 2.992 |
| Hypertension | -0.046 | 0.392 | 0.014 | 1 | 0.906 | 0.955 | 0.443 | 2.058 |
| OCTN2 | 1.767 | 0.376 | 22.14 | 1 | 0 | 5.855 | 2.804 | 12.225 |
| LINCMD1 | 2.858 | 0.613 | 21.749 | 1 | 0 | 17.426 | 5.243 | 57.92 |

β: coefficient; SE: standard error; Wald: Wald statistic; df: degrees of freedom; P: P-value; HR: hazard ratio; 95% CI: 95% confidence interval

| **Supplementary Table 6. The primer sequences used for plasmid construction** | | |
| --- | --- | --- |
| Gene | Sequence (5’-3’) | |
| Human: | | |
| OCTN2 | F: | ATGCGGGACTACGACGAGG |
|  | R: | GAAGGCTGTGCTTTTAAGGATTGTGG |
| OCTN2-sh1 | GCAGATCTTCTCGAAGAATTT | |
| OCTN2-sh2 | CGAGTGAGTTACAAGACCTAA | |
| OCTN2-sh3 | CCAATGGGATTGTTGTGCCTT | |
| CRAT-sh1 | GTCATCGAGTACACGAAGAAA | |
| CRAT-sh2 | CGTGGTACACAACTACCAGTT | |
| CRAT-sh3 | CAAGGCATACAACACCCTCAT | |
| DZIP3 | F: | ATGGATTCTCTACCAGATGAATTTTTGTGAGG |
|  | R: | TCAGATCTTGGGCAACTGCCG |
| DZIP3-sh1 | CGGAAATTGAAGGATGCTTAT | |
| DZIP3-sh2 | CCATACTTACTGTTCCTCAAA | |
| DZIP3-sh3 | CGATTTGTAGTTACCTAGATT | |
| LINCMD1 | F: | ATGAGGAAACCCAGGCCTGG |
|  | R: | ATTCCATACAGAGCTCCCAAGCTG |
| LINCMD1-sh1 | ATCTCAACAGCACTCACAAAT | |
| LINCMD1-sh2 | AGGCTTTCCAGACAGGATATT | |
| LINCMD1-sh3 | AGGAAACACAGAATGAGTTTA | |
| HECTD4 | F: | CATGGCGTCTCACTGACAGTTGG |
|  | R: | TCAGCCACTGAGGGGGTCTTC |
| HNRNPUL1 | F: | ATGCGATCCTGGGCGTTCTC |
|  | R: | CTACTGTGTACTTGTGCCACCCTG |
| MATR3 | F: | ATGTCCAAGTCATTCCAGCAGTCATCTC |
|  | R: | TTAAGTTTCCTTCTTCTGTCTGCGTTCTTCTG |
| ATXN2 | F: | ATGTCGCTGAAGCCCCAGC |
|  | R: | TTACAACTGCTGTTGGTGGTGGG |
| MOV10 | F: | ATGCCCAGTAAGTTCAGCTGCC |
|  | R: | TCAGAGCTCATTCCTCCACTCTGG |
| PRRC2A | F: | GAGCTTCCAGCGCAATGTCC |
|  | R: | AGAGGAACTCCCTCAGCGG |
| HERC5 | F: | AGTAGCTGAGGCTGCGGTT |
|  | R: | TCAGCCAAATCCTCTGTTGTTGTTGA |
| KHSRP | F: | ATGTCGGACTACAGCACGGG |
|  | R: | TTTAACCTCTGGACCCAGCGAATG |
| CDC5L | F: | ATGCCTCGAATTATGATCAAGGGGG |
|  | R: | TCAGAATTTTGACTTTAAAGTCTCTTTCTCCAGC |
| RBM14 | F: | ATGAAGATATTCGTGGGCAACGTCG |
|  | R: | CTGCGACTCTGATAAACGGCG |
| SFPQ | F: | ATGTCTCGGGATCGGTTCCG |
|  | R: | CTAAAATCGGGGTTTTTTGTTTGGGCC |
| ZC3H4 | F: | GCTTTCGGAACTATGGAGGCCG |
|  | R: | ATGCCACCCTAGGAAGGGTG |
| HNRNPU | F: | ATGAGTTCCTCGCCTGTTAATGTAAAAAAGC |
|  | R: | TCAATAATATCCTTGGTGATAATGCTGACTCCATG |
| USP10 | F: | TTGAAACATCATGCCCTGGTTGC |
|  | R: | TTACAGCAGGTCCACTCGGC |
| DSC3 | F: | ATGGCCGCCGCTGG |
|  | R: | TTATCTCTTTGTGCATGCTTCTGCTAATG |
| NDUFA6-DT | F: | GCGCATGCTCATCTACAGAAACG |
|  | R: | CCTTTTGAACCTTTTTATTTTCCTGGCAGG |
| LINC01960 | F: | CTCTGGTCTCCTGACTCTGG |
|  | R: | TTATATTTCACCCTTAAATACT  TTAATATACACCATTTAAAAG |
| RUSC1-AS1 | F: | CACAGAGGGTGTGACCCAAAGC |
|  | R: | GAATGTTAGAGTGTTTTATTAAT  TTCTTTGTCAGACAAGTGTTTAG |
| SENCR | F: | TTTTGCTCAGGGCCTGTGCG |
|  | R: | TTCCACAGTTAAAAACTAAAGG  GCAAGTCTTCAATTTAATCTAG |
| Mouse | | |
| *Octn2* | F: | ATGCGGGACTACGACGAGG |
|  | R: | TTAGAAGGCTGTGCTCTTTAGGACTG |
| sh*Octn2-*sh1 | GCTAAGGGTCAAAGGAATAAA | |
| sh*Octn2-*sh2 | CCAAGTGAGTTACAAGACTTA | |
| sh*Octn2-*sh3 | GATCGCTTCCTGCCTTATATT | |

| **Supplementary Table 7. Antibodies list** | | |
| --- | --- | --- |
| Antibodies | Source | Identifier |
| Anti-OCTN2 | Proteintech | 16331-1-AP |
| Anti-OCTN2 | Abcam | ab180757 |
| Anti-CPT2 | Proteintech | 26555-1-AP |
| Anti-CPT1A | Proteintech | 66039-1-Ig |
| Anti-CACT | Proteintech | 19363-1-AP |
| Anti-TMLHE | Proteintech | 16621-1-AP |
| Anti-BBOX1 | Proteintech | 16099-1-AP |
| Anti-CRAT | Proteintech | 15170-1-AP |
| Anti-p53 | Proteintech | 60283-2-Ig |
| Anti-Acetyl-p53 (Lys382) | Cell signaling technology | 2525 |
| Anti-Acetyl-p53 (Lys373) | Affinity | AF4363 |
| Anti-Acetyl-p53 (Lys320) | Immunoway | YK0051 |
| Anti-PUMA | Proteintech | 55120-1-AP |
| Anti-BAX | Proteintech | 50599-2-Ig |
| Anti-GADD45A | Proteintech | 13747-1-AP |
| Anti-P21 | Proteintech | 10355-1-AP |
| Anti-TAP1 | Proteintech | 11114-1-AP |
| Anti-HLA-A | Proteintech | 15240-1-AP |
| Anti-PSMB8 | Proteintech | 14859-1-AP |
| Anti-B2M | Proteintech | 13511-1-AP |
| Anti-Acetyl-Histone H3 (Lys9) | Proteintech | 29133-1-AP |
| Anti-Acetyl-Histone H3 (Lys27) | Proteintech | 82902-1-RR |
| Anti-Histone H3 | Proteintech | 17168-1-AP |
| Anti-DZIP3 (Rabbit pAb) | Affinity | DF4017 |
| Anti-DZIP3 (Mouse mAb) | Santa Cruz | sc-514725 |
| Anti-Acetylated-Lysine | Cell signaling technology | #9814 |
| Anti-Phospho-(Ser/Thr) | Abcam | ab117253 |
| Anti-Ki-67 | Proteintech | 27309-1-AP |
| Anti-CD11b | Proteintech | 66519-1-Ig |
| Anti-CD11b | Proteintech | 31745-1-AP |
| Anti-IgG | Proteintech | 30000-0-AP |
| Anti-β-ACTIN | Abclonal | AC026 |
| Anti-HA (Mouse mAb) | Medical & Biological Laboratories | M180-3 |
| Anti-HA (Rabbit mAb) | Cell Signaling Technology | 3724 |
| Anti-Flag (Mouse mAb) | Medical & Biological Laboratories | M185 |
| Anti-Flag (Rabbit pAb) | Cell Signaling Technology | 14793 |
| Anti-Myc (Mouse mAb) | Abclonal | AE010 |
| Anti-Myc (Rabbit mAb) | Cell Signaling Technology | 2278 |
| Anti-Ub-K48 | Abcam | ab140601 |
| Anti-Ub | Abcam | ab134953 |
| HRP-conjugated Goat Anti-Rabbit IgG(H+L) | Proteintech | SA00001-2 |
| HRP-conjugated Goat Anti-Mouse IgG(H+L) | Proteintech | SA00001-1 |
| Multi-rAb™ Polymer HRP-Goat Anti-Mouse Recombinant Secondary Antibody (H+L) | Proteintech | RGAM011 |
| Multi-rAb® Polymer HRP-Goat Anti-Rabbit Recombinant Secondary Antibody (H+L) | Proteintech | RGAR011 |
| Goat anti-Mouse IgG(H+L), Alexa Fluor 488 | Invitrogen | A11029 |
| Goat anti-Rabbit IgG(H+L), Alexa Flour® 488 | Invitrogen | A11034 |
| Goat anti-Mouse IgG(H+L), Alexa Fluor® 568 | Invitrogen | A11031 |
| Goat anti-Rabbit IgG(H+L), Alexa Fluor 568 | Invitrogen | A11036 |

| **Supplementary Table 8. The primer sequences used for qRT-PCR** | | | |
| --- | --- | --- | --- |
| Specie | Gene | Sequence (5’-3’) | |
| Human | TMLHE | F: | GTGCTTGGCAGCAACATGAA |
|  |  | R: | GACTGGCCCAATCCCAATCA |
|  | OCTN2 | F: | GGATGGGAAAGAAGCCTGCT |
|  |  | R: | AGCACACCCACGAAGAACAA |
|  | BBOX1 | F: | AGACAACTGTCCGTGCTCTG |
|  |  | R: | TCTTCAGCCAATCAGCCTGG |
|  | CPT1A | F: | CCAGACGAAGAACGTGGTCA |
|  |  | R: | AACGTCACAAAGAACGCTGC |
|  | CPT2 | F: | CCGTCCACTTTGAGCACTCT |
|  |  | R: | GCCATGGTACTTGGAGCACT |
|  | CACT | F: | CTCTGGACACGGTCAAGGTC |
|  |  | R: | CTCCTTCATCCCGGATCAGC |
|  | P21 | F: | GCCCAAGCTCTACCTTCCC |
|  |  | R: | GGAGTGGTAGAAATCTGTCATGC |
|  | BBOX1 | F: | AGACAACTGTCCGTGCTCTG |
|  |  | R: | TCTTCAGCCAATCAGCCTGG |
|  | PUMA | F: | ACCTCAACGCACAGTACGAG |
|  |  | R: | TAAGGGCAGGAGTCCCATGA |
|  | BAX | F: | TTTGCTTCAGGGTTTCATCC |
|  |  | R: | GTTGAAGTTGCCGTCAGA |
|  | GADD45 | F: | ATCACTGTCGGGGTGTACGA |
|  |  | R: | CTGCAGAGCCACATCTCTGT |
|  | TNF-α | F: | GTGACAAGCCTGTAGCCCAT |
|  |  | R: | CAGACTCGGCAAAGTCGAGA |
|  | IL-1α | F: | TCTTCTGGGAAACTCACGGC |
|  |  | R: | GCACACCCAGTAGTCTTGCT |
|  | IL-6 | F: | ACCCCCAGGAGAAGATTCCA |
|  |  | R: | ATTTGTGGTTGGGTCAGGGG |
|  | P53 | F: | ACCTATGGAAACTACTTCCTGAAA |
|  |  | R: | ACCATCGCTATCTGAGCAGC |
|  | TAP1 | F: | CTAGCTCTAGGTGTCCCGCT |
|  |  | R: | ACTGACAACGAAGGCGGTAG |
|  | HLA-A | F: | TCACAGACTGACCGAGCGAA |
|  |  | R: | CCAGGTCAGTGTGATCTCCG |
|  | PSMB8 | F: | AGATGACACGACCCTACCCA |
|  |  | R: | AACGTTCCTTTCTCCGTCCC |
|  | B2M | F: | AGATGAGTATGCCTGCCGTG |
|  |  | R: | GCGGCATCTTCAAACCTCCA |
|  | DZIP3 | F: | GCCTTTCCACAGCAAACCAG |
|  |  | R: | GAGCGCAAGGCAAAACTGAA |
|  | CRAT | F: | AGCAAGACCAAGAAGCCTCC |
|  |  | R: | GATCTGATCCGCAGTGAGGG |
|  | LINCMD1 | F: | TGGCCTTCACACTCCATGTC |
|  |  | R: | ACATCGTGAAGACTGGCAGG |
|  | β-ACTIN | F: | CATGTACGTTGCTATCCAGGC |
|  |  | R: | CTCCTTAATGTCACGCACGAT |
| Mouse | *P21* | F: | CCCGAGAACGGTGGAACTTT |
|  |  | R: | AGAGTGCAAGACAGCGACAA |
|  | *Puma* | F: | ACCTCAACGCGCAGTACGA |
|  |  | R: | TGAGGGTCGGTGTCGATGCT |
|  | *Gadd45a* | F: | CTGCAGAGCAGAAGACCGAA |
|  |  | R: | GGGTCTACGTTGAGCAGCTT |
|  | *Bax* | F: | CTGGATCCAAGACCAGGGTG |
|  |  | R: | CCTTTCCCCTTCCCCCATTC |
|  | *Octn2* | F: | TCCGAACACGGAATATCAGG |
|  |  | R: | AGCCCACTGATATGGTCAGC |
|  | *Tap1* | F: | GGACTTGCCTTGTTCCGAGA |
|  |  | R: | ATAGCGAAGGCATCTGGACG |
|  | *Hla-a* | F: | CAATGAGGCTCCTCAAGCGA |
|  |  | R: | GTCACGGTTGACGAAGAAGC |
|  | *Psmb8* | F: | ACTACAGTTTCTCCGCGCAA |
|  |  | R: | AAAGGACCTCAGGAATGCGG |
|  | *B2m* | F: | GACCGGCCTGTATGCTATCC |
|  |  | R: | CAGTAGACGGTCTTGGGCTC |
|  | *Lincmd1*-FL | F: | CTCTTTGCAGTGGGACAGC |
|  |  | R: | GCTATGATGGCAAAACCAGCA |
|  | *Lincmd1*-F1 | F: | CTCTTTGCAGTGGGACAGC |
|  |  | R: | AGGCAGGTCCCTCCTCCT |
|  | *Lincmd1*-F2 | F: | GCAACTTGTCCCCAGGCTT |
|  |  | R: | GCTATGATGGCAAAACCAGCA |
|  | *Lincmd1*-F3 | F: | GCAACTTGTCCCCAGGCTT |
|  |  | R: | CAAACCTCTCAGGAAGACGGA |
|  | *Lincmd1*-F4 | F: | GTCCCCTTCAACCAGCTACA |
|  |  | R: | GCTATGATGGCAAAACCAGCA |
|  | *β-actin* | F: | GTGACGTTGACATCCGTAAAGA |
|  |  | R: | GCCGGACTCATCGTACTCC |

| **Supplementary Table 9. The primer sequences used for dual-luciferase reporter assay** | | |
| --- | --- | --- |
| Gene | Sequence(5’-3’) | |
| P21 | F: | CTAGCTAGCTAGCTTCCCAGGAACATGCTTG |
|  | R: | GAAGATCTTCCTGACTTCGGCAGCTGCTC |
| GADD45A | F: | GAACTAACGTTAAATGTGCAATATGTTAGAATTCATGC |
|  | R: | GGAGGACAAAGGGCCCCG |
| BAX | F: | CTAGCTAGCTAGAACAGAAAAGCAGGCCTGG |
|  | R: | GAAGATCTTCAATCGCAGCTCTAATGCCTTC |
| PUMA | F: | GGGTCCCTGAACCCCGAGGA |
|  | R: | CACCCCGGGGGCATGAACAC |

| **Supplementary Table 10. Online Tools and Databases Used in This Study** | | | |
| --- | --- | --- | --- |
| Tool/Database Name | Website URL | |  |
| GEPIA | <https://gepia.org/> | |  |
| iUUCD | [http://iuucd.biocuckoo.org](http://iuucd.biocuckoo.org/) | |  |
| STRING | <https://string-db.org/> | |  |
| BioGRID | <https://thebiogrid.org/> | |  |
| IntAct | <https://www.ebi.ac.uk/intact/> | |  |
| RPISeq | http://pridb.gdcb.iastate.edu/RPISeq/ |  |  |
